# Evidence for Ussurian tube-nosed bats (*Murina ussuriensis*) hibernating in snow

**DOI:** 10.1038/s41598-018-30357-1

**Published:** 2018-08-13

**Authors:** Hirofumi Hirakawa, Yu Nagasaka

**Affiliations:** 1 Forestry and Forest Products Research Institute, Hitsujigaoka 7, Toyohira, Sapporo 062-8516 Japan; 2 Forestry Research Institute, Hokkaido Research Organization, Bibai, 079-0198 Japan

## Abstract

Surviving winter is a challenge for endothermic animals living at high latitudes. In bats, some species migrate to milder climates in winter, but others presumably stay and hibernate in thermally buffered roosts. However, we know little about where, or in what roosts bats hibernate. Ussurian tube-nosed bats (*Murina ussuriensis*) have occasionally been observed under or near the surface of snow. We collected the details of those accounts and used our own observations to conclude that these bats hibernate in snow. To our knowledge, this is the first evidence of hibernation in snow for bats, and second for mammals, following polar bears (*Ursus maritimus*) denning in snow.

## Introduction

Bats are one of the most successful groups of mammals, comprising more than 1,300 species. They range widely from the tropics to the sub-Arctic^1,2^. Surviving winter is a challenge for bats living at high latitudes. Some bats, like birds, migrate to milder climates in winter, but many do not^3,4^. Non-migratory species are, unlike birds, assumed to stay and hibernate in roosts buffered from extreme conditions because food (insects) is rarely if ever available. However, we do not know the details of where these bats hibernate, let alone the roosts they use^5^. We often assume that bats with unknown roosts hibernate in tree cavities or rock crevices^6^. Herein, we show that Ussurian tube-nosed bats (*Murina ussuriensis*; Chiroptera: Vespertilionidae) hibernate in snow.

**Figure 1:**
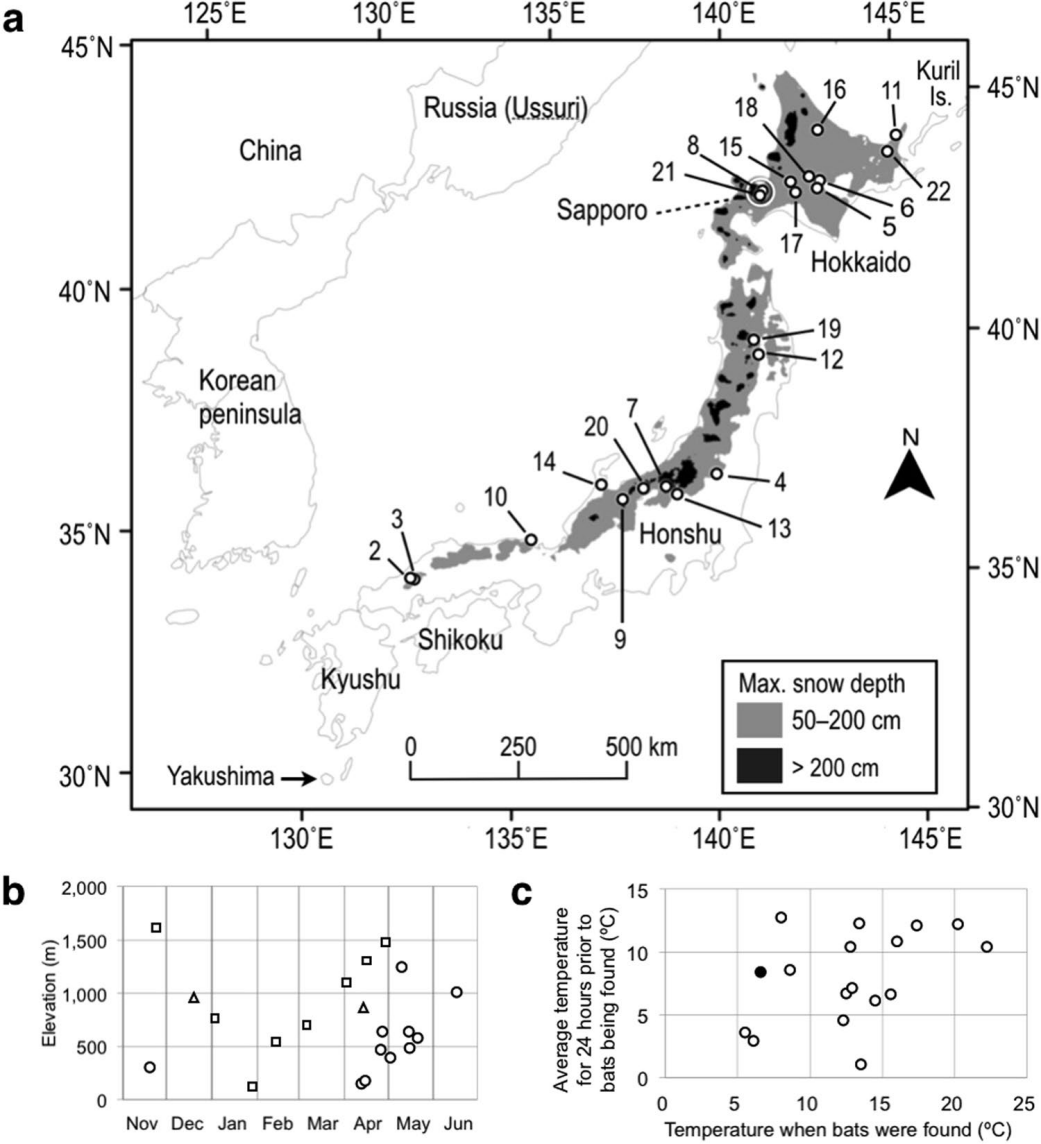
Where, when, and in what conditions bats were found in snow. Of 22 collected accounts, the earliest one in Sapporo 1964 was omitted from figures due to unknown details. (**a**) The sites where bats were found in snow. We numbered accounts chronologically, which corresponds to Supplementary Note and Supplementary Dataset S1. (**b**) The elevation and season of observations in Hokkaido (circles), northern Honshu (triangles), and central and western Honshu (squares). (**c**) Temperature conditions for observations made on snowmelt. The solid circle represents an observation made on 20 November 2011; open circles those in spring (Supplementary Fig. S1, S2). The temperature value of the vertical axis in °C approximates the reduction in snow depth in cm for 24 hours before the bats were found (see Methods).

The Ussurian tube-nosed bat is a small (4–8 g) insectivorous species occurring in the Kurile Islands, Sakhalin, south-eastern Siberia, Korea, and Japan^7^. Its population within Japan, which some authors^8,9^ have called *M*. *silvatica*, ranges from Hokkaido in the north to Yakushima in the south (Fig. 1). From spring to autumn, individuals usually roost in the dead foliage of trees^10–12^. During other times of year, they also roost in tree cavities^13,14^, but their whereabouts during winter remain unknown in areas where subfreezing temperatures are the norm.

In 2005 we came across some anecdotal accounts of *M*. *ussuriensis* being found near the surface of residual piles of snow in spring. We contacted the authors of these accounts for precise details. We searched for additional accounts with various means and asked for the same details (Methods). We also tried to observe the phenomenon ourselves and in 2013 we first found bats in snow. To date, we have collected 22 accounts and found 37 individuals. We analysed these data to examine whether *M*. *ussuriensis* hibernates in snow.

## Results

**Figure 2:**
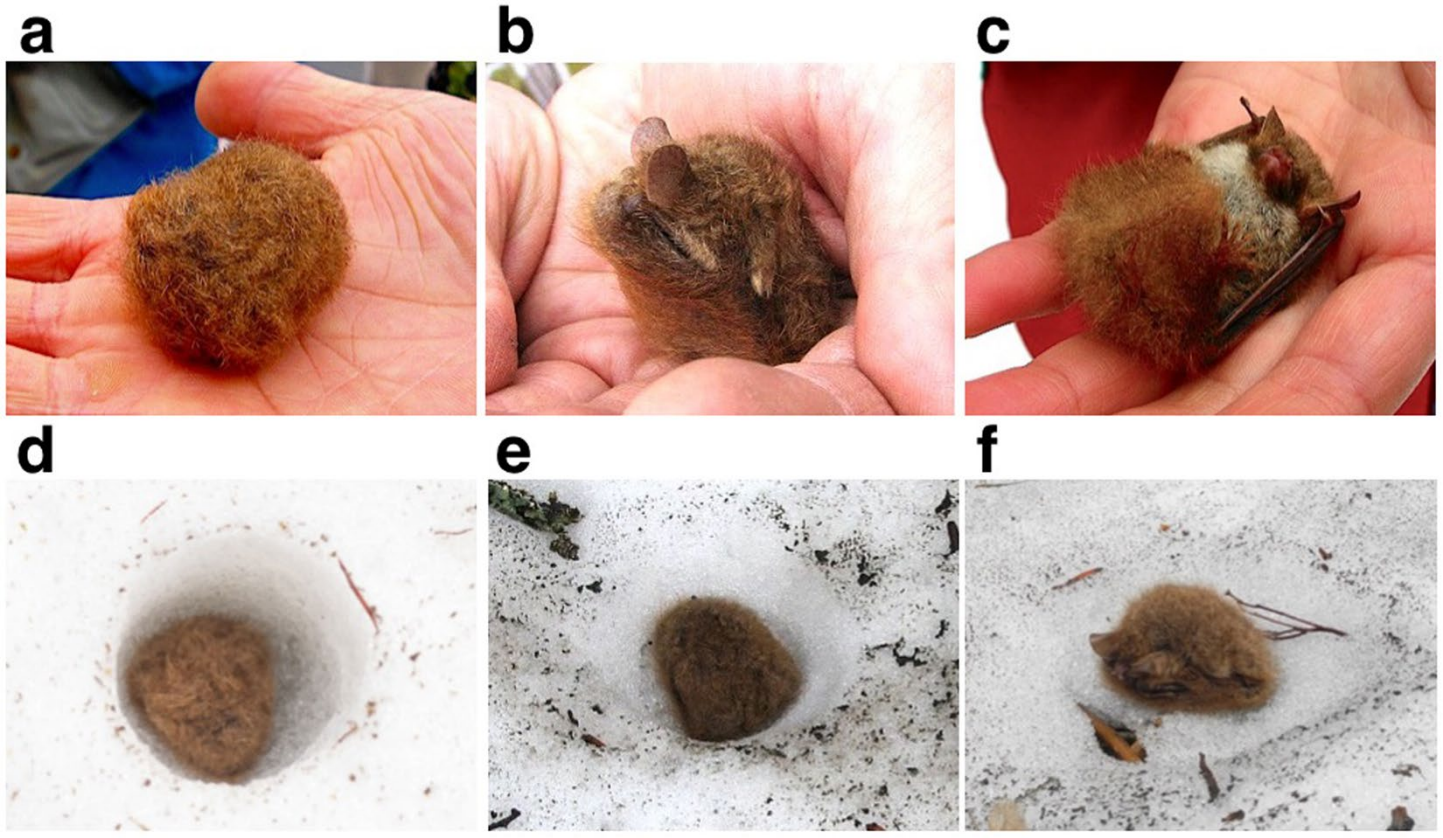
Postures of the bats and the states of snow surrounding them. (**a**–**c**) The postures: Spherical posture, with the bat’s head completely tucked under its tail membrane (**a**); Semi-spherical posture, with body elongated and ears protruded (**b**) or head and upper abdomen exposed (**c**). The last posture may only appear after disturbance. These photos were taken by people who picked up the bats without knowing they were actually bats. (**d**–**f**) The shapes of holes and dents. Herein, holes are cylindrical with the rim almost right-angled (**d**); dents are cone-shaped with the rim being wider (**e**); almost flat surface (**f**). Photos were provided under a CC BY license by Eiichi Kawashima (**a**,**c**), Akihiro Kawamura (**b**,**e**), Yushi and Keiko Osawa (**d**), and Yu Nagasaka (**f**).

First we analysed 22 accounts (Supplementary Note; Supplementary Dataset S1; Supplementary KML S1). These were from heavy snow areas (>50 cm at maximum depth) on Honshu and Hokkaido (Fig. 1a). The seasonally earliest account was from 20 November and the latest 17 June; four accounts were from snow-accumulating season, one from mid-winter, and 17 from snowmelt season (Fig. 1b; Supplementary Table S1; Supplementary Figs S1 and S2). One account from snowmelt season involved three bats, whereas all other accounts involved single individuals. All accounts found bats alive but behaviourally lethargic in spherical or semi-spherical postures (Fig. 2a–c; these photos were taken by people who picked up the bats without knowing they were actually bats). Five accounts determined sex: three were females and two males. Three accounts weighted bats a few days later: two were alive at 4.0 g and 3.8 g, and the other at 5.0 g may have been dead.

All the accounts from snowmelt season found bats on a dent or in a hole on the surface of snow (Fig. 2d–f). Holes were typically cylindrical in shape, while dents were cone shaped with wider rims. The maximum depth of holes was 6–7 cm. Many of these accounts found bats on warm days (e.g., daytime high > 10 °C) shortly before the disappearance of snow (Fig. 1c; Supplementary Figs S1 and S2). In contrast, of the earlier accounts until mid-winter, four reported bats excavated or exposed from snow by human activities and one on 20 November found a bat when the first substantial snowfall of the season had almost melted away several days later (see Yubari account in Supplementary Fig. S1).

**Figure 3:**
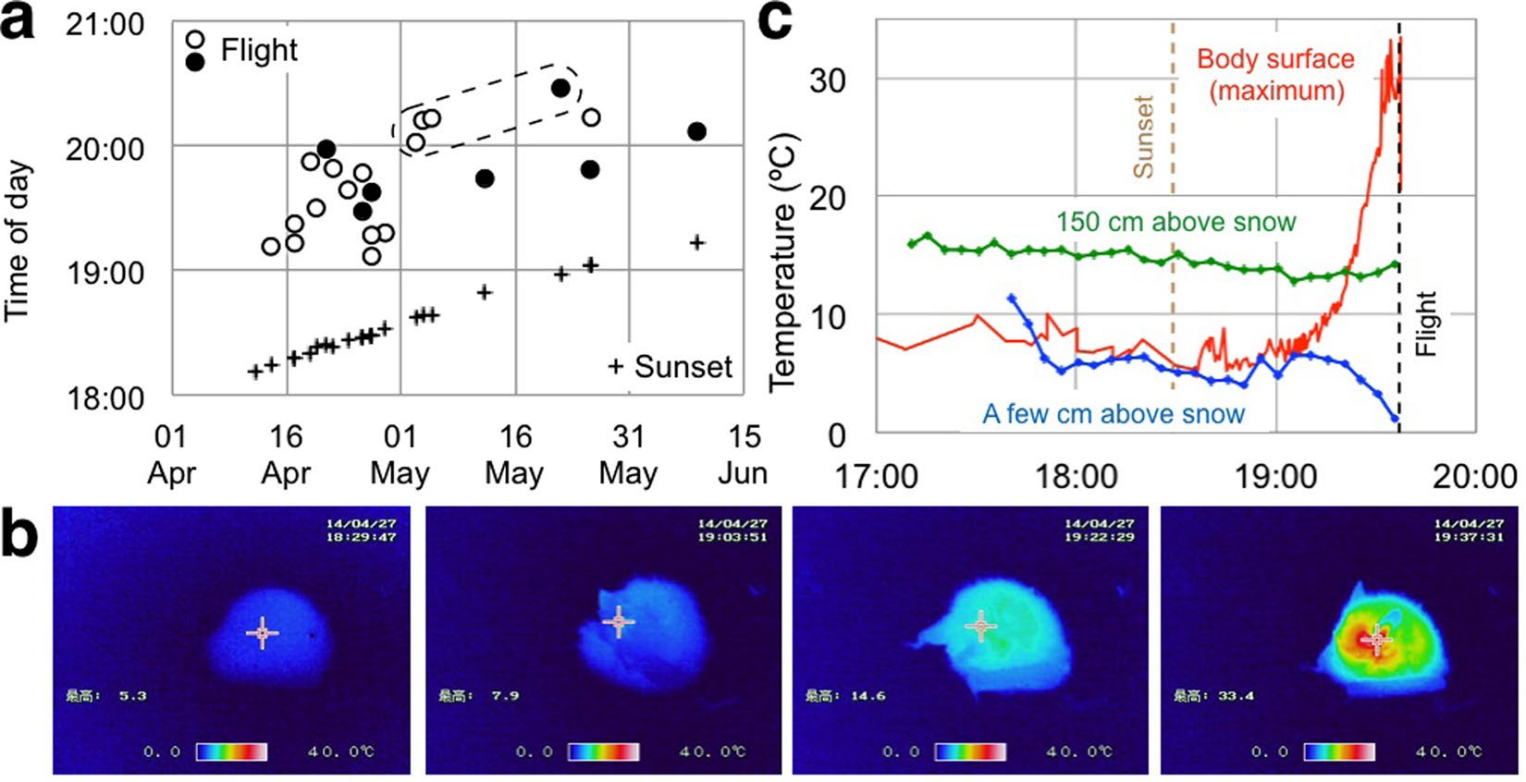
Authors’ post-sunset observations of exposed bats. (**a**) Of 23 bats we observed after sunset, all but one took flight in 38–94 minutes (Supplementary Table S2). We observed eight of those (solid circles) by thermography (Supplementary Fig. S3). Four (encompassed by dashed line) were wet from rain (Supplementary Fig. S4). (**b**) Thermographic images of a bat taken at 0, 34, 53, and 66 min after sunset on 27 April 2014 (Supplementary Slideshow S1). (**c**) The change in temperatures measured on 27 April 2014.

Since 2013, we found 37 bats in over 300 hours (156 days) of targeted searches near Sapporo, Hokkaido (Supplementary Table S2; Supplementary Dataset S2; Supplementary Note; Supplementary KML S2; Supplementary Videos S1–S4). One bat was dead. Of 23 individuals that we observed after sunset, 22 took flight within 94 minutes after sunset (Fig. 3a). Thermographic observation of eight of those bats showed that they increased body temperature (*T*_b_, hereafter) prior to flying away (Fig. 3; Supplementary Fig. S3; Supplementary Slideshow S1). Four bats that took flight were wet by rain (Fig. 3a; Supplementary Fig. S4).

## Discussion

Our data suggested the possibility of bats hibernation in snow. However, we have not confirmed that individual bats survive entire winters in snow. To settle the issue, we examined how snow holes and dents may form. If bats hibernate in snow, cavities accommodating bats inside snow are exposed by snowmelt as holes, and then become dents (cavity-exposure hypothesis; Supplementary Fig. S5a). Alternatively, bats that land on snow after hibernating elsewhere might have created holes and dents by melting into it (sit-and-sink hypothesis; Supplementary Fig. S5b). Is the latter possible?

**Figure 4:**
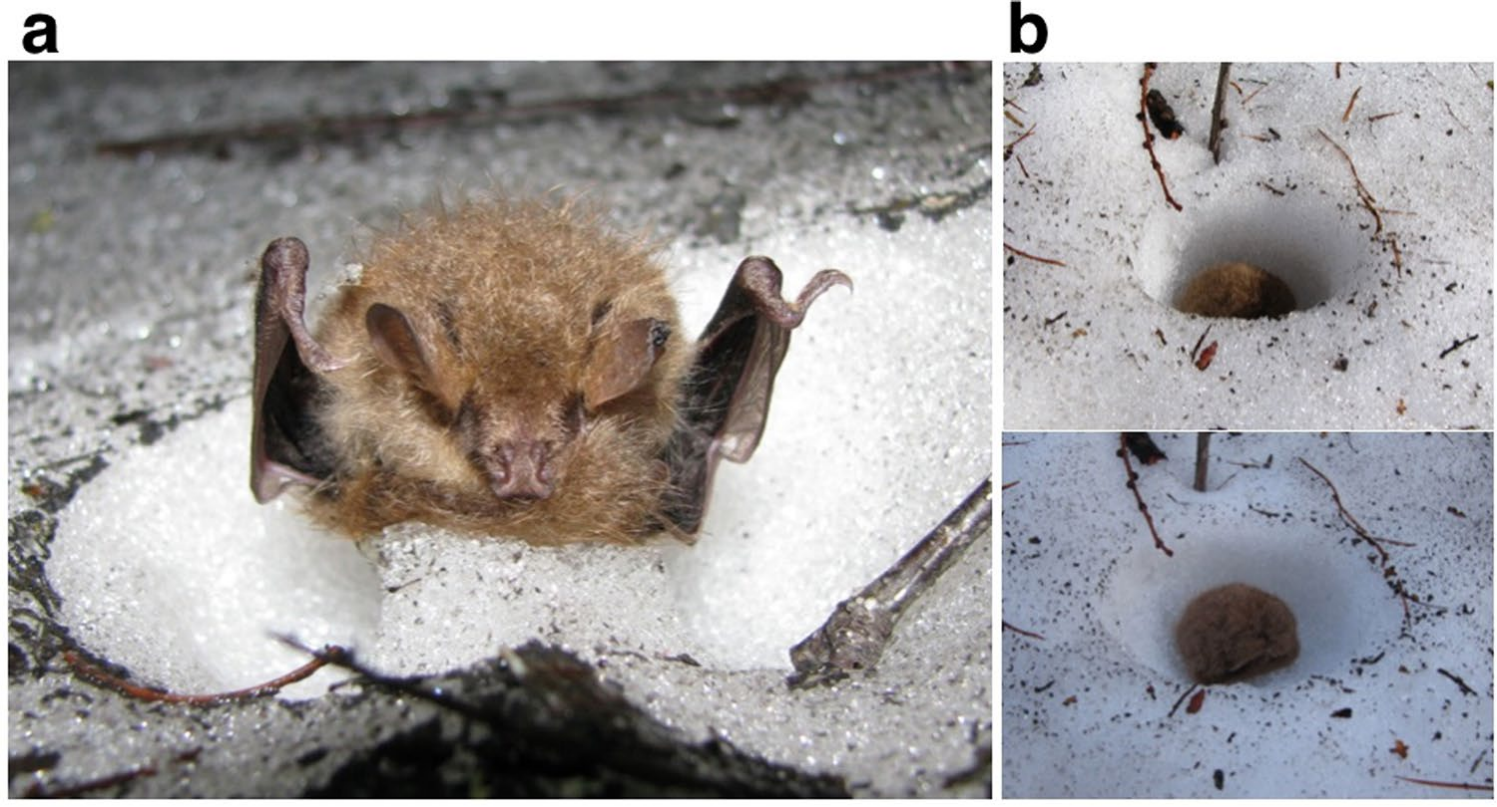
Authors’ observations of the snow under and around bats. (**a**) A snow pillar formed under a bat found dead on 31 May 2013 suggests that the snow directly under torpid, hypothermic bats is more difficult to melt than the snow around them: evidence against sit-and-sink hypothesis. (**b**) The change of the snow likely represents part of the process of an exposed cavity getting shallower over time: evidence for cavity-exposure hypothesis. The upper and lower photos were taken at 15:10 and 17:59 on 24 April 2017, respectively, with 2 h 49 min elapsed in between them. The estimated air temperatures were 14.2 °C and 11.5 °C, respectively. The sunset was at 18:27. This bat took flight at 19:38, 71 minutes after sunset (Supplementary Video S4).

First we assessed how fast snow melted when bats were found during snowmelt season as a background process. Because daily average air temperatures (*T*_a_, hereafter) in °C approximate daily reductions in snow depth in cm (Methods), the vertical axis of Fig. 1c indicates the reductions of snow depth in cm that had occurred for 24 hours before these bats were found. This reduction occurs mostly by surface melting. The sit-and-sink hypothesis predicts that snow under bats melted faster than this speed of surface melting. We calculated (see Methods) that to sink 6 cm into snow, a bat would need to remain euthermic (*T*_b_ = 37 °C) for at least 6.4 hours, and require 8.9–14.0 kJ of energy, equivalent to 0.23–0.36 g of body fat, or 5–7% of body mass. However, it seems unlikely that a heterotherm, which saves energy by lowering its metabolism and *T*_b_ during inactivity, would remain euthermic for such a long time. If a bat’s *T*_b_ fell below euthermic levels, sinking 6 cm would take much longer, particularly if concurrent surface melting counteracted the sinking effect. All but one of the bats found in our study were lethargic (for the exception, see Supplementary Video S3) and likely torpid at *T*_b_ well below euthermic levels. Using thermography we actually observed low body surface temperatures of bats upon discovery, followed by rapid post-sunset increases to euthermic levels. The dead bat found on a snow pillar further indicated faster melting of snow around than directly below a non-metabolizing body (Fig. 4a). Thus, living bats that forego metabolic warming do not likely sink into snow. Our observations of holes becoming progressively shallower over time (Fig. 4b) further oppose the site-and-sink hypothesis. We thus concluded that bats found in snowmelt season had hibernated in snow.

**Figure 5:**
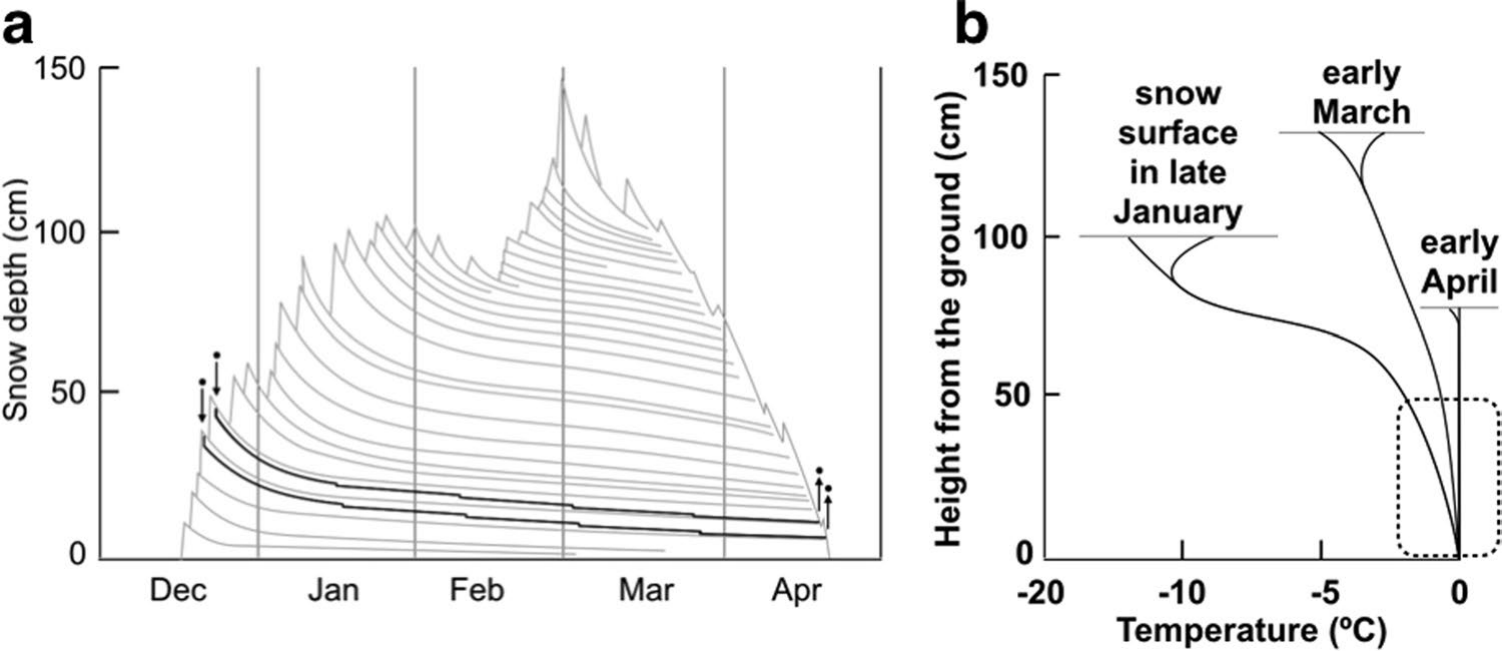
Schematic change in the structure and temperature profile of snow cover with plausible positions of hibernating bats. (**a**) Intermittent precipitation events make layers in snow cover, which gradually become compacted; in spring, melt occurs mostly at the surface. (This part of the figure was adapted from a figure in the literature^50^, which describes snow cover in Sapporo during the winter of 1954–1955). Bats (dots) settle in snow at an early stage of snow cover; periodic arousals to warm body temperatures lead to snow melt and the bat lowering through the snow layers (thick lines); they are exposed shortly before the disappearance of snow cover. (**b**) The temperature near the top surface of snow cover is strongly affected by fluctuating air temperatures; in contrast, the temperature at the bottom is kept near 0 °C^15,44,45^. Because of the low thermal conductivity of snow, temperature is stable in lower parts of the snow cover (encircled by dashed line).

A plausible scenario is as follows. In late autumn when night *T*_a_ falls below 5 °C, bats change roosts from foliage to tree cavities^14^, where they probably use multi-day torpor. During a subsequent arousal, individuals that detect substantial snow cover alight upon it (Fig. 5a). Snow surfaces are softer and lighter at this time of the year^15^, so the bats easily make a small cavity in the snow. A bat’s duration of confinement would depend on how much snow further accumulates above the bat and how long the snow takes to melt. Thus, it may last only a few days (e.g., see Yubari account in Supplementary Fig. S1), or continue for possibly longer than half a year (e.g., see Uenshiri account in Supplementary Fig. S2). During confinement, bats periodically arouse to euthermic *T*_b_, as is common in other hibernating species^16^. The raised *T*_b_ melts the snow in contact with the bats, thus lowering their position in snow and making the snow cavity cylindrical. However, the increase in cavity height would later be diminished due to snow compaction to some degree. Surface snow melting eventually exposes the cavities. Exposure could occur during night or day but should be more frequent during daytime due to faster snowmelt conditions. When exposure occurs during daytime, bats likely remain torpid until after sunset and then arouse to fly away. They may then roost in tree cavities until foliage becomes available.

As indicated above, we suppose that *M*. *ussuriensis* uses snow only opportunistically. They would not fly for extended periods or long distances to seek suitable snow cover, and in areas or years with milder winters and limited snow, they would remain in tree cavities throughout the hibernation period. Opportunistic entry into snow may result in only temporary confinement if snow melts soon after, as in Yubari account; however, through early snow entry bats could secure a position near the ground where temperatures are stable and close to 0 °C (Fig. 5b). We have no idea about how bats assess the amount of snow before entering it, but suppose that they may use cues such as the amount of vegetation protruding from the snow surface. By the time that we suspect bats enter snow in our study area, so much snow typically covers the ground that most herbaceous vegetation is buried, even thick and tall bamboo grass (see Supplementary Video S2).

We predict that hibernation in snow is common in *M*. *ussuriensis*, as this behaviour bears three possible benefits: (1) conservation of water^17,18^, (2) safety from predators, and (3) thermal stability. First, water loss from body surfaces or through respiration should be minimal in small snow cavities because moisture easily saturates them. This could lead to prolonged torpor bouts and savings of the energy required for arousals^19^. Also, rehydration at arousals is easy. Second, predators are unlikely to search prey inside snow because prey is rare, visual search is useless, and movement is energetically expensive. Bats seem more vulnerable when exposed on the surface of snow by snowmelt. However, even exposed bats still seem difficult for predators to detect (Supplementary Fig. S6) and such exposure lasts no longer than half a day (mostly much shorter) because bats take flight after dark. Third, in contrast to tree cavities that have fluctuating temperatures that range across 0 °C in areas where sub-zero ambient temperatures (*T*_a_) are the norm^20–22^, snow cavities near the ground level have stable temperatures close to 0 °C (Fig. 5b). Hibernating in snow is viable and even beneficial if *M*. *ussuriensis* can exploit such stable but freezing conditions.

Most vespertilionid bats cannot withstand freezing conditions and metabolically warm themselves when *T*_a_ approaches freezing. However, laboratory experiments showed that certain species of tree-roosting bats in North America (*Lasiurus spp*.) survived subfreezing *T*_a_ while torpid and withstood sub-zero *T*_b_, a state called “super-cooling”^23–25^. It is unknown, however, how long these species can endure in a super-cooled state because researchers rewarmed all the bats tested in less than half a day, presuming that super-cooling was only a transient state in which spontaneous arousals were impossible. We predict that *M*. *ussuriensis* hibernating in snow super-cools because this tiny endotherm probably cannot afford to metabolically keep its *T*_b_ above freezing in snow for months. We further predict that infrequent but spontaneous arousals interrupt this torpor because without them cylindrical cavities would not form. Such behaviour — super-cooling with infrequent spontaneous arousals — is observed in arctic ground squirrels (*Spermophilus parryii*) hibernating underground^26^.

Ours appear to be the first evidence of bats hibernation in snow, and the second for mammals, following polar bears (*Ursus maritimus*) denning in snow^27,28^. However, bears, including *U*. *maritimus*, do not hibernate like other mammalian hibernators; they maintain *T*_b_ above 30 °C and females give birth and nurse young during denning in winter^29–31^. Other species of bats (or mammals) might also hibernate in snow. Eastern red bats (*L*. *boreali*s), known to super-cool, sometimes hibernate under leaf litter and become temporarily buried under snow^32,33^—behaviour possibly a precursor to snow hibernation (or “sheltering”). The hoary bat (*L*. *cinereus*) might also at least temporarily shelter in snow; a hiker’s dog pulled a torpid *L*. *cinereus* from snow in the Santa Catalina Mountains, Arizona on 27 December 2008 (32.40740 N, 110.75102 W, 2360 m in elevation; Debbie Buecher, personal communication). Further north or at higher elevations, certain bats might use snow for hibernation.

### Additional Note

On 9 December 2017, YN found an Ussurian tube-nosed bat by removing snow. He immediately covered the bat with snow. Then, on 15 April 2018 he observed a bat emerge from this precise location. The bat flew away. This suggests that the species can hibernate in the snow.

## Methods

### Fact-finding

In 2005, we initiated our study by contacting authors of available reports of bats found in snow^34–39^. We contacted observers if the authors were not involved in observation. We recorded exact location, date and time, weather conditions, the status of snow in the vicinity, the condition of each bat, and other circumstances. We also searched for additional accounts in photos and postings on the Internet, through personal contacts, and publicity of the phenomenon through public and media outreach, for which we sought the same details. In the process, one of the authors (HH) helped document two new accounts of bats found in snow^40,41^.

Most accounts described bats being found on a dent or hole. In some accounts, observers provided measures or estimates of the depth and/or diameter of these features. Otherwise, we estimated dimensions and characteristics based on photos or illustrations. All bats were found in a spherical or semi-spherical posture and we assumed a spherical diameter of 3–4 cm for estimating energy use and snowmelt.

We summarise all accounts in Supplementary Dataset S1, Supplementary KML S1 and Supplementary Note. We corrected errors (e.g., date) or supplements inadequate descriptions in the original sources.

### Meteorological analysis

We analysed the meteorological conditions associated with each account. We estimated elevation-adjusted temperatures at each site by interpolation with a lapse rate of 0.57 °C per 100 m based on data from the nearest meteorological stations^42^. Given that most bats were found at high-elevation sites while the most proximate meteorological stations were typically at much lower elevations, we used snow depth data from the nearest stations at similar elevations when available.

### Searching for bats on snow

By 2013 and with 18 collected accounts, we were convinced that the bats found in snowmelt season are buried near the bottom of snow cover and exposed by snowmelt shortly before snow disappearance. By that time, we were also convinced that *M*. *ussuriensis*, which occurs in most of the forests of Hokkaido^43^, also exhibits this behaviour in the lowland forests where we routinely study them in summer. Thus, since 2013, we focused our search on residual piles of snow in spring less than 20 cm deep, regardless of forest type or area. Because snow depth can vary considerably across a landscape, we first selected an area where bare ground was partly visible and conducted the search on foot (snow boots or mountain skis), leaving no thin-snow corners unsearched. We paid special attention to snow surfaces within about 10 m from the snow edge. To avoid overlooking bats in holes, we tried to walk with less than 5 m space in between search routes. As spring progressed, search areas naturally became higher in elevation (Supplementary Video S1, S2). We searched opportunistically: mostly on weekends or holidays, or during lunch breaks or after work on weekdays. We spent more effort on warm days (e.g., daytime high > 10 °C) because faster snowmelt increased the chance of bat exposure. We also spent more effort later in the afternoon, because we assumed the number of exposed bats accumulated towards sunset.

### Observing bats on snow

We observed some of the bats we found until after sunset. We refrained from touching the bats and used dim red lights or thermography (Thermoshot F30: NEC Avio) to minimize disturbance. With the latter, we monitored the change in the maximum body surface temperature of the bats ±2 °C or ±2%, whichever was greater.

### Assessing spring snowmelt

Loss of snow cover is a complex process involving sublimation, evaporation, melt and infiltration of meltwater, which are affected by air temperature, wind, humidity, solar radiation, and even rainfall and fog^15,44,45^. We assessed the rate of snow loss using the degree-day method, whereby the amount of run-off snowmelt water is estimated by assuming it is proportional to a daily average *T*_a_^44,45^. This is a rough estimate that ignores all other relevant factors, is often applied to large areas (typically on a watershed basis), and uses an empirical multiplication factor (called “degree-day factor”), ranging 0.3–1.0 cm/°C by area and melting stage. Because the resulting number is a water-equivalent value of depth, its division by snow density gives the reduction in depth of snow cover. Snow density increases as winter progresses; it can be as high as 0.35–0.55 g/cm^3^ at the end of snow season^44,45^. Because degree-day and snow-density factors cancel each other out, the daily average *T*_a_ in °C roughly indicates magnitude in the depth of snow cover loss in cm/day (e.g, the average daily air temperature of 10 °C predicts 10 cm reduction of snow depth during that day). The melt occurs both at the top and the bottom of snow cover, but the former contributes most.

### Simulating snowmelt under bats

We simulated snowmelt under a hypothetical bat to examine how snow holes and dents might form. We assumed snow density near the end of snow season beneath the surface was 0.35–0.55 g/cm^3^ ^44,45^. Heating energy of 334 J/g is needed to convert 0.0 °C snow to 0.0 °C water and the ratio of energy required for melting dense snow to that required for melting ice of the same mass (thermal quality) is about 0.8^44,45^. Melting a 6-cm-deep hole with a 4.5-cm diameter sphere would require 8.9–14.0 kJ of energy, equivalent to about 5–7% of bats’ body mass.

How long would a bat need to maintain a body temperature 37 °C to melt a 6-cm-deep hole? The bat’s body heat would be transferred to the snow beneath it through its furred pelage, an insulator. The “insulation value”, representing the heat flow per unit of time per unit area between two objects with a unit temperature difference^46^, of the pelage of *M*. *ussuriensis* is unknown; instead we used a surrogate value from the red bat (*Lasiurus borealis*) in summer (winter value unknown, but likely greater), measured at 0.28–0.33 °C/(kcal/h/m^2^)^47^. Similar anatomies and behaviours of *M*. *ussuriensis* and *L*. *borealis* include using foliage for roosting, having a furred uropatagium (interfemoral membrane), and curling into a spherical posture by tucking the head under the uropatagium during torpor^23,24^.

We did not account in our estimates for other variables such as thermal conductance of body tissues and other possible mechanisms to prevent heat loss, such as vasoconstriction^48,49^; hence, we made the simplified assumption that the bat body was invariably at 37 °C from core to skin despite the outflow of the heat to (thereby the cooling effect of) the snow and the predictable temperature gradient within the body. Accordingly, we kept the difference in temperature between bat skin and snow at 37 °C in our model. The reciprocal of the insulation value of *L*. *borealis* in summer is 3.03–3.57 kcal/h/m^2^/°C, which multiplied by 37 °C results in a heat flow range of 112.1–132.1 kcal/m^2^/h. We assumed that the lower half of the sphere representing a 4-cm-diameter hibernating bat contacted the snow, resulting in a contact area of 0.0025 m^2^. We calculated heat flow as 0.28–0.33 kcal/h, or 1.17–1.38 kJ/h. Dividing 8.9 kJ (the lower-bound estimate of energy required to melt a 6 cm hole in snow) by 1.38 kJ/h (the upper-bound estimate of heat flow from bat to snow) gives 6.4 hours: a highly conservative estimate of the time required for euthermic bats to sink 6 cm into snow.

### Data availability

We included all data generated or analyzed during this study in this published article and its Supplementary Information files.

## Electronic supplementary material

Supplementary Information

A bat found on 20 April 2013.

A bat found on 9 June 2013.

An atypical bat that was not lethargic.

The behaviour until the moment of flight.

Dataset 1

Dataset 2

Supplementary KML files.zip

Supplementary Slideshow S1.zip
