# Supplementary material for: Evidence for Ussurian tube-nosed bats (Murina ussuriensis) hibernating in snow
Source: Sci Rep. 2018 Aug 13;8:12047. doi: 10.1038/s41598-018-30357-1 (PMC6089880; doi:10.1038/s41598-018-30357-1)
Supplement: Supplementary file 1 — Supplementary Information [file 41598_2018_30357_MOESM1_ESM.pdf]

Supplementary Materials

Evidence for Ussurian tube-nosed bats (*Murina ussuriensis*) hibernating in snow

Hirofumi Hirakawa & Yu Nagasaka

<u>Contents</u>

Supplementary Table

S1. Summary of collected accounts of bats found near or under the surface of snow.

S2. Summary of authors' observations of 37 bats.

Supplementary Figure

S1. Change in snow depth and air temperature at four sites where bats were found.
S2. Seasonal change in the daily average air temperatures at 21 sites where bats were found.
S3. Change in the maximum body surface temperature of eight bats on the snow.
S4. Influence of rain.
S5. Two opposing hypotheses on the formation process of snow holes and dents where bats are found in spring.
S6. Safety from predators when bats are exposed.

Supplementary Note

S1. Brief summaries for each of 22 collected accounts.
S2. Remarks on authors' observations of 37 bats.
  1. Disturbance
  2. Bats that went missing during the daytime
  3. Difficulty of finding exposed bats

Legends for Supplementary files

Supplementary Video
S1 (31 sec, 6.0 MB): A bat found on 20 April 2013.
S2 (38 sec, 7.4 MB): A bat found on 9 June 2013.
S3 (61 sec, 12.2 MB): An atypical bat that was not lethargic.
S4 (22 sec, 5.2 MB): Behaviour until the moment of flight.

Supplementary Dataset
S1 (52 KB)**:** Data for 22 collected accounts.
S2 (53 KB)**:** Data for authors' observations of 37 bats.

Supplementary KML
S1 (260 KB)**:** The 3-D map representation of 21 sites from 22 collected accounts.
S2 (199 KB)**:** The 3-D map representation of 37 sites where authors found bats.

Supplementary Slideshow:
S1 (19.6 MB)**:** Slideshow of thermographic images of a bat found on 27 April 2014.

References for Supplementary Materials

Supplementary Table S1. Summary of collected accounts of bats found near or under the surface of snow.

Season	Total	Excavated from snow by human activities	Found on snow surface: Holes	Dents	Unclear	Snow state: Bare-ground visible	No bare-ground	unclear
Snow-accumulating season (~3 January)	**4**	3			1		3	1
Mid-winter (29 January)	**1**	1					1	
Snowmelt season (14 February~)	**17**		7	7	3	11	5	1
**Total**	**22**	**4**	**7**	**7**	**4**	**11**	**9**	**2**
Note 1.	Note 2.			Note 3.	Note 4.		Note 5.	

Notes:

1) Accounts in snow-accumulating season were from the early stage of the season (< 2 weeks from the first snow); those in snowmelt season were from near the end of the season (see Supplementary Fig. S1, S2).
2) One account in snowmelt season involved three bats; all the others involved single individuals.
3) We subjectively categorized holes and dents based on available illustrations or photos (Fig. 2d-f).
4) One in snow-accumulating season was recorded after the first substantial snow had mostly melted (see Yubari account in Supplementary Fig. S1).
5) Of five accounts in snowmelt season with no bare-ground reported, two were apprantly shortly before the dissapperrance of snow (see Supplementary Fig. S1).

For data details, see Supplementary Dataset S1.
For 3-D map representation of the sites, see Supplementary KML S1.
For summry of each account, see Supplementary Note.

Supplementary Table S2. Summary of authors' observations of 37 bats.

		Observation	Observation following encounter			
Time bats found	**Total**	imcomplete	Disappeared during daytime	Turned out to be dead	Flight observed after sunset	Flight not observed for 3 hours after sunset
Before sunset						
7-8 hrs	**1**		1			
6-7 hrs	**2**	1			1	
5-6 hrs	**2**	1			1	
4-5 hrs						
3-4 hrs	**3**		1		2	
2-3 hrs	**4**				4	
1-2 hrs	**8**	4			4	
0-1 hrs	**12**	2		1	8	1
After sunset						
0-1 hrs	**5**	3			2	
**Total**	**37**	**11**	**2**	**1**	**22**	**1**
			Note 1.		Note 2–4.	Note 5.

Notes:
1) For details, refer to Supplementary Note and Supplementary Video S3.
2) All the observed flights were within 94 minutes after sunset (Fig. 3a).
3) We observed eight of these bats with thermography (Fig. 3; Supplementary Fig. S3; Supplementary Slideshow S1).
4) Four of these bats were wet by rain (Fig. 3a; Supplementary Fig. S4).
5) We did not find this bat there next morning.
For data details see Supplementary Dataset S2.
For 3-D map representation of the sites, see Supplementary KML S2.

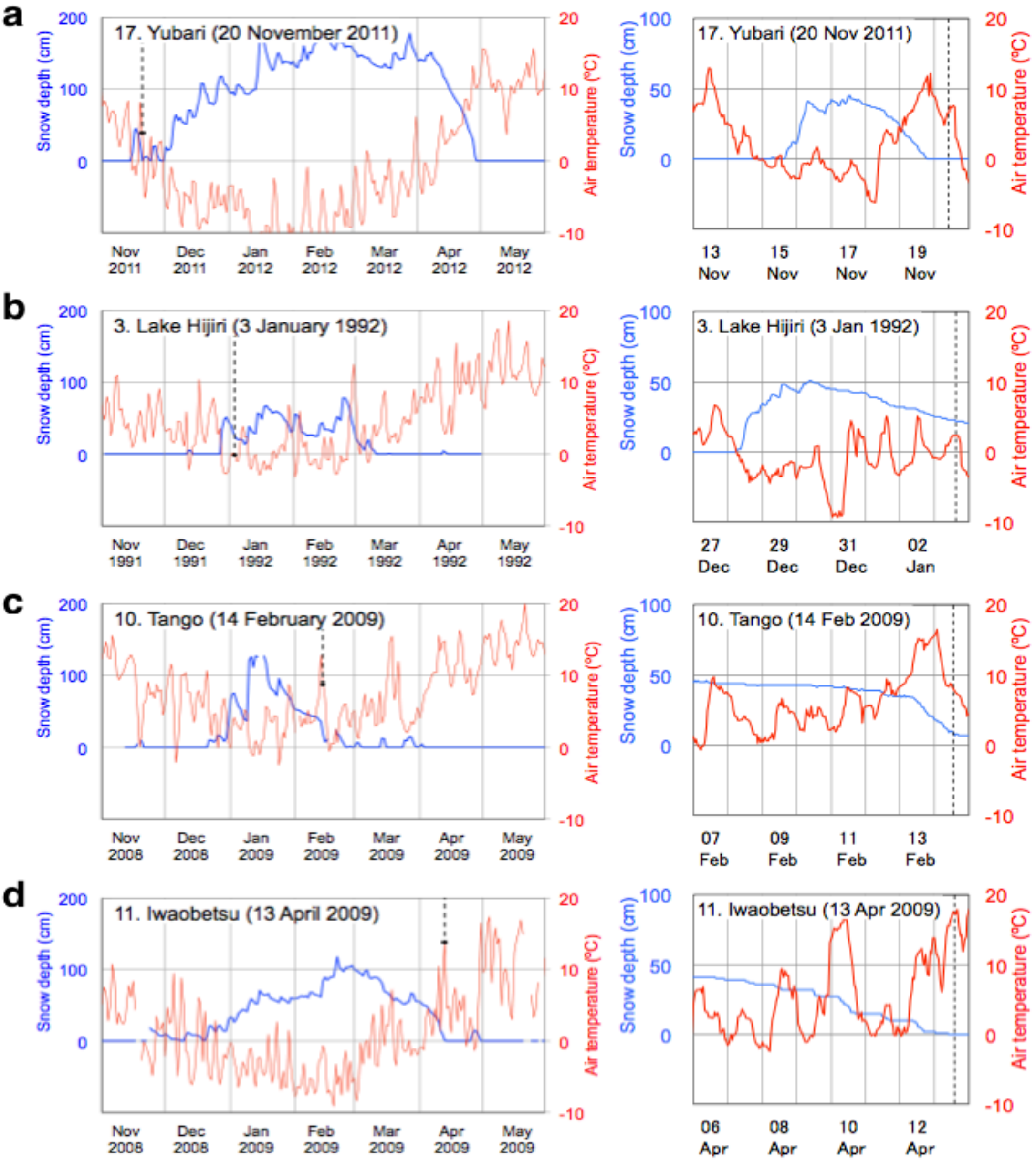

Supplementary Figure S1. Change in snow depth (SD) and air temperature (AT) at four sites where bats were found. The left graphs show the daily changes for seven months; the right shows hourly changes for eight days prior to each bat being found. The vertical dashed lines indicate the day or time when bats were found; the dots on each vertical dashed line indicate the average temperature of the day. These data came from nearby weather stations (WS): AT was adjusted 0.57 ºC per 100 m for elevational differences. (**a**) WS (for SD) is 2.5 km North, +5 m in elevation from the bat location; WS (for AT): 29 km NNE, -385 m. (**b**) WS (SD): 58 km WSW, -10 m; WS (AT): 13 km NW, -527 m. (**c**) WS (SD and AT): 6 km NW, -12 m. (**d**) WS (SD and AT): 13 km SW, -6 m. Applicable snow data were not available for the other 17 sites where bats were found, but temperature data are shown in Supplementary Fig. S2.

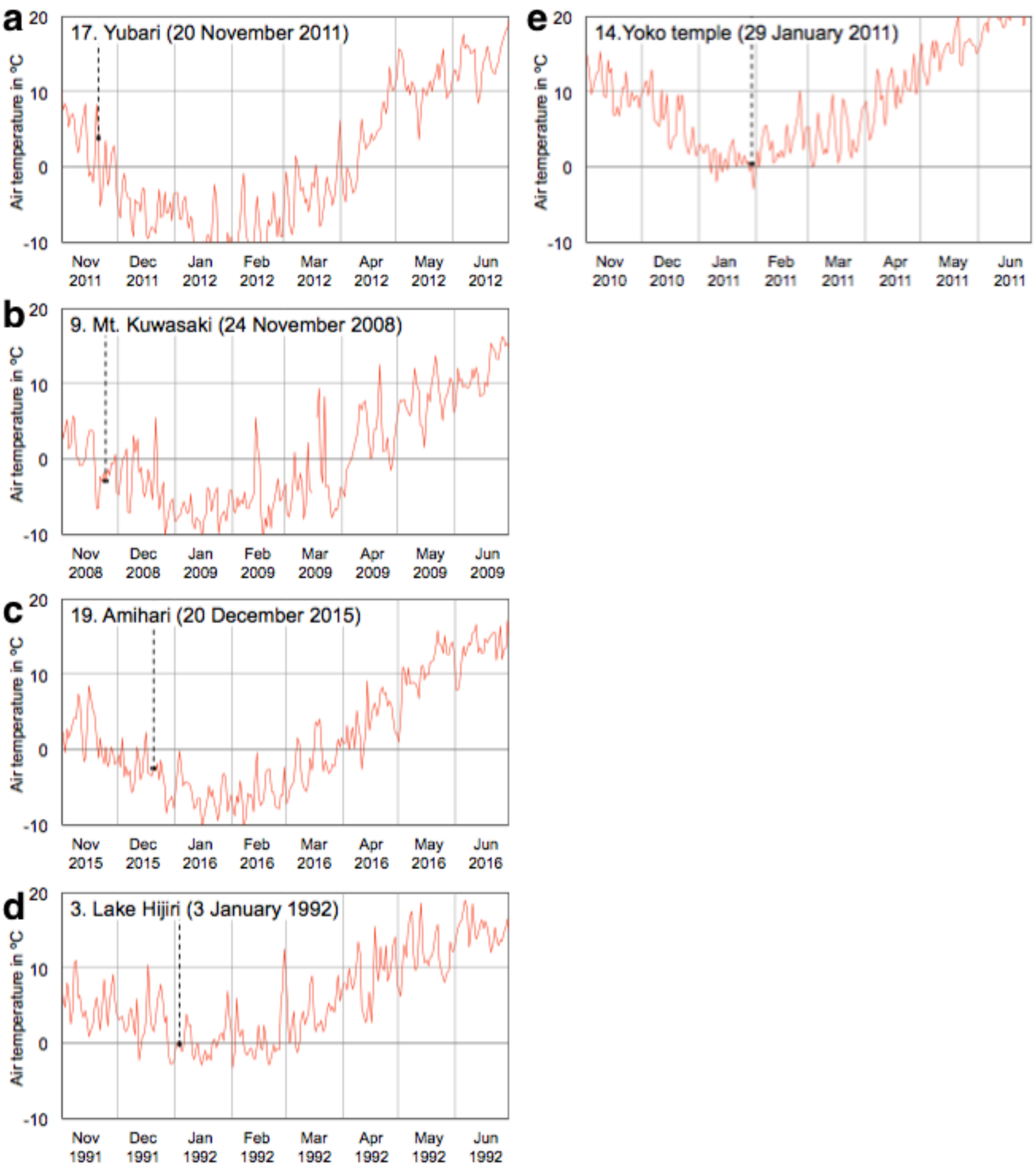

Supplementary Figure S2-1. Seasonal change in the daily average air temperatures at five sites where bats were found in early (a–d) or mid- (e) winter. These data came from nearby weather stations and were adjusted 0.57 ºC per 100 m for elevational differences. The vertical dashed lines indicate the day bats were found; the dot on each vertical dashed line indicates the average temperature that day.

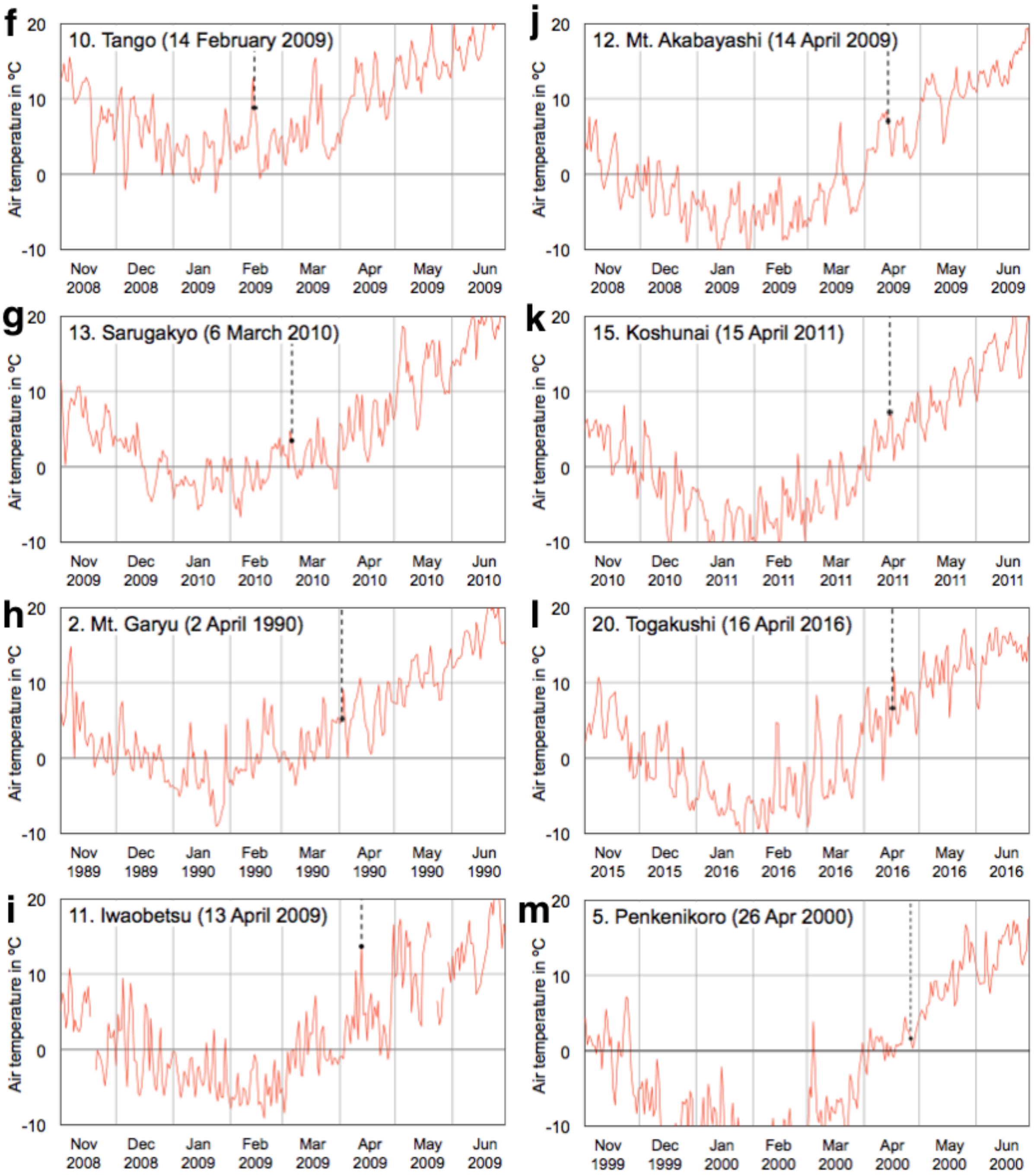

Supplementary Figure S2-2. Seasonal change in daily average air temperatures at eight sites where bats were found in February (f), March (g) and April (h–m). These data came from nearby weather stations and were adjusted 0.57 °C per 100 m for elevational differences. The vertical dashed lines indicate the day bats were found; the dot on each vertical dashed line indicates the average temperature that day.

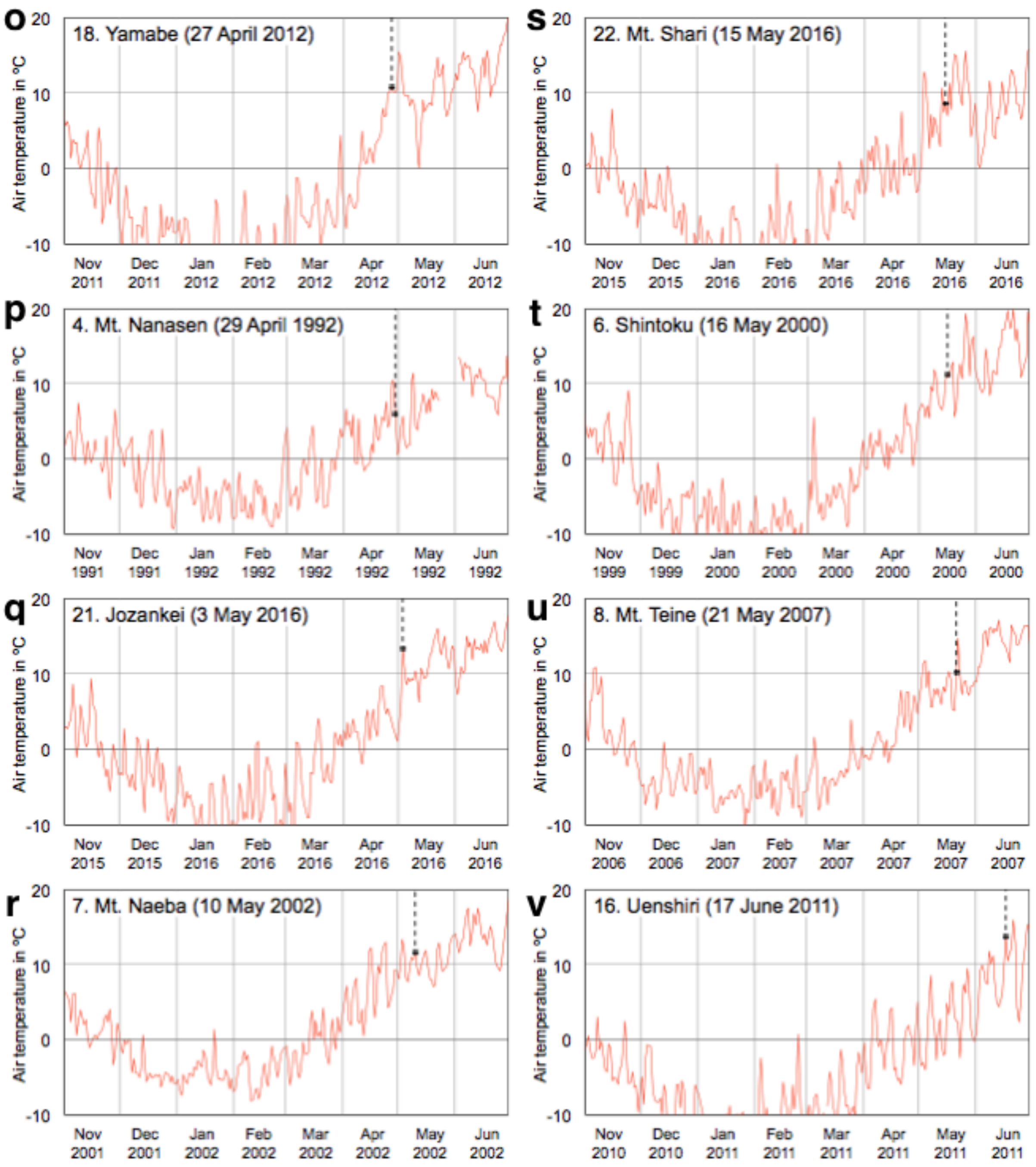

Supplementary Figure S2-3. Seasonal change in daily average air temperatures at eight sites where bats were found in April (o–p), May (q–u), and June (v). These data came from nearby weather stations and were adjusted 0.57 ºC per 100 m for altitudinal differences. The vertical dashed lines indicate the day bats were found; the dot on each vertical dashed line indicates the average temperature that day.

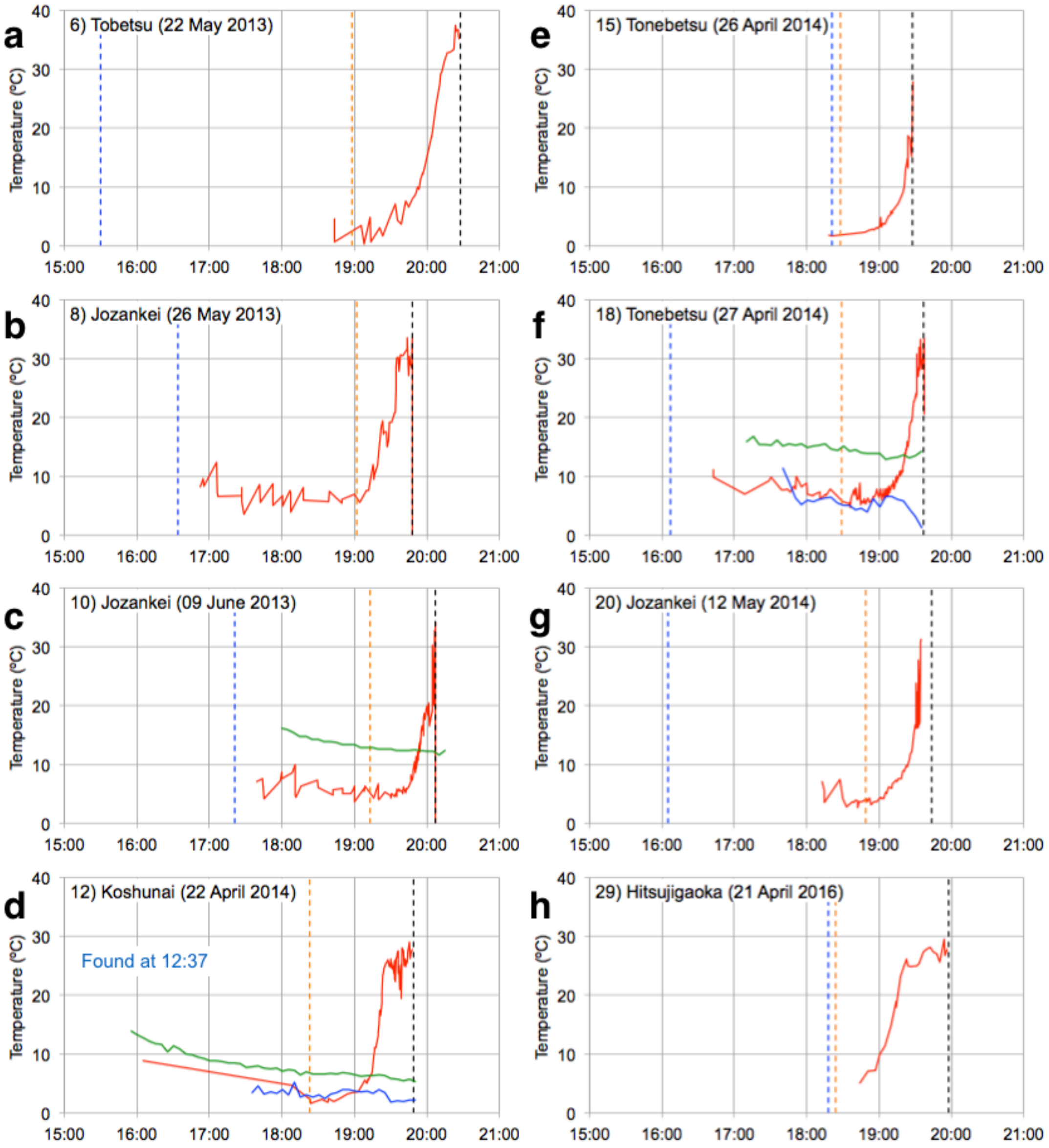

Supplementary Figure S3. Change in the maximum body surface temperature of eight bats on the snow. (**a–h**) The maximum body surface temperatures (red lines) were measured by thermography; the camera was manually triggered at irregular intervals. The vertical dashed lines indicate the time a bat was found (blue), sunset that day (orange), and when the bat flew off (black). Movements of the bats caused change in temperature measures as different parts of the body moved through the measurement area of the thermal image sensor. Measurements of bat surface temperatures were usually highest when hairless areas around the eyes and innermost parts of the ears were in line of site of the thermal camera (Fig. 3b; Supplementary Slideshow S1). Due to camera battery failure, measurement for the bat depicted in panel **g** ended before the bat flew off. Air temperatures depicted at a few cm and 150 cm above the snow surface (blue and green lines, respectively, in panels **c**, **d**, and **f**) were measured using thermocouple thermometers.

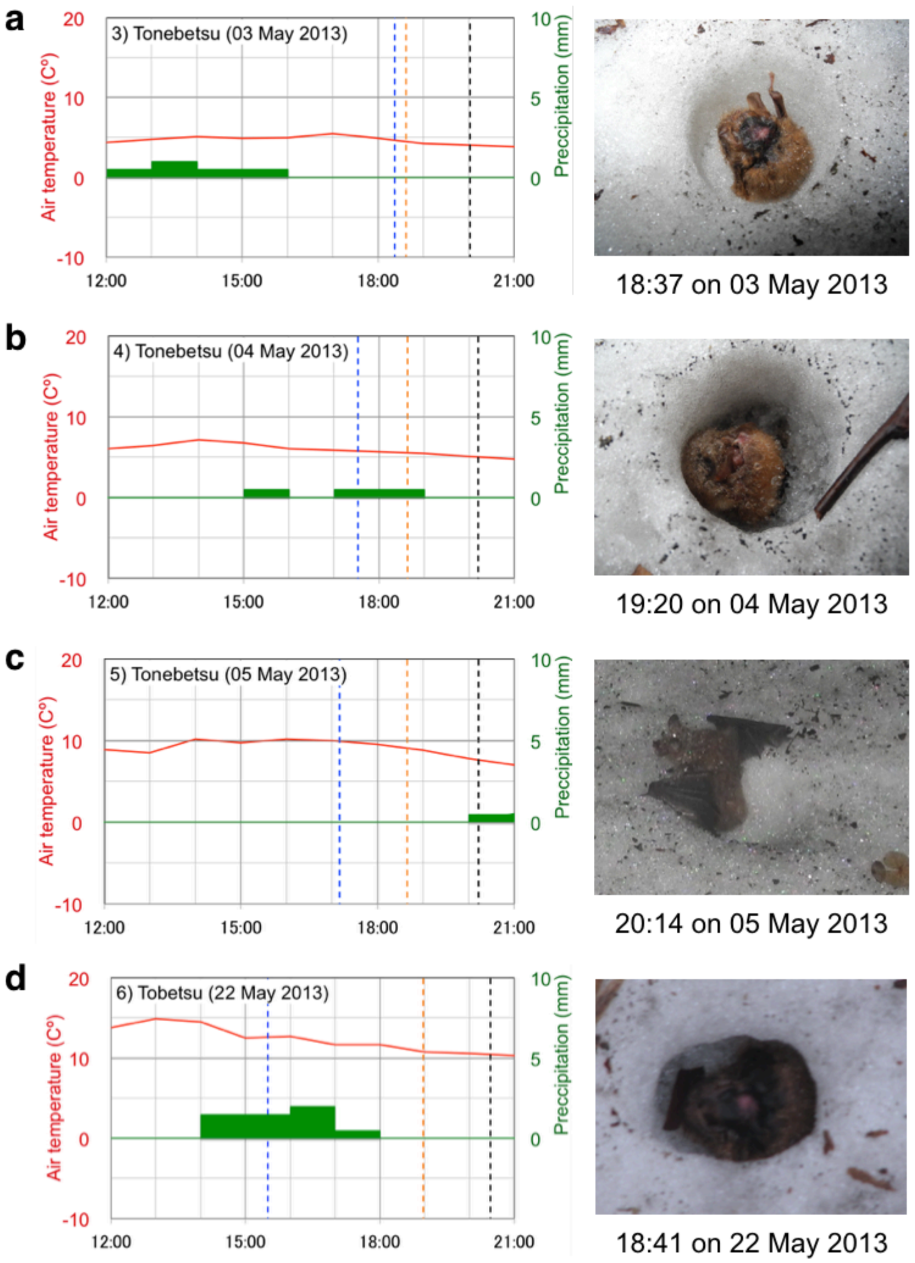

Supplementary Figure S4. Influence of rain. (**a-d**) These bats were wet by rain, but managed to take flight after sunset. The green bars in the graphs represent mm of precipitation per hour at the nearest weather stations; the red lines show hourly air temperature measured at the nearest weather station, adjusted 0.57ºC per 100 m elevation. The nearest weather station for **a**, **b**, and **c** was located 3.3–3.7 km north of the sites; the station for **d** was located 6.7 km south. The vertical dashed lines indicate the time of day that bats were found (blue), sunset that day (orange), and when the bat flew off (black). For homeothermic mammals, such conditions would cause hypothermia and could be lethal, but for these bats being wet in the snow did not seem to be a serious problem, although it may have extended the time it took them to warm sufficiently to take flight (Fig. 3a).

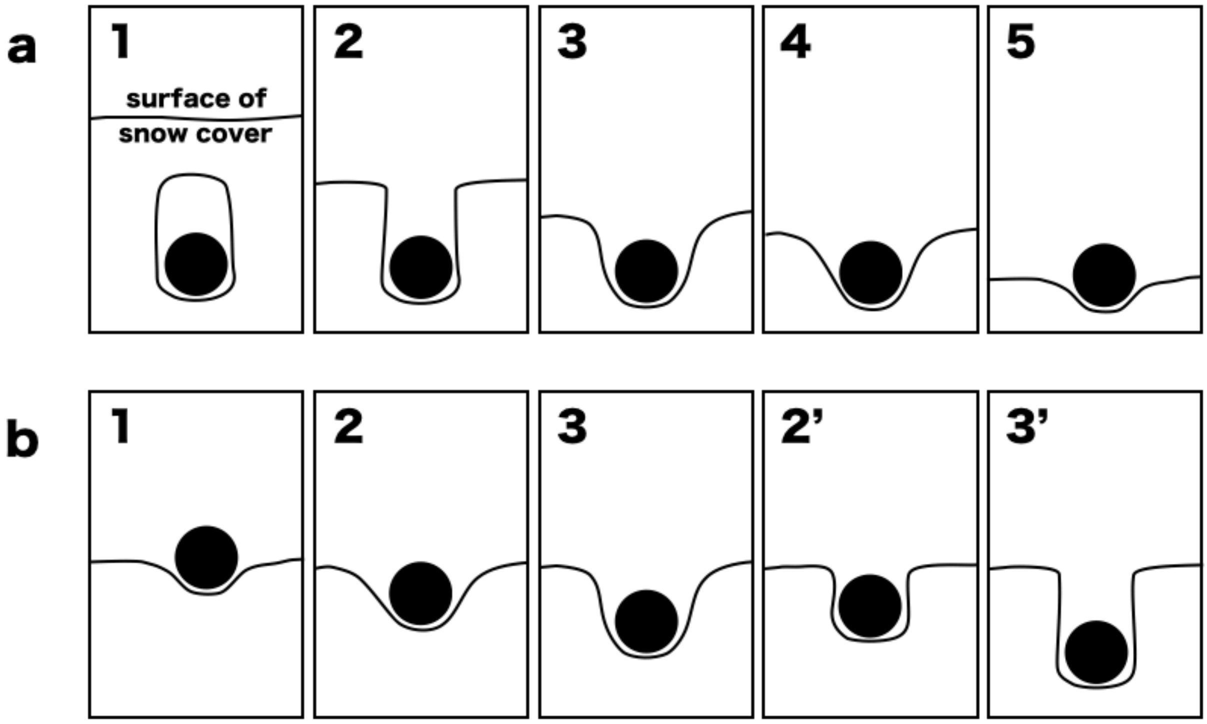

Supplementary Figure S5. Two opposing hypotheses on the formation process of snow holes and dents where bats are found. (**a**) Cavity-exposure hypothesis, in which vertical cylindrical cavities are preformed inside snow cover (1), exposed as the snow surface lowers during spring snowmelt (2), and then become dents (3–5). Progression of the process depends on air temperatures and other meteorological conditions. (**b**) Sit-and-sink hypothesis, in which bats that settle on snow (1) subsequently form dents (2–3) or holes (2'–3') as snow melts under and around them. For this process to occur, snow under and around the bats must melt faster than the open snow surface surrounding the bats.

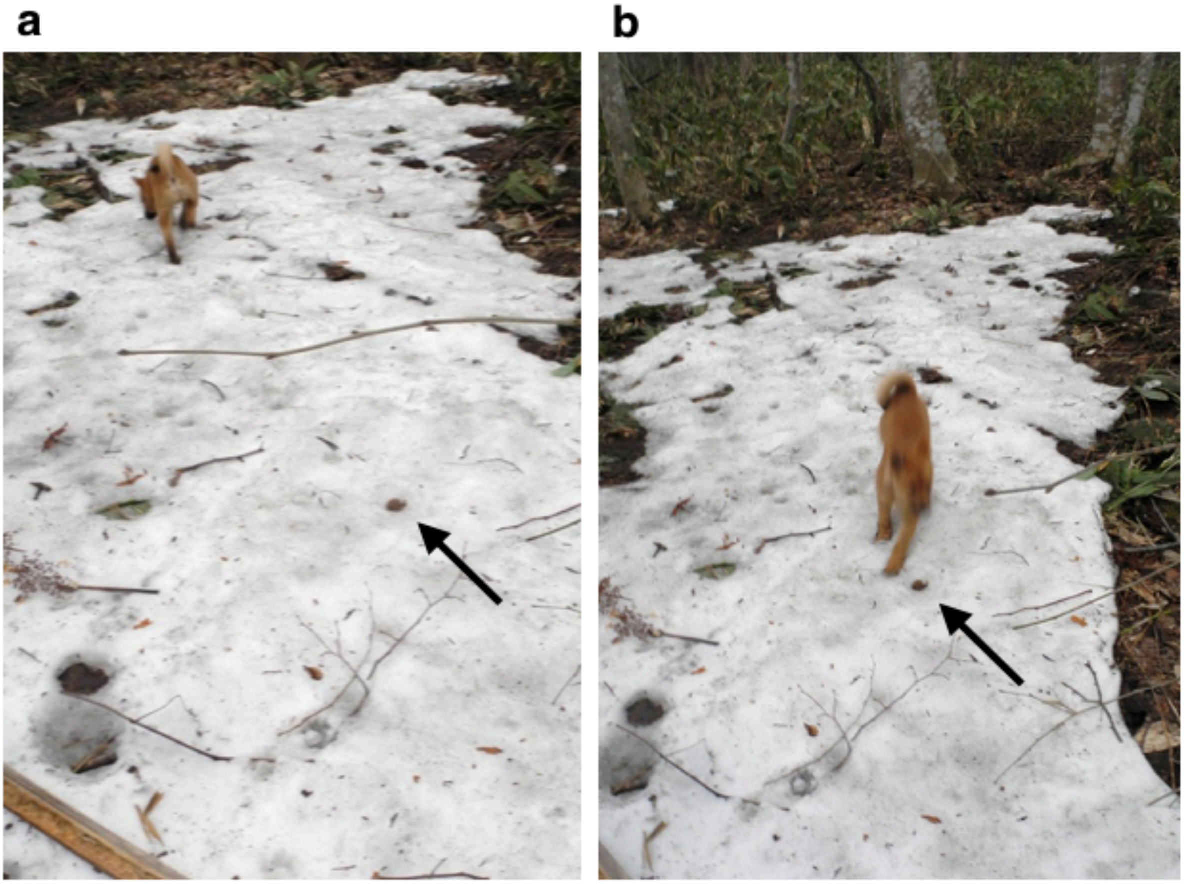

Supplementary Figure S6. Safety from predators when exposed. (**a-b**) Bats in snow seem most vulnerable to predation when exposed at the final stage of hibernation. One of the authors (YN) took his dog, Yuki ♂, in search of bats on 14 April 2015. Upon finding one at 17:25, he released Yuki to see his response. The dog walked past within about 1 m of the bat without noticing it (**a**); when returning at a run, the dog nearly stepped on the bat, still without noticing it (**b**). This implies that these bats have no distinguishing odours that attract the attention of carnivores with an acute sense of smell. A similar observation was reported for a pet gray fox (*Urocyon cinereoargenteus*) and a species of *Lasiurus* during warmer seasons in Georgia, USA [51]. Bats in snow are not easy to visually detect. Snow surfaces at this season are blotted with dirt, twigs, and leaves because most debris and detritus that fell on (and were buried in) the snow during winter are exposed. After presumably being buried early in the snow season, bats are among the last objects to be exposed (Fig. 5a). *Murina ussuriensis* in a spherical posture also resembles acorns in size, shape, and colour. These features and the timing of exposure, in combination with being motionless, seem to effectively work to conceal them from predators.

Supplementary Note

S1: Brief summaries for each of 22 collected accounts.

See also Supplementary Dataset S1 for data details and Supplementary KML S1 for 3-D map representation of the 22 sites.

1. Sapporo (29 April 1964)

Found on snow cover in the vicinity of Sapporo. No other details are available. The female bat was kept in captivity for 26 days. Body mass was reported at 4 g (the timing of the measurement is unknown); other measurements in the document[24] tell that mass was likely to 0.5-g precision.

2. Mt. Garyu (2 April 1990)

Three bats were found, each in a separate snow hole. Snow cover remained only in patches on a northern slope. A group of about 15 people was taking an outdoor training; one of them noticed a hole in a patch of snow and then found a brown lump in the bottom. After being dug out and rolled on the person's palm, the object started to move and unfold wings, revealing that it was a bat. Further search in the vicinity resulted in two other bats being found. They were also dug out. While being watched, they became fully active and flew away. The document[25] included an illustration of a vertical hole that was 25 cm deep, but according to one of the observers, Mr. Hiroshi Sugishima, this was only assessed later by recollection and could have been much shallower; we disregarded the given depth in our analysis as unreliable.

3. Lake Hijiri (3 January 1992)

Found in a snow hole on a paved road near a remote lake while the person who found it was on foot. The depth of the snow on the road was around 20 cm. The snow hole was found in a rut, where the snow depth was probably around 10 cm. The traffic was scarce and one or at most a few cars likely made the rut. The hole depth to the bat from the rim was about 3 cm. The bat was in a semi-spherical posture with its abdomen upwards. Snow had started to accumulate six days earlier (Extended Data Fig. 1b). It is of note that a hole of that depth had already been formed by that time. This female bat was subsequently kept in captivity. After death, it was prepared as a museum specimen and accessioned into the Geihoku Kogen no Shizenkan (Natural History Museum of Geihoku).

4. Mt. Nanasen (29 April 1992)

Found in a snow hole in a spherical posture with its head upwards. Snow covered most of the ground in the vicinity. The person who found it was descending a trail on a mountain

ridge. Being picked up and warmed up by hand, the bat gradually aroused from torpor. The bat was taken to Professor Mizuko Yoshiyuki at Tokyo University of Agriculture, a bat biologist, one or two days later, where it was determined to be a female and at which time its body mass was 3.8 g. It was still alive at that time, although it had not been fed.

5. Penkenikoro (26 April 2000)

Found in a snow hole. The person who found it was heading for a raptor survey point. Snow totally covered the ground in the vicinity. The hole was just deep enough for the spherical bat to be below the rim. The diameter of the hole rim was measured at 4 cm, with a small gap between the bat's body and the wall. A few days later, the bat was taken to Professor Hisashi Yanagawa at Obihiro University of Agriculture and Veterinary Medicine, a wildlife biologist, where it was determined to be male and when its body mass was 5.0 g. The precision is unknown. It was probably dead then. The date given as 27 April 1999 in the document[28] by H. Yanagawa was incorrect.

6. Shintoku (16 May 2000)

Found in a snow dent in a semi-spherical posture with the abdomen downwards. This location was in a plantation of Sachalin fir (*Abies sachalinensis*). Snow covered most of the ground in the vicinity. The person who found the bat was on their way back from a field survey and kept the male bat in captivity overnight and then released it the next day where it was found.

7. Mt. Naeba (5 May 2002)

When found, this bat's body was mostly exposed on the snow surface. The person who found it was engaged in a vegetation survey. The bat was in a semi-spherical posture with its abdomen downwards. When picked up, it reacted a little. The depth of the snow at the site was approximately 50 cm. However, the snow was rapidly melting away and the visible ground surface was noticeably expanding that day.

8. Mt. Teine (21 May 2007)

Found first time on a deliberate search. The observer was Hiroaki Nakajima, a photographer who had been informed of this phenomenon by one of the authors (HH). Precise details were documented. The bat was in a hole on a mass of lingering snowpack. The diameter of the hole was about 5 cm. The depth from the bat to the rim above was around 2 cm. Depth of snow at the site was about 20 cm. When found, the bat was motionless in a spherical posture, but after an hour, it started to respond to the sounds made by Mr. Nakajima. When he slightly touched the bat, it opened its mouth, but the eyes were kept closed. Returning after a half-hour absence, Mr. Nakajima found that the bat had changed its position,

but was motionless. When he left the site three hours after finding the bat, it was still motionless.

9. Mt. Kuwasaki (24 November 2008)

Found while a group of climbers were ascending a mountain by crawling through fresh deep snow in single file. The bat was found at the foot of one of them. The depth of snow at the site was greater than 1 m, and the bat was likely at a depth within 80 cm of the snow surface. When picked up, the bat squeaked, but otherwise was motionless in a semi-spherical posture. Inspection found no obvious injuries. It was left on the undisturbed snow surface next to where it was found, still showing no signs of movement.

10. Tango (14 February 2009)

Found in a snow hole in torpor in a spherical posture (Fig. 2ac) with the abdomen downwards. This site was on an unpaved logging road, and the snow depth was likely about 50 cm. The person picked up the bat and placed it in a depression on a nearby slope with no snow cover. Two hours later, the observer returned to the site and did not find the bat.

11. Iwaobetsu (13 April 2009)

Found in a snow dent with the abdomen downwards. The bat was breathing and half aroused from torpor. When the observer picked it up, the bat gave off a defensive hiss. He placed the bat back in the dent. The snow mostly covered the ground around the site, and the depth of the snow on the spot was likely about 30–40 cm.

12. Mt. Akabayashi (14 April 2009)

Found in a dent in a spherical posture with the abdomen downwards (Fig. 2e) on a small mass of lingering snowpack. While being photographed on the observer’s palm (Fig. 2b), it gradually aroused from torpor. Before the bat became fully active, it was placed in a cavity of a tree nearby.

13. Sarugakyo (6 March 2010)

Found in a snow dent in a semi-spherical posture with the abdomen upwards. The ground was visible in patches. The depth of snow cover was 20–30 cm. When picked up, it slightly moved its nose and ears. It was soon placed back in the dent. The person who found it returned to the site the next morning, and did not find the bat.

14. Yoko temple (29 January 2011)

Found inside snow in the precinct of a temple. Snow depth was about 80–100 cm and the bat was found when the upper half of the snow cover was removed with a squared shovel. The bat was motionless in a spherical posture, and was brought inside a house. After a while it gave off a short sound and started to fly around in the room. It was then captured and

released outside.

15. Koshunai (15 April 2011)

Found in a decayed hole on a small patch of remnant snowpack in a spherical posture with the head upward. It was in a young birch forest thick with an understory of dwarf bamboos. Snow cover remained in patches. The observer tried to pick the bat up, but he stopped the attempt halfway because the bat apparently resisted it. The observer returned to the site the next day, but there was neither bat nor snow.

16. Mt. Uenshiri (17 June 2011)

Found on a small mass of remnant snowpack on a trail of a treeless mountain ridge. Bat was in a cylindrical hole with its back upwards. The bat's back was almost level with the rim of the hole. The observer breathed on the bat, but it did not move. As the bat was motionless for another 20 minutes, the observer lightly touched it, but it did not react. Presuming that it was dead, the observer picked it up, at which point the bat urinated. The bat was left on a dwarf bamboo leaf beside the trail.

17. Yubari (20 November 2011)

Found after the first snow cover of the season had almost melted away (Extended Data Fig. 1a). It was in a larch forest behind the observer's home. He took the bat home. The bat was in a semi-spherical posture and still motionless. Then, warmed by the room air, the bat started to move slowly, although its eyes remained closed. The observer took it outside and left it among woodpiles. When he checked later in the evening, he did not find the bat.

18. Yamabe (27 April 2012)

Found in a snow hole in a spherical posture with the head upward. The top of the bat's head was level with the rim of the hole. The ground was visible in the vicinity. The snow depth was 10–20 cm. Wondering what the object was, the observer touched it with the tip of a pen; the bat raised its head and made a groaning sound. The observer then left it as it was.

19. Amihari (20 December 2015)

Found in a pristine beech forest. A total of 33 people were snowshoeing in four separate groups when one of them heard a squeaking sound and found a bat on the snow surface, apparently disturbed by the others. The bat was mostly buried with only its face visible. After being scooped up with the snow, it gave off a whooshing—apparently intimidating—sound. It was placed in a cavity of a tree nearby.

20. Togakushi (16 April 2016)

Found in the bottom of a cylindrical 6–7-cm-deep hole on a remnant patch of snow in a larch forest. The observers deliberately searched for and found this bat using procedures

recommended by the authors. They watched the bat until 131 minutes after sunset using an LED light intermittently, but the bat did not fly away. This lack of flight after sunset is an anomaly compared with our other observations (see Fig. 3a). The bat might have been moribund or intimidated by the light or sound of a bear bell they kept ringing while waiting.

21. Jozankei (3 May 2016)

Found on a remnant mass of snowpack in a broad-leaved forest while the observers were searching for edible wild plants. They noticed the quivering ears and furry back of a small animal slightly protruding from the snow surface. They turned the body with a twig and realized that it was bat. The bat opened its eyes, but did not show any other movement. They set the bat to the previous position and left.

22. Mt. Shari (15 May 2016)

Found motionless in a snow dent with its abdomen upward in a mixed forest while the observer was searching for sable faeces. Having some knowledge about this phenomenon, the observer did not touch the bat. The ground was only partly covered with snow.

Supplementary Note

S2: Remarks on authors' observations of 37 bats.

See also Supplementary Dataset S2 for data details and Supplementary KML S2 for 3-D map representation of the sites where 37 bats were found.

1. Disturbance

We tried to minimize disturbance to bats during observation: one of the authors (HH) only used a thermography camera for after-dark observation; the other author (YN) intermittently used dim red light and occasionally also took flash photographs (with visible light). The degrees to which the bats perceived possible stimuli arising from observer presence and how much such perception affected their behaviour were unknown. In some cases, however, the bats apparently responded with an instantaneous jerk to the sounds or movements that we made, indicating the bats were able to perceive to those stimuli despite their lethargic state.

The following two cases are of note regarding disturbance.

1-1. Suspected strong stimuli: A bat found on 12 May 2014

An animal photographer found this bat and one of the authors (HH) joined him for observation with thermography. The photographer’s camera flash and sounds were so frequent (sometimes almost continuous) that it was difficult to assume the bat was unaffected, although the influence was not apparent in the data (Supplementary Figure S3g).

1-2. Delay of flight: A bat found on 12 April 2015

After finding a bat at 60 minutes before sunset (18:11), one of the authors (YN) started observation 67 minutes after sunset. In trying to take a video, he kept illuminating the bat with red light. In the process, he once dropped the light close to the bat, possibly creating a strong stimulus. Rain began to fall at about 20:30. He turned the red light off at 20:55 and as the bat still did not move, he gave up observation at 21:15, 184 minutes after sunset. The next morning, shortly after 6:00, he did not find the bat on the spot. The weather had cleared by then.

When the rain started 139 minutes after sunset, the bat's flight was already much delayed compared to other observations. Although the added thermal burden of metabolically warming with wet fur may have caused the delay, we cannot rule out the possibility that near-continuous exposure to the light or the strong stimulus of the dropped light may have caused the delay.

2. Bats that went missing during the daytime

Most of the bats we found during the daytime stayed where they were found until after sunset (Supplementary Table S1; Supplementary Dataset S2). However, in the following two cases, the bats went missing during the daytime.

2-1. Snow disappearance: A bat found on 27 April 2014

The bat was found at 11:05 on a thin cover of snow (less than 4 cm in thickness). The air temperature was higher than 20ºC and the snow was rapidly melting away. A video recorded while we were away showed that underlying bamboo grass stood up as the snow beneath and around the bat disappeared, and the bat (either deliberately or accidentally) dropped to the ground at 12:52. Later, we searched around the spot, but we could not find the bat.

2-2. A bat that was not lethargic: A bat found on 15 April 2017

This bat was motionless when found at 14:42, but soon after it became very active (Supplementary Video S3). We left the spot when it became motionless again, 20 minutes after first being found. When we returned 45 minutes later, the bat was not there.

The following case is also of note regarding snow disappearance during the daytime.

2-3. A bat that stayed lethargic even after disappearance of snow: A bat found on 21 April 2016

The bat was found at 12:28 on a thin cover of snow (less than 4 cm in thickness). The snow underneath the bat had totally disappeared by 17:33, but the bat remained motionless on the ground until the observation stopped at 17:40, 44 minutes before sunset.

3. Difficulty of finding exposed bats

Looking at the amount of search efforts for finding 37 bats (Supplementary Dataset S2), one might think the phenomenon must be very rare. However, the low success rate does not necessarily mean the scarcity of buried bats. Search efficiency varies depending primarily on air temperatures and snowmelt conditions and can be remarkably high in good conditions. For example, we found six bats on two consecutive days in an area in 2014. The maximum temperatures were above 20ºC on both days and snow was quickly disappearing from the area. However, restricting search efforts to good conditions will reduce search opportunities. Thus, there is a dilemma between search efficiency and effort. More efforts may bring more encounters with bats, but inevitably lead to lower search efficiency.

Similarly, more search hours on a day can only lead to a lower search efficiency because there is always a chance of bat exposure after the search conducted before sunset. In order to find all the buried bats in an area, we would need to scan the whole surface of the snow during sunset every day while snow is disappearing from the area. Still, we cannot avoid overlooking some bats that are exposed and take flight during the night.

Legends for Supplementary Files

Supplementary Video

S1 (31 sec, 6.0 MB)**: A bat found on 20 April 2013.**

This video and Video S2 are provided to show differential conditions between two cases of bats being found. The snow cover was disappearing much earlier at this site (150 m in elevation) than at the site in Video S2 (750 m in elevation). This site was located in a broad-leaved forest, where trees had not yet foliated when the bat was found. The estimated average temperature for one week prior to this observation was 4.9ºC. Short bamboo grass covered the forest floor.

S2 (38 sec, 7.4 MB)**: A bat found on 9 June 2013.**

This bat was found 50 days later and 600 m higher in elevation than, and 27 km southwest of, that in Video S1. Although the snow cover status is similar, the broad-leaved trees had already been foliated, active singing of birds was heard and insects were flying around, indicating that the spring in this video was more progressed than that in Video S1. This is because the thicker snow cover at higher elevation in general requires more heat to melt, effecting the snow remain until it gets warmer. The estimated average air temperature for one week prior to this observation was 11.5ºC. The floor of the mixed forest was covered with thick and tall bamboo grass, which was standing up where the snow cover disappeared. It is likely that the bat alighted on snow cover after early snowfall flatted out the vegetation.

S3 (61 sec, 12.2 MB)**: An atypical bat that was not lethargic.**

Found at 14:42 on 15 April 2017, this bat became full of motion soon after it was found. However, it did not move out of the dent and it became motionless again after a while. We left the spot 20 minutes after it was found. When we returned 45 minutes later, however, it went missing. What occurred to the bat is unknown. It might have flew off, or predators might have preyed upon it as the bat moving about on the snow could easily attract the attention of predators. It was a warm day and the estimated air temperature when it was found was 17.1ºC. However, no other bats, including those found at even higher temperatures (Supplementary Dataset S2), have been observed in such an active state. Hence, the high temperature (at least alone) cannot account for the state. Although the cause of the state is thus unclear, this observation suggests that on some conditions, bats may become active.

S4 (22 sec, 5.2 MB)**: The behaviour until the moment of flight.**

This thermographic video shows the behaviour of a bat for 18 seconds until the moment of flight. It was at 19:38, 71 minutes after sunset, on 24 April 2017. The video was taken using FLIR ONE for iOS Personal Thermal Imager. The temperatures displayed on the image were the measurements at the centre of the screen as indicated by the cross.

Supplementary Dataset

S1 (52 KB)**: Data for 22 collected accounts.**

This excel file contains data for 22 collected account.

S2 (49 KB)**: Data for authors' observations of 37 bats.**

This excel file contains data for authors' observations of 37 bats and data for search efforts.

Supplementary KML

S1 (260 KB)**: The 3-D map representation of 21 sites from 22 collected accounts.**

Open this file with Google Earth (or Google Earth Pro), which will display the file's contents. Show the sidebar and check the list of display items. For details, see the legend displayed on the map window. If the letters of the legend are not aligned, reload the file and/or resize the window (or hide the legend by checking off the legend checkbox). The site of the earliest account in Sapporo in spring 1964 is not shown due to lack of details.

S2 (199 KB)**: The 3-D map representation of 37 sites where authors found bats.**

See the instruction above for how to use the file.

Supplementary Slideshow (19.6 MB):

S1: Slideshow of thermographic images of a bat found on 27 April 2014.

This archive (zipped) file contains a set of files to display the slideshow of thermographic images

showing the change in body surface temperature and posture of a bat from 133 minutes before sunset until flight, 68 minutes after sunset. Double-clicking the Slideshow.html file in the folder will activate the default browser to display the contents. Some relevant photos, a data table, and a data graph are also displayed on different panels in the same window.

References for Supplementary Materials

51. Constantine, D. G. Ecological observations on Lasiurine bats in Georgia. *J. Mammal.* **39,** 64-70 (1958).
