# Supplementary material for: Evidence for Ussurian tube-nosed bats (Murina ussuriensis) hibernating in snow
Source: Sci Rep. 2018 Aug 13;8:12047. doi: 10.1038/s41598-018-30357-1 (PMC6089880; doi:10.1038/s41598-018-30357-1)
Supplement: Supplementary file 9 — Supplementary Slideshow S1.zip [file 41598_2018_30357_MOESM9_ESM.zip › Slideshow/Slideshow.html]

Ussurian Tube-nosed Bat on 27 April 2014


(


)
 


 


 


  **PhotoSearch Ver.1.2E (7 August 2017)**
  **Designed by H. Hirakawa**
