# Supplementary figures and images for: Evidence for Ussurian tube-nosed bats (Murina ussuriensis) hibernating in snow (part 2 of 2)
Source: Sci Rep. 2018 Aug 13;8:12047. doi: 10.1038/s41598-018-30357-1 (PMC6089880; doi:10.1038/s41598-018-30357-1)

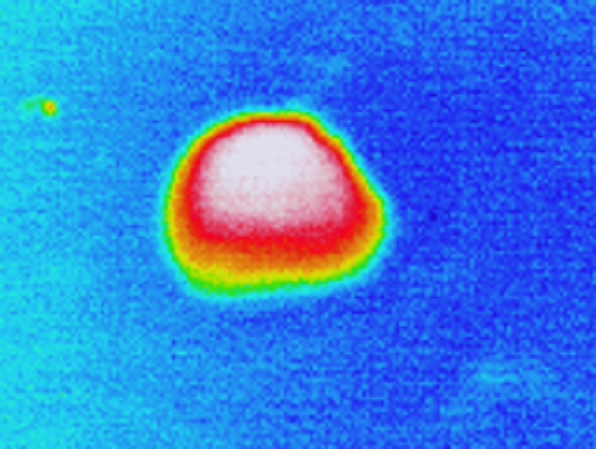

Supplement: Supplementary file 9 — Supplementary Slideshow S1.zip [file 41598_2018_30357_MOESM9_ESM.zip › Slideshow/photos/UTNB140427-002.jpg]

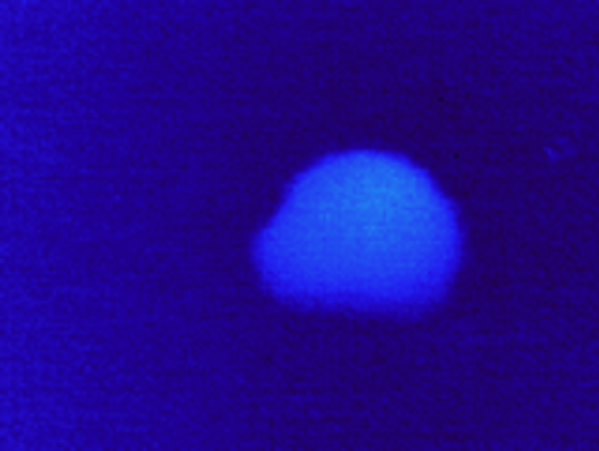

Supplement: Supplementary file 9 — Supplementary Slideshow S1.zip [file 41598_2018_30357_MOESM9_ESM.zip › Slideshow/photos/UTNB140427-016.jpg]

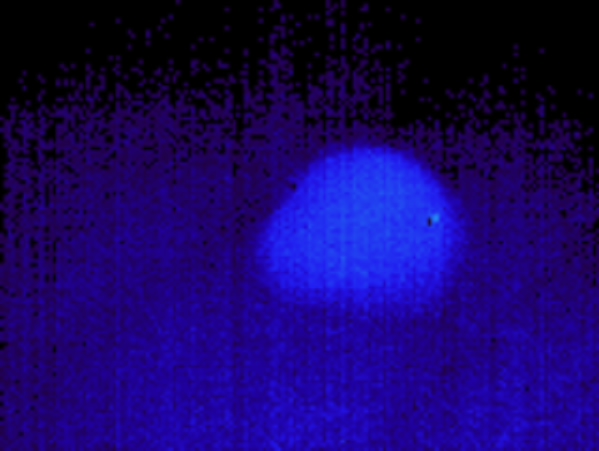

Supplement: Supplementary file 9 — Supplementary Slideshow S1.zip [file 41598_2018_30357_MOESM9_ESM.zip › Slideshow/photos/UTNB140427-017.jpg]

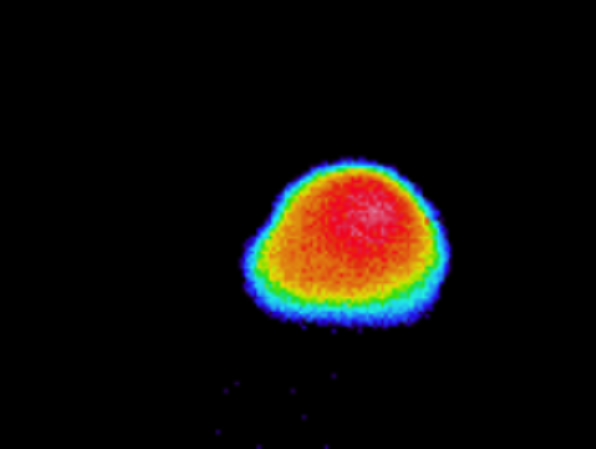

Supplement: Supplementary file 9 — Supplementary Slideshow S1.zip [file 41598_2018_30357_MOESM9_ESM.zip › Slideshow/photos/UTNB140427-003.jpg]

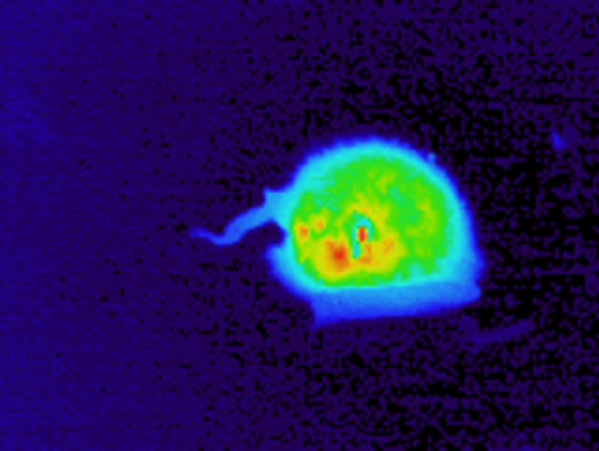

Supplement: Supplementary file 9 — Supplementary Slideshow S1.zip [file 41598_2018_30357_MOESM9_ESM.zip › Slideshow/photos/UTNB140427-163.jpg]

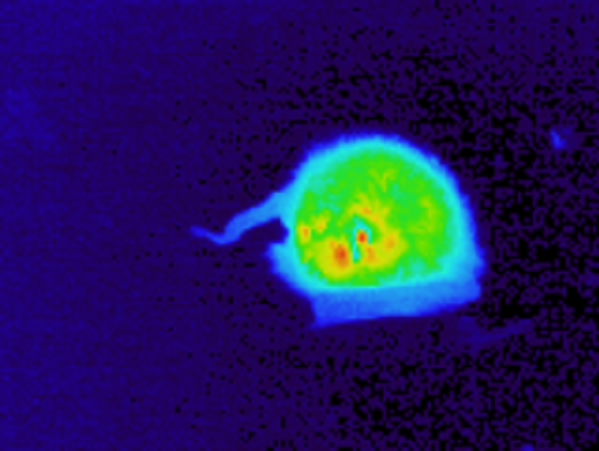

Supplement: Supplementary file 9 — Supplementary Slideshow S1.zip [file 41598_2018_30357_MOESM9_ESM.zip › Slideshow/photos/UTNB140427-161.jpg]

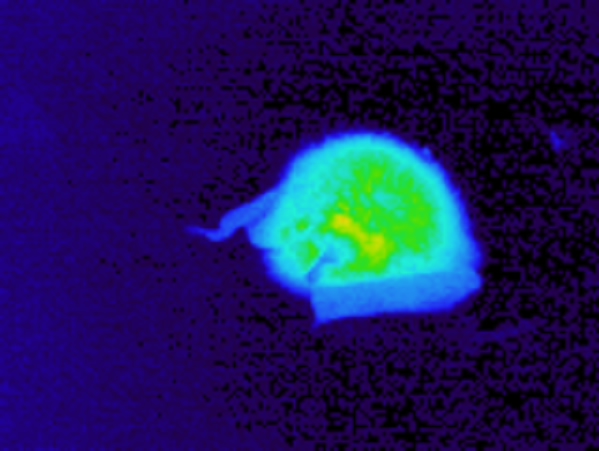

Supplement: Supplementary file 9 — Supplementary Slideshow S1.zip [file 41598_2018_30357_MOESM9_ESM.zip › Slideshow/photos/UTNB140427-149.jpg]

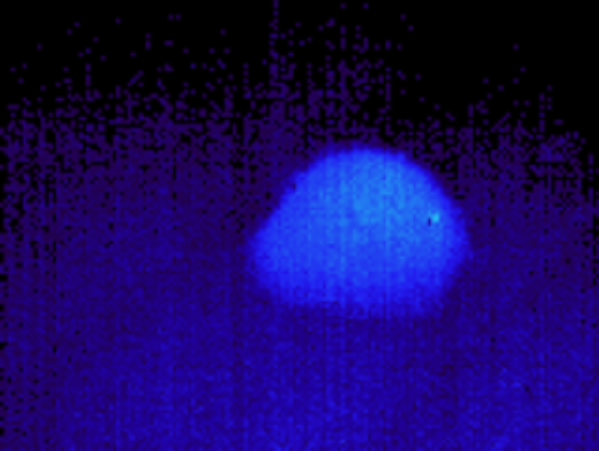

Supplement: Supplementary file 9 — Supplementary Slideshow S1.zip [file 41598_2018_30357_MOESM9_ESM.zip › Slideshow/photos/UTNB140427-015.jpg]

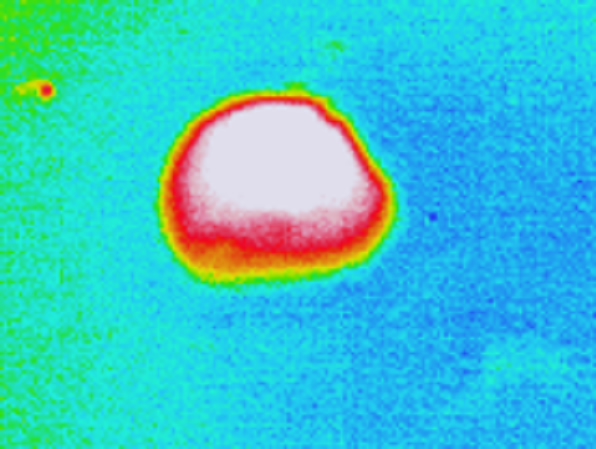

Supplement: Supplementary file 9 — Supplementary Slideshow S1.zip [file 41598_2018_30357_MOESM9_ESM.zip › Slideshow/photos/UTNB140427-001.jpg]

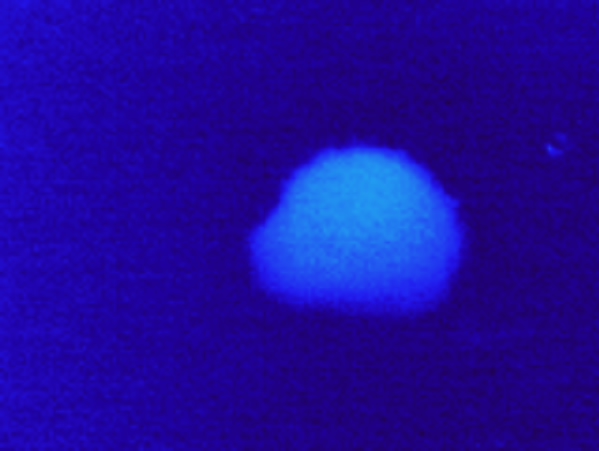

Supplement: Supplementary file 9 — Supplementary Slideshow S1.zip [file 41598_2018_30357_MOESM9_ESM.zip › Slideshow/photos/UTNB140427-014.jpg]

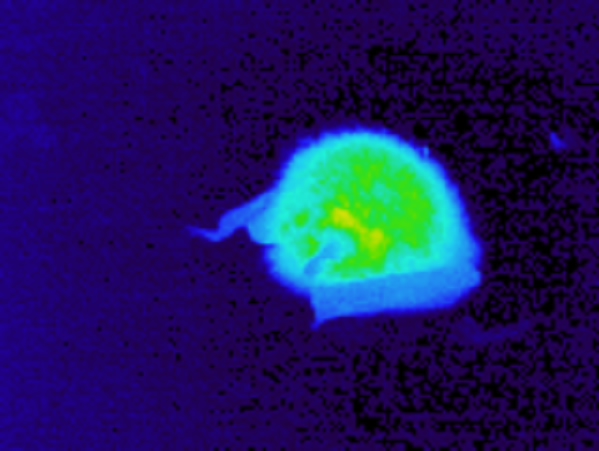

Supplement: Supplementary file 9 — Supplementary Slideshow S1.zip [file 41598_2018_30357_MOESM9_ESM.zip › Slideshow/photos/UTNB140427-148.jpg]

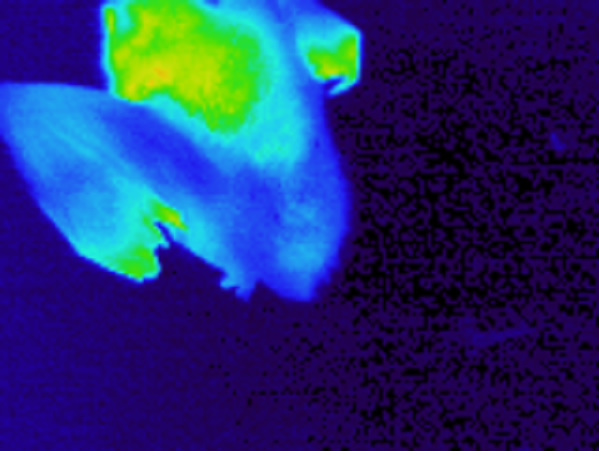

Supplement: Supplementary file 9 — Supplementary Slideshow S1.zip [file 41598_2018_30357_MOESM9_ESM.zip › Slideshow/photos/UTNB140427-174.jpg]

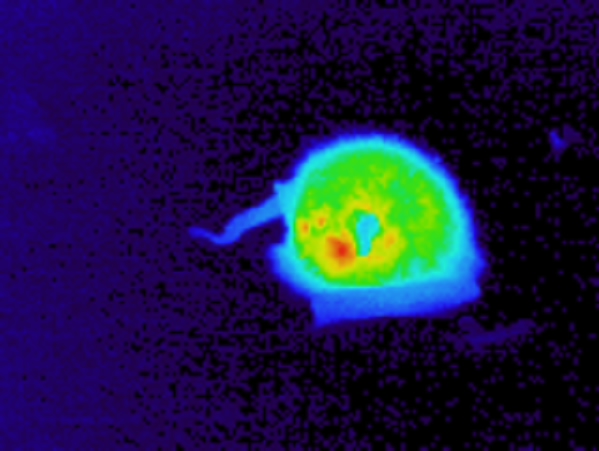

Supplement: Supplementary file 9 — Supplementary Slideshow S1.zip [file 41598_2018_30357_MOESM9_ESM.zip › Slideshow/photos/UTNB140427-160.jpg]

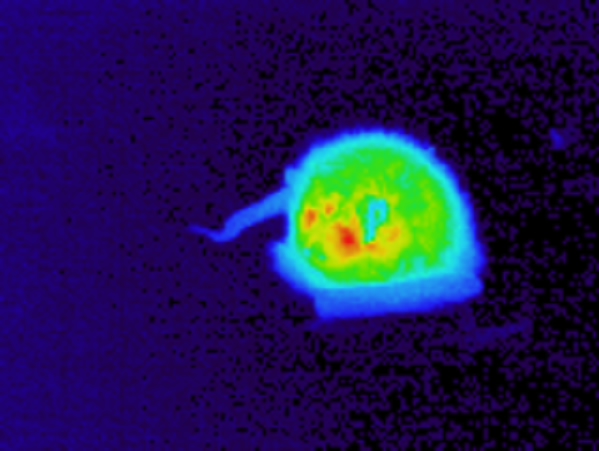

Supplement: Supplementary file 9 — Supplementary Slideshow S1.zip [file 41598_2018_30357_MOESM9_ESM.zip › Slideshow/photos/UTNB140427-158.jpg]

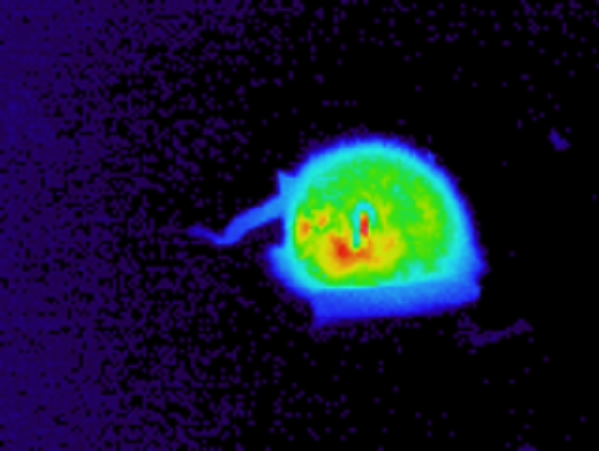

Supplement: Supplementary file 9 — Supplementary Slideshow S1.zip [file 41598_2018_30357_MOESM9_ESM.zip › Slideshow/photos/UTNB140427-164.jpg]

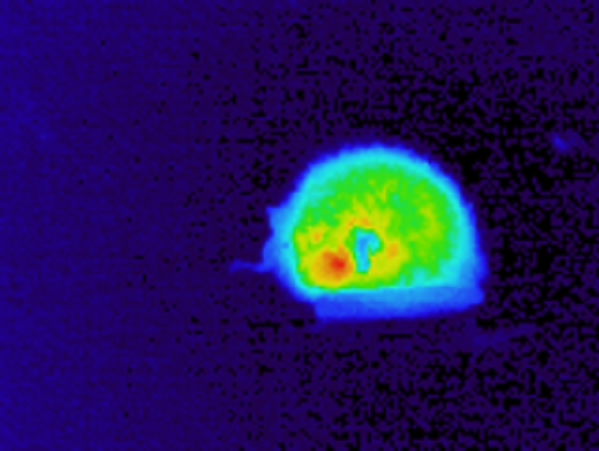

Supplement: Supplementary file 9 — Supplementary Slideshow S1.zip [file 41598_2018_30357_MOESM9_ESM.zip › Slideshow/photos/UTNB140427-170.jpg]

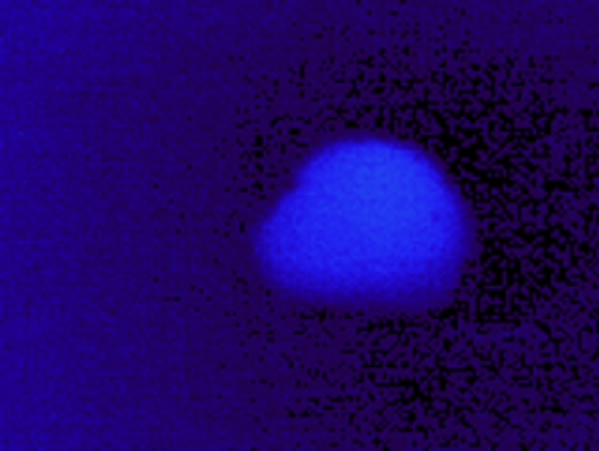

Supplement: Supplementary file 9 — Supplementary Slideshow S1.zip [file 41598_2018_30357_MOESM9_ESM.zip › Slideshow/photos/UTNB140427-038.jpg]

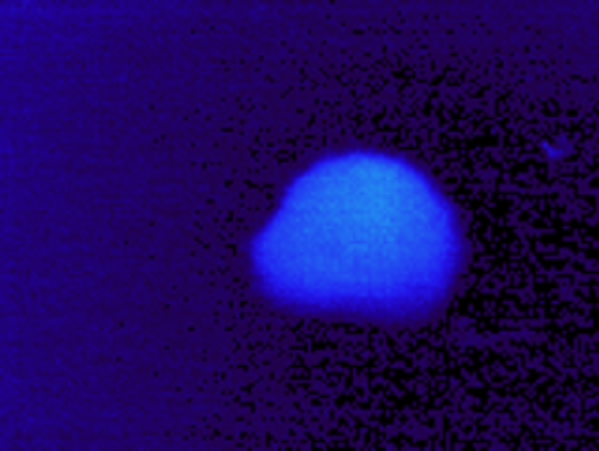

Supplement: Supplementary file 9 — Supplementary Slideshow S1.zip [file 41598_2018_30357_MOESM9_ESM.zip › Slideshow/photos/UTNB140427-010.jpg]

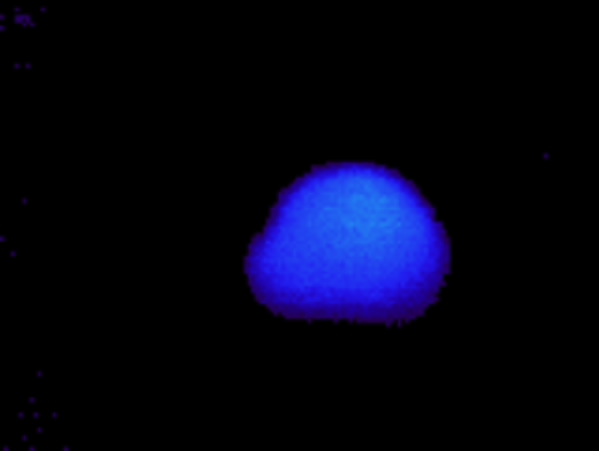

Supplement: Supplementary file 9 — Supplementary Slideshow S1.zip [file 41598_2018_30357_MOESM9_ESM.zip › Slideshow/photos/UTNB140427-004.jpg]

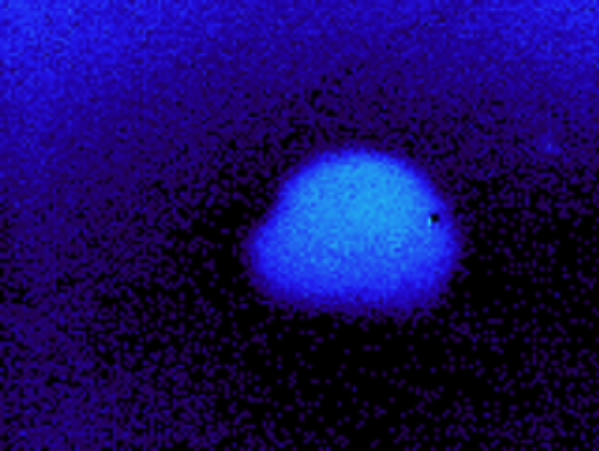

Supplement: Supplementary file 9 — Supplementary Slideshow S1.zip [file 41598_2018_30357_MOESM9_ESM.zip › Slideshow/photos/UTNB140427-005.jpg]

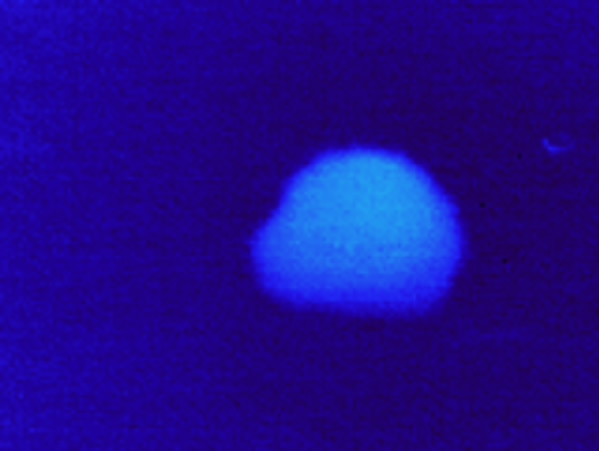

Supplement: Supplementary file 9 — Supplementary Slideshow S1.zip [file 41598_2018_30357_MOESM9_ESM.zip › Slideshow/photos/UTNB140427-011.jpg]

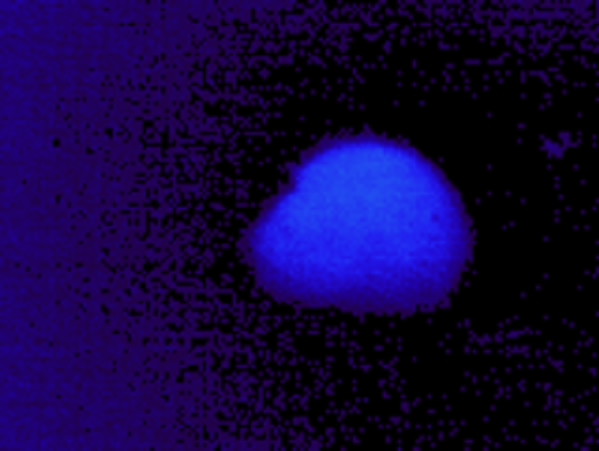

Supplement: Supplementary file 9 — Supplementary Slideshow S1.zip [file 41598_2018_30357_MOESM9_ESM.zip › Slideshow/photos/UTNB140427-039.jpg]

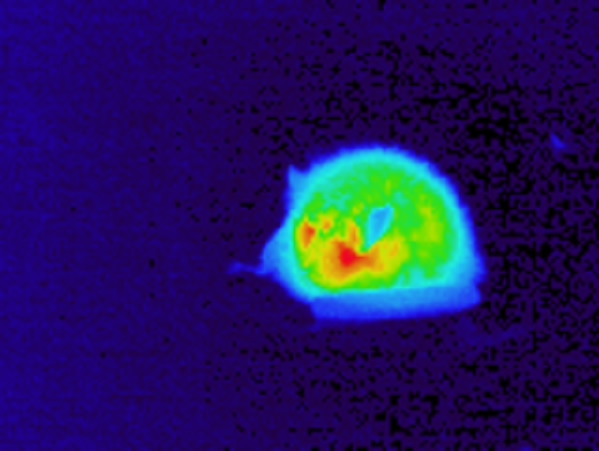

Supplement: Supplementary file 9 — Supplementary Slideshow S1.zip [file 41598_2018_30357_MOESM9_ESM.zip › Slideshow/photos/UTNB140427-171.jpg]

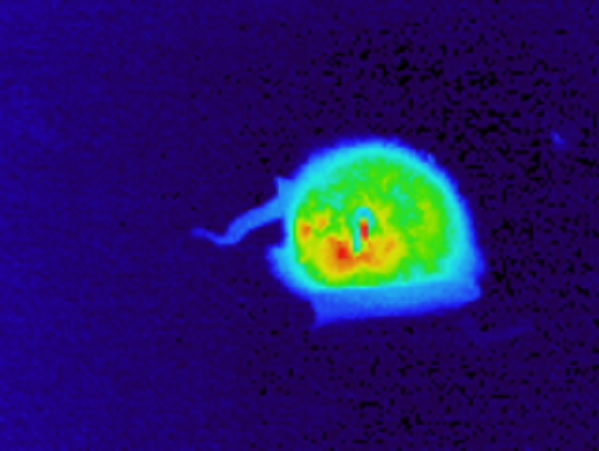

Supplement: Supplementary file 9 — Supplementary Slideshow S1.zip [file 41598_2018_30357_MOESM9_ESM.zip › Slideshow/photos/UTNB140427-165.jpg]

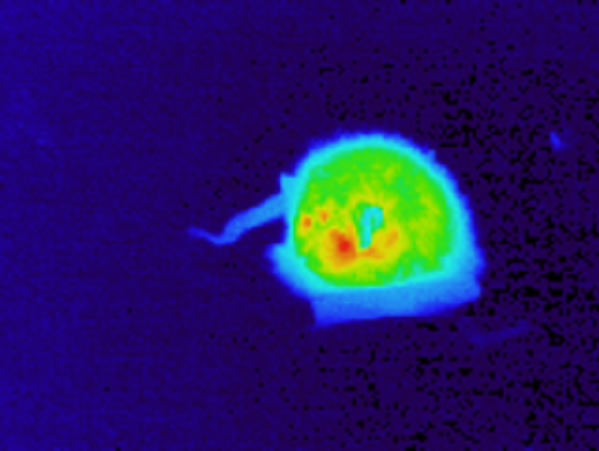

Supplement: Supplementary file 9 — Supplementary Slideshow S1.zip [file 41598_2018_30357_MOESM9_ESM.zip › Slideshow/photos/UTNB140427-159.jpg]

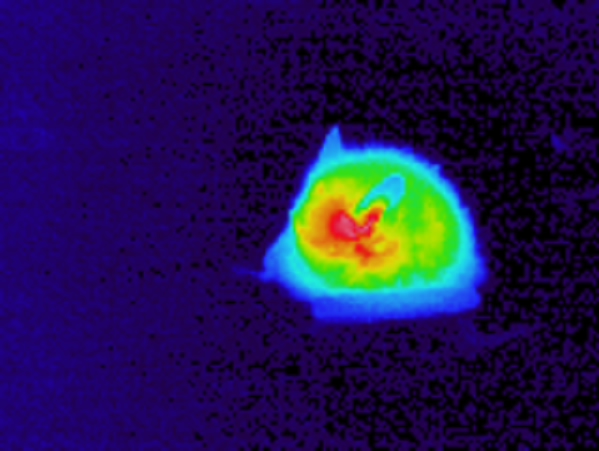

Supplement: Supplementary file 9 — Supplementary Slideshow S1.zip [file 41598_2018_30357_MOESM9_ESM.zip › Slideshow/photos/UTNB140427-173.jpg]

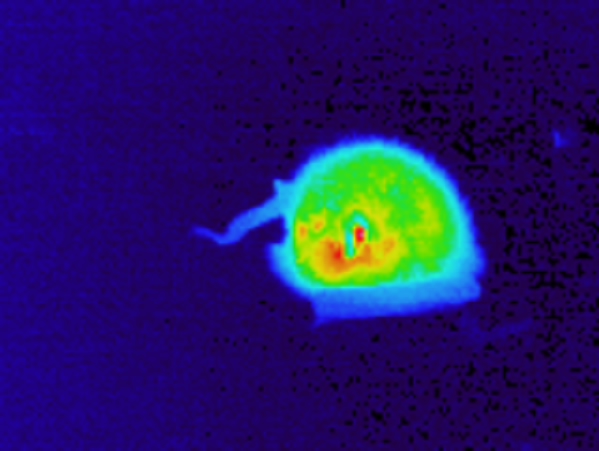

Supplement: Supplementary file 9 — Supplementary Slideshow S1.zip [file 41598_2018_30357_MOESM9_ESM.zip › Slideshow/photos/UTNB140427-167.jpg]

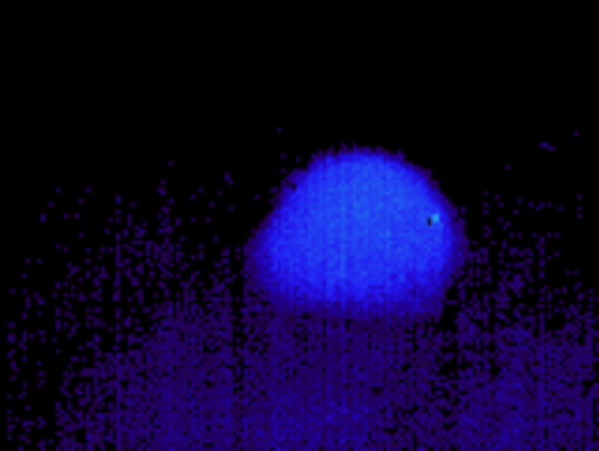

Supplement: Supplementary file 9 — Supplementary Slideshow S1.zip [file 41598_2018_30357_MOESM9_ESM.zip › Slideshow/photos/UTNB140427-007.jpg]

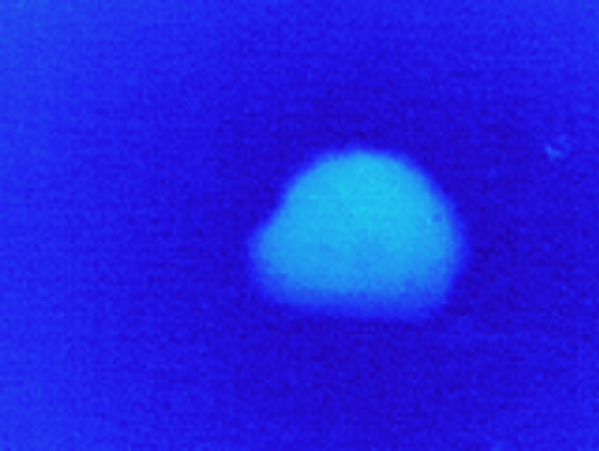

Supplement: Supplementary file 9 — Supplementary Slideshow S1.zip [file 41598_2018_30357_MOESM9_ESM.zip › Slideshow/photos/UTNB140427-013.jpg]

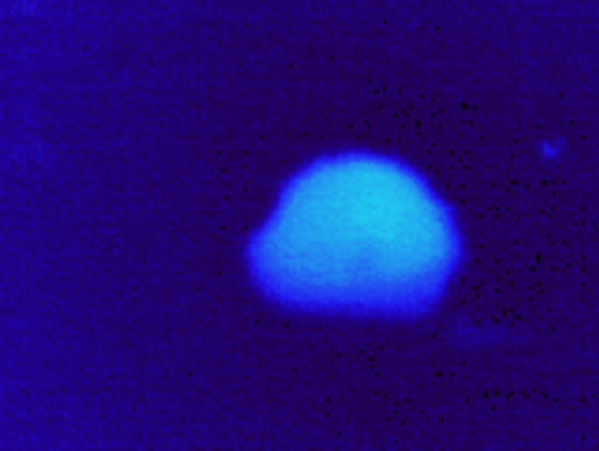

Supplement: Supplementary file 9 — Supplementary Slideshow S1.zip [file 41598_2018_30357_MOESM9_ESM.zip › Slideshow/photos/UTNB140427-006.jpg]

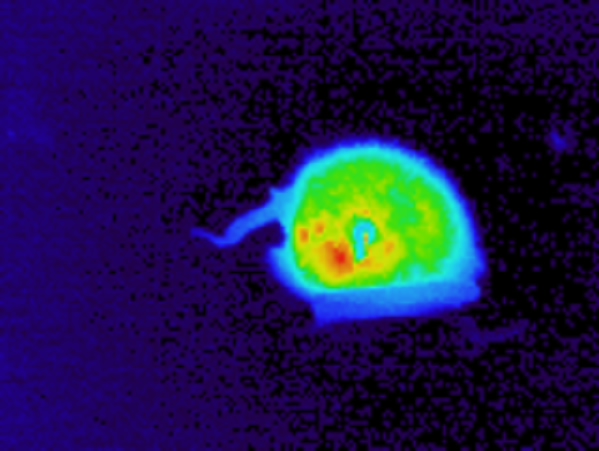

Supplement: Supplementary file 9 — Supplementary Slideshow S1.zip [file 41598_2018_30357_MOESM9_ESM.zip › Slideshow/photos/UTNB140427-166.jpg]

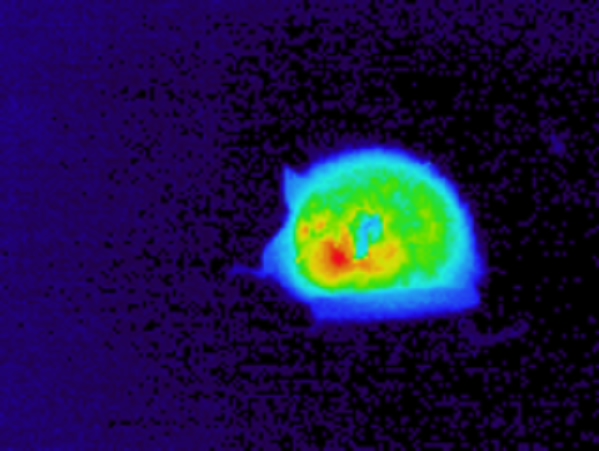

Supplement: Supplementary file 9 — Supplementary Slideshow S1.zip [file 41598_2018_30357_MOESM9_ESM.zip › Slideshow/photos/UTNB140427-172.jpg]

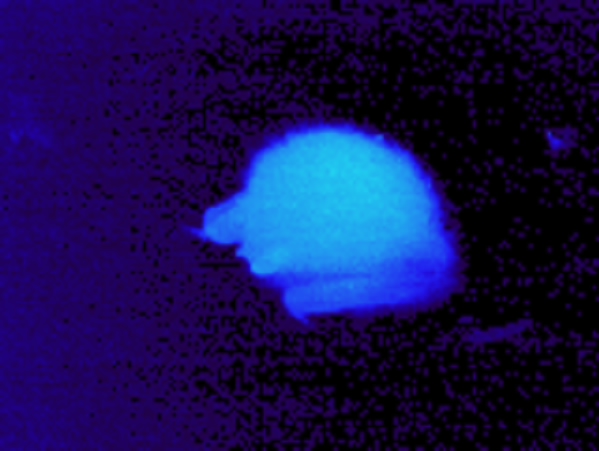

Supplement: Supplementary file 9 — Supplementary Slideshow S1.zip [file 41598_2018_30357_MOESM9_ESM.zip › Slideshow/photos/UTNB140427-115.jpg]

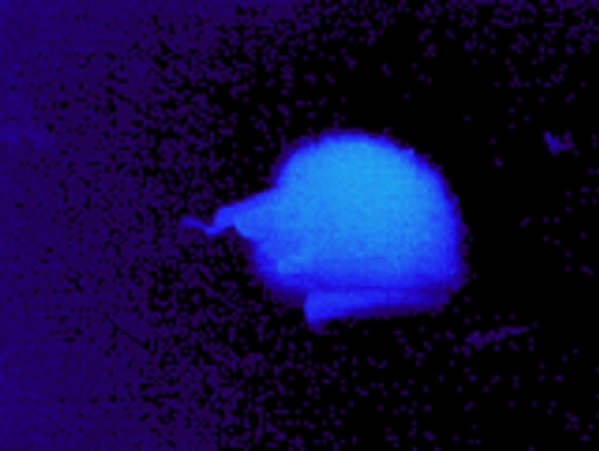

Supplement: Supplementary file 9 — Supplementary Slideshow S1.zip [file 41598_2018_30357_MOESM9_ESM.zip › Slideshow/photos/UTNB140427-101.jpg]

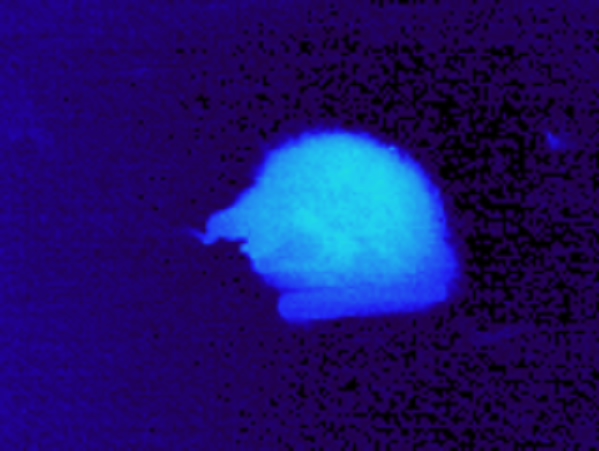

Supplement: Supplementary file 9 — Supplementary Slideshow S1.zip [file 41598_2018_30357_MOESM9_ESM.zip › Slideshow/photos/UTNB140427-129.jpg]

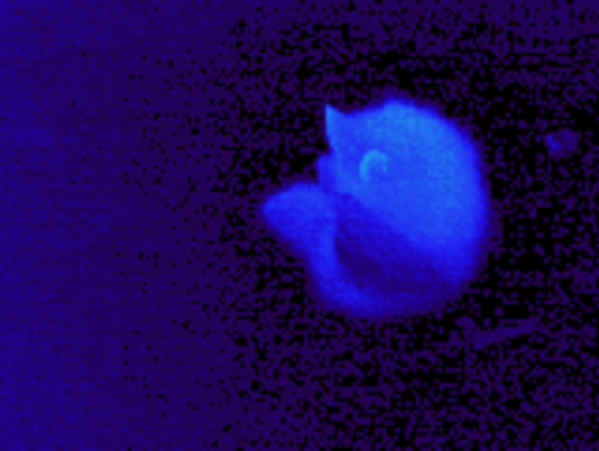

Supplement: Supplementary file 9 — Supplementary Slideshow S1.zip [file 41598_2018_30357_MOESM9_ESM.zip › Slideshow/photos/UTNB140427-061.jpg]

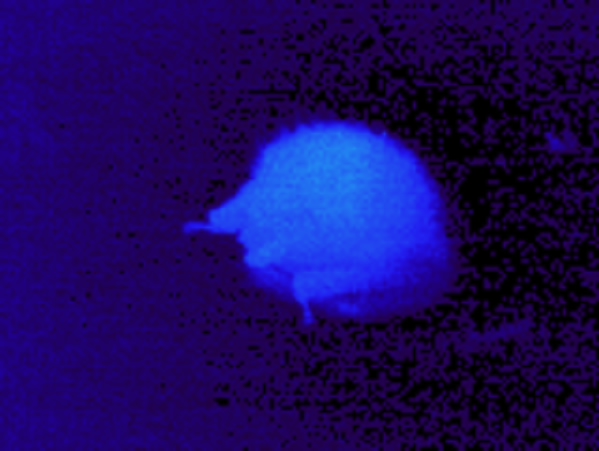

Supplement: Supplementary file 9 — Supplementary Slideshow S1.zip [file 41598_2018_30357_MOESM9_ESM.zip › Slideshow/photos/UTNB140427-075.jpg]

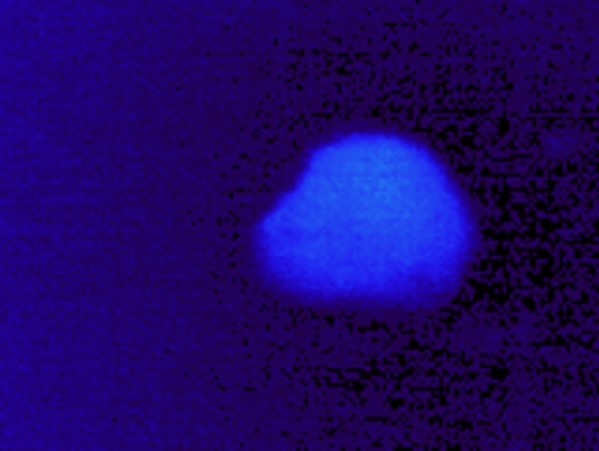

Supplement: Supplementary file 9 — Supplementary Slideshow S1.zip [file 41598_2018_30357_MOESM9_ESM.zip › Slideshow/photos/UTNB140427-049.jpg]

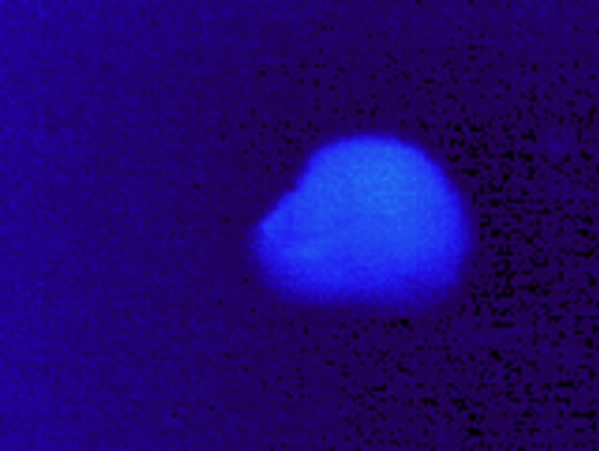

Supplement: Supplementary file 9 — Supplementary Slideshow S1.zip [file 41598_2018_30357_MOESM9_ESM.zip › Slideshow/photos/UTNB140427-048.jpg]

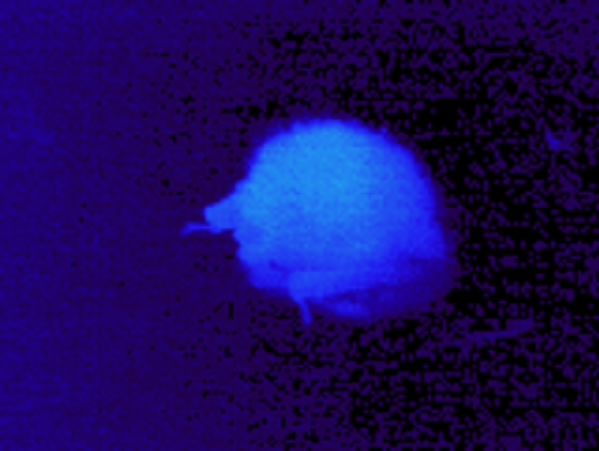

Supplement: Supplementary file 9 — Supplementary Slideshow S1.zip [file 41598_2018_30357_MOESM9_ESM.zip › Slideshow/photos/UTNB140427-074.jpg]

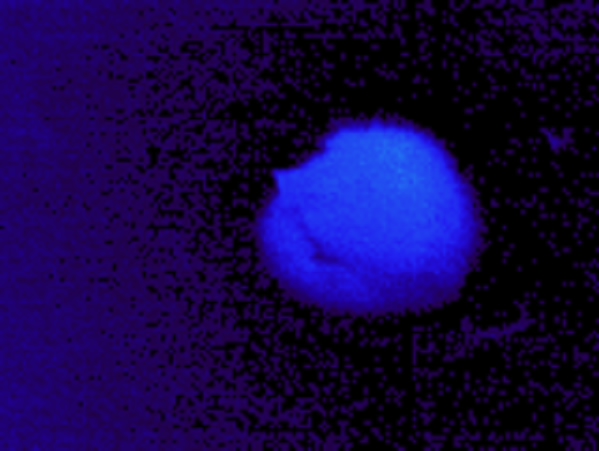

Supplement: Supplementary file 9 — Supplementary Slideshow S1.zip [file 41598_2018_30357_MOESM9_ESM.zip › Slideshow/photos/UTNB140427-060.jpg]

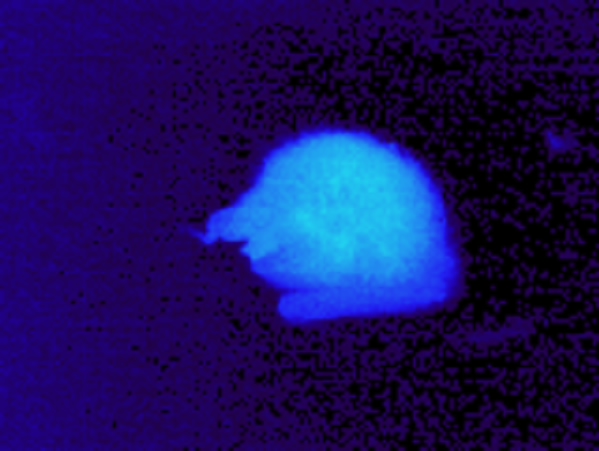

Supplement: Supplementary file 9 — Supplementary Slideshow S1.zip [file 41598_2018_30357_MOESM9_ESM.zip › Slideshow/photos/UTNB140427-128.jpg]

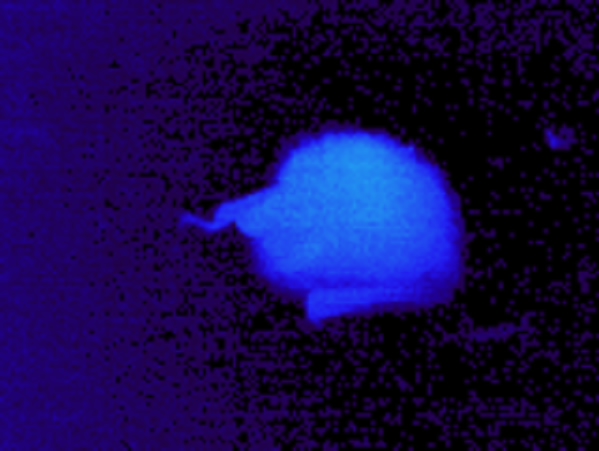

Supplement: Supplementary file 9 — Supplementary Slideshow S1.zip [file 41598_2018_30357_MOESM9_ESM.zip › Slideshow/photos/UTNB140427-100.jpg]

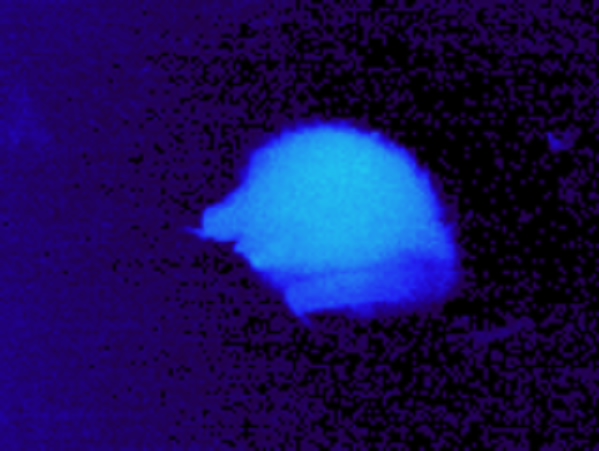

Supplement: Supplementary file 9 — Supplementary Slideshow S1.zip [file 41598_2018_30357_MOESM9_ESM.zip › Slideshow/photos/UTNB140427-114.jpg]

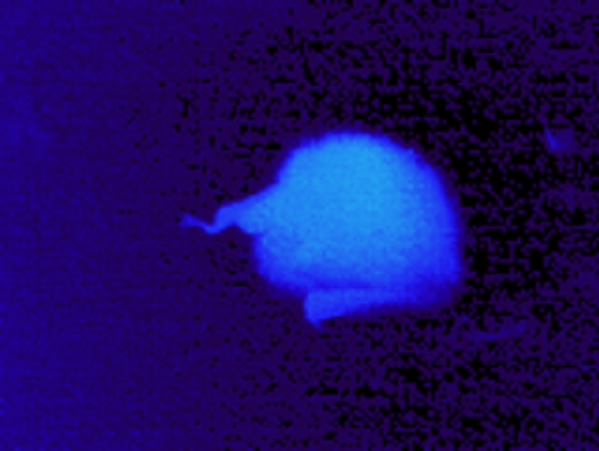

Supplement: Supplementary file 9 — Supplementary Slideshow S1.zip [file 41598_2018_30357_MOESM9_ESM.zip › Slideshow/photos/UTNB140427-102.jpg]

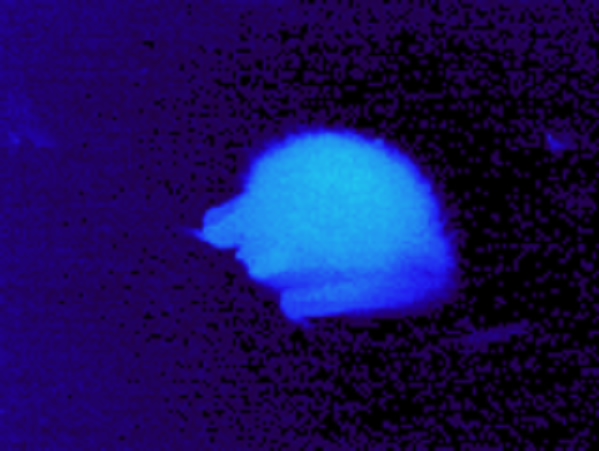

Supplement: Supplementary file 9 — Supplementary Slideshow S1.zip [file 41598_2018_30357_MOESM9_ESM.zip › Slideshow/photos/UTNB140427-116.jpg]

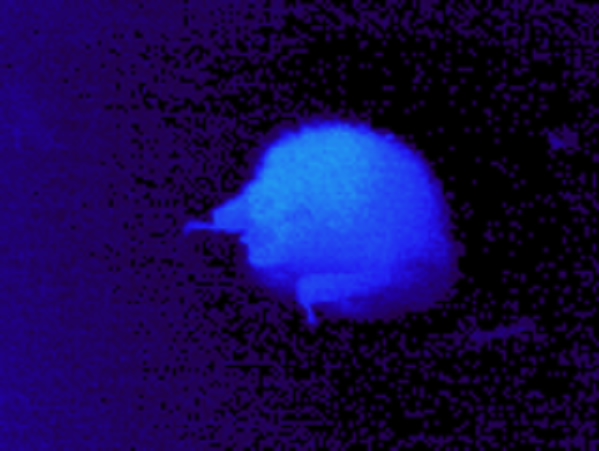

Supplement: Supplementary file 9 — Supplementary Slideshow S1.zip [file 41598_2018_30357_MOESM9_ESM.zip › Slideshow/photos/UTNB140427-076.jpg]

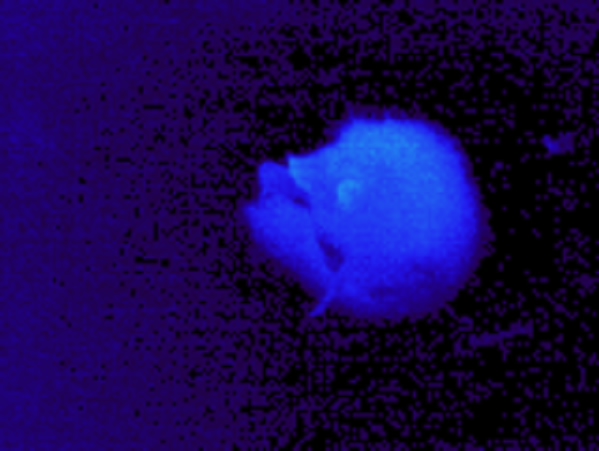

Supplement: Supplementary file 9 — Supplementary Slideshow S1.zip [file 41598_2018_30357_MOESM9_ESM.zip › Slideshow/photos/UTNB140427-062.jpg]

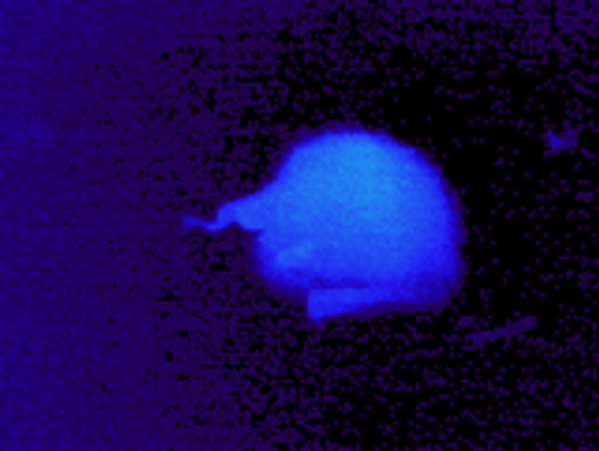

Supplement: Supplementary file 9 — Supplementary Slideshow S1.zip [file 41598_2018_30357_MOESM9_ESM.zip › Slideshow/photos/UTNB140427-089.jpg]

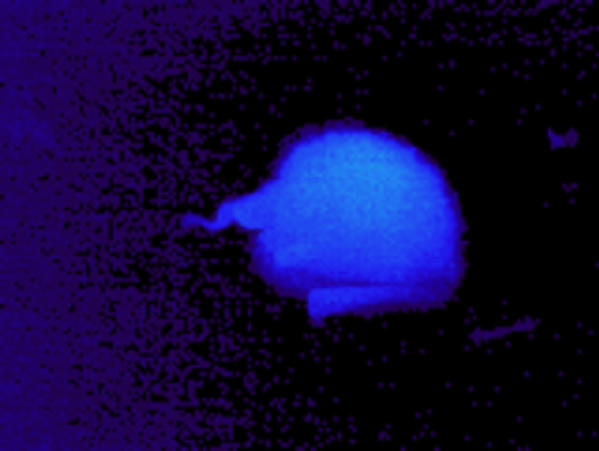

Supplement: Supplementary file 9 — Supplementary Slideshow S1.zip [file 41598_2018_30357_MOESM9_ESM.zip › Slideshow/photos/UTNB140427-088.jpg]

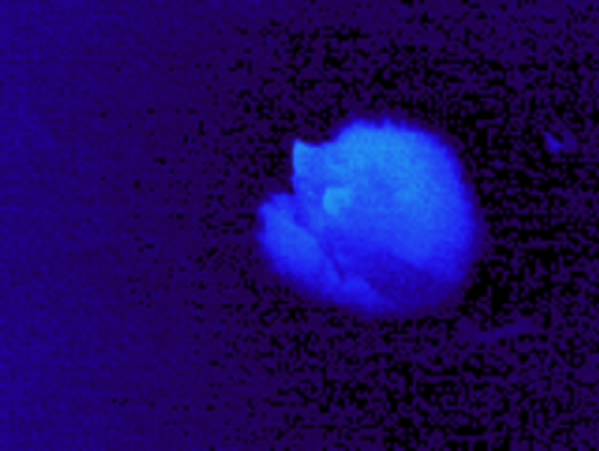

Supplement: Supplementary file 9 — Supplementary Slideshow S1.zip [file 41598_2018_30357_MOESM9_ESM.zip › Slideshow/photos/UTNB140427-063.jpg]

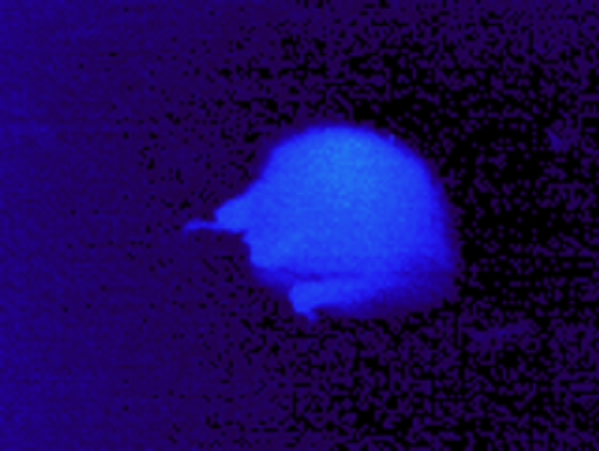

Supplement: Supplementary file 9 — Supplementary Slideshow S1.zip [file 41598_2018_30357_MOESM9_ESM.zip › Slideshow/photos/UTNB140427-077.jpg]

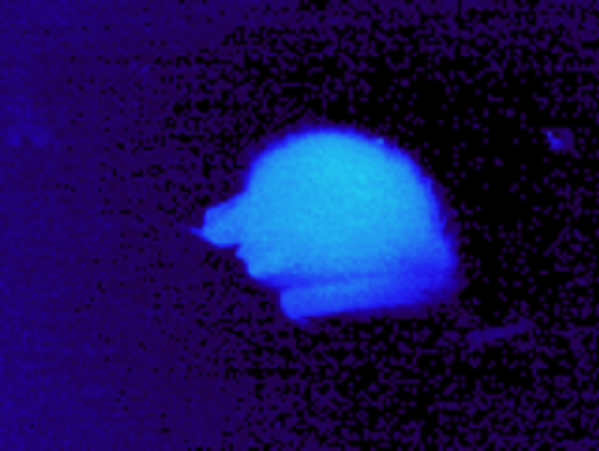

Supplement: Supplementary file 9 — Supplementary Slideshow S1.zip [file 41598_2018_30357_MOESM9_ESM.zip › Slideshow/photos/UTNB140427-117.jpg]

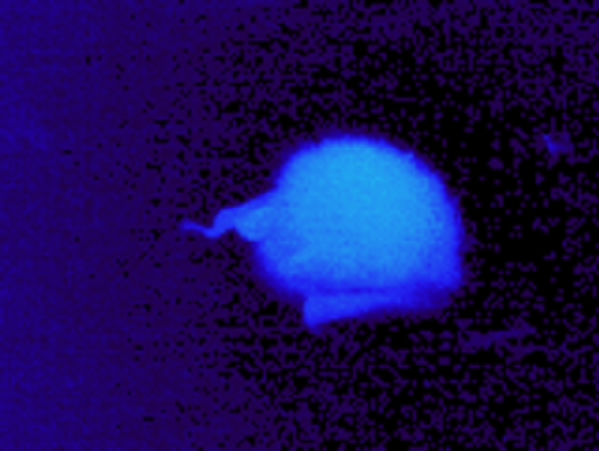

Supplement: Supplementary file 9 — Supplementary Slideshow S1.zip [file 41598_2018_30357_MOESM9_ESM.zip › Slideshow/photos/UTNB140427-103.jpg]

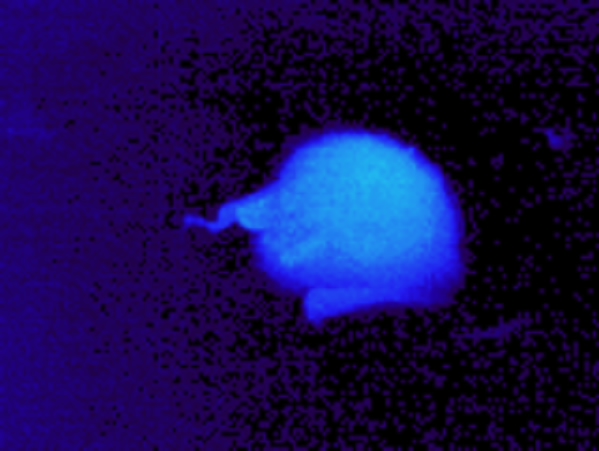

Supplement: Supplementary file 9 — Supplementary Slideshow S1.zip [file 41598_2018_30357_MOESM9_ESM.zip › Slideshow/photos/UTNB140427-107.jpg]

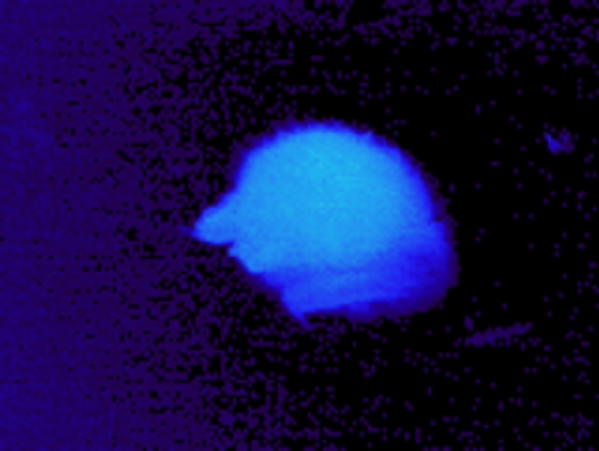

Supplement: Supplementary file 9 — Supplementary Slideshow S1.zip [file 41598_2018_30357_MOESM9_ESM.zip › Slideshow/photos/UTNB140427-113.jpg]

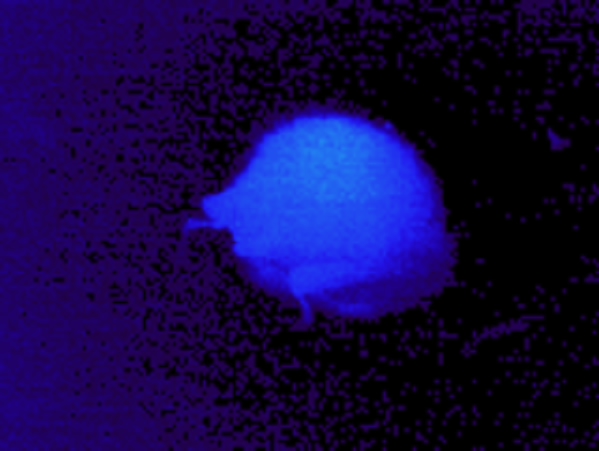

Supplement: Supplementary file 9 — Supplementary Slideshow S1.zip [file 41598_2018_30357_MOESM9_ESM.zip › Slideshow/photos/UTNB140427-073.jpg]

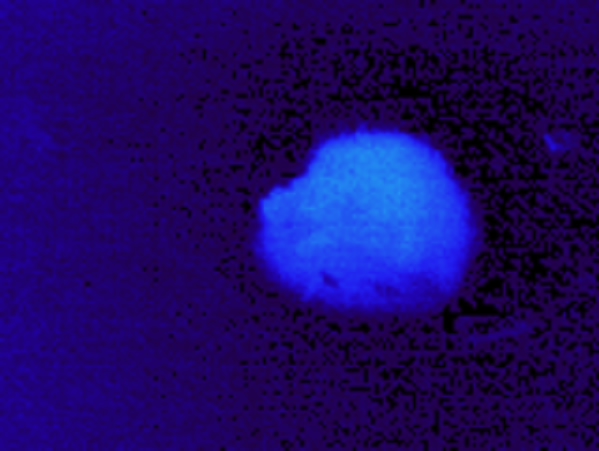

Supplement: Supplementary file 9 — Supplementary Slideshow S1.zip [file 41598_2018_30357_MOESM9_ESM.zip › Slideshow/photos/UTNB140427-067.jpg]

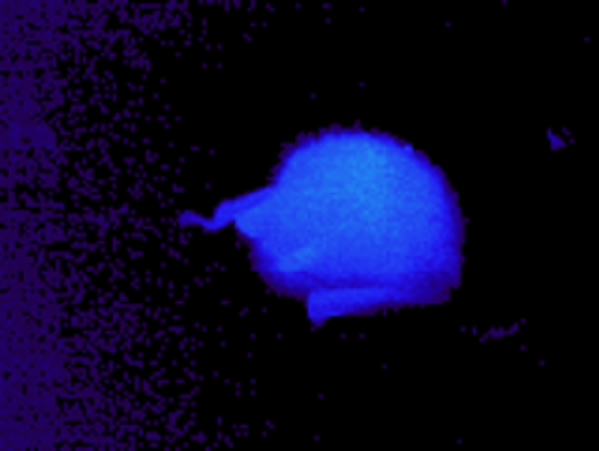

Supplement: Supplementary file 9 — Supplementary Slideshow S1.zip [file 41598_2018_30357_MOESM9_ESM.zip › Slideshow/photos/UTNB140427-098.jpg]

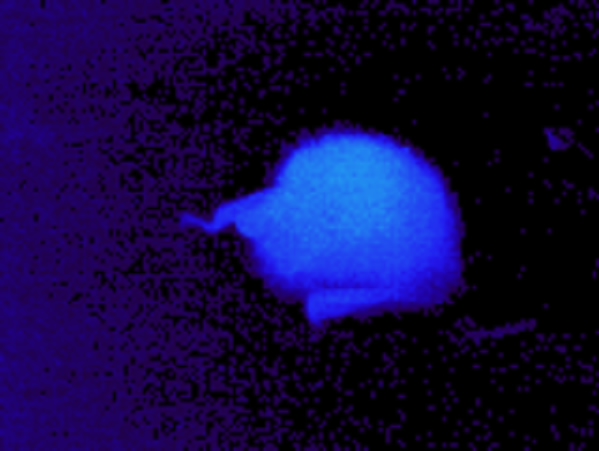

Supplement: Supplementary file 9 — Supplementary Slideshow S1.zip [file 41598_2018_30357_MOESM9_ESM.zip › Slideshow/photos/UTNB140427-099.jpg]

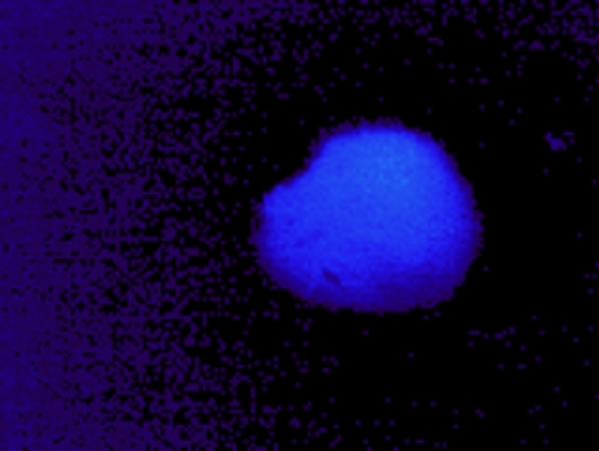

Supplement: Supplementary file 9 — Supplementary Slideshow S1.zip [file 41598_2018_30357_MOESM9_ESM.zip › Slideshow/photos/UTNB140427-066.jpg]

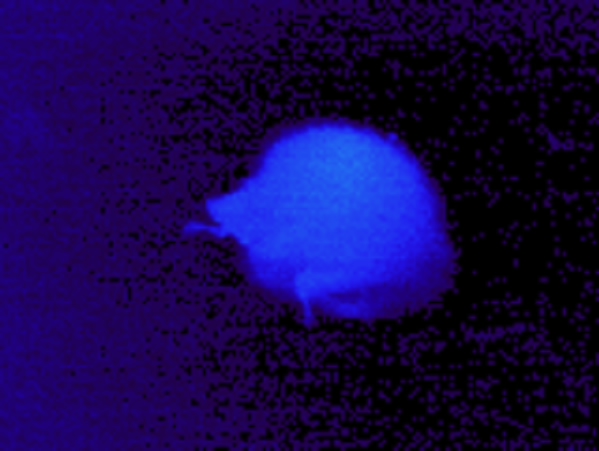

Supplement: Supplementary file 9 — Supplementary Slideshow S1.zip [file 41598_2018_30357_MOESM9_ESM.zip › Slideshow/photos/UTNB140427-072.jpg]

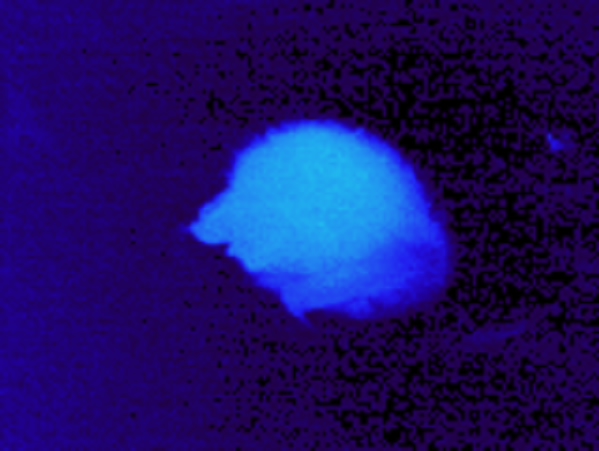

Supplement: Supplementary file 9 — Supplementary Slideshow S1.zip [file 41598_2018_30357_MOESM9_ESM.zip › Slideshow/photos/UTNB140427-112.jpg]

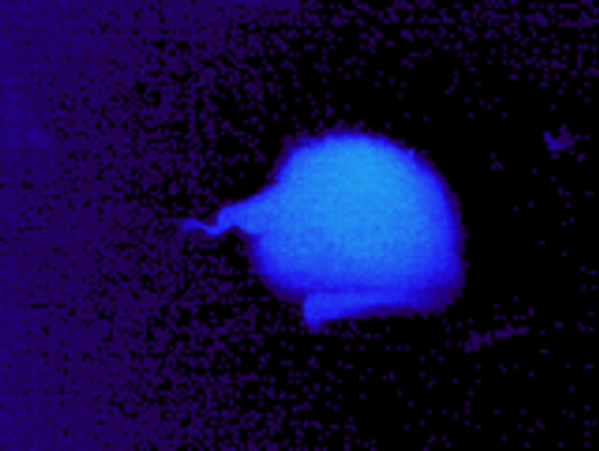

Supplement: Supplementary file 9 — Supplementary Slideshow S1.zip [file 41598_2018_30357_MOESM9_ESM.zip › Slideshow/photos/UTNB140427-106.jpg]

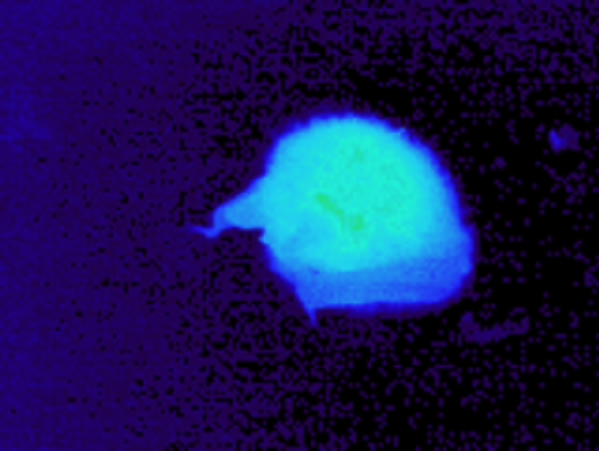

Supplement: Supplementary file 9 — Supplementary Slideshow S1.zip [file 41598_2018_30357_MOESM9_ESM.zip › Slideshow/photos/UTNB140427-138.jpg]

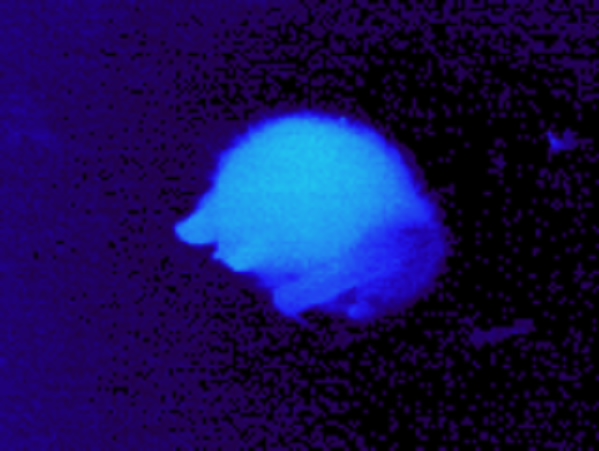

Supplement: Supplementary file 9 — Supplementary Slideshow S1.zip [file 41598_2018_30357_MOESM9_ESM.zip › Slideshow/photos/UTNB140427-110.jpg]

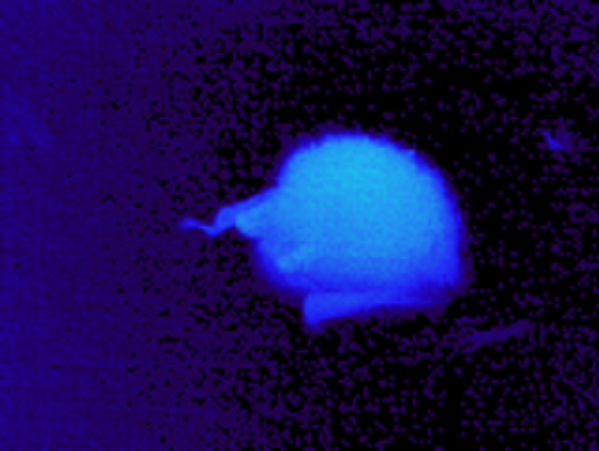

Supplement: Supplementary file 9 — Supplementary Slideshow S1.zip [file 41598_2018_30357_MOESM9_ESM.zip › Slideshow/photos/UTNB140427-104.jpg]

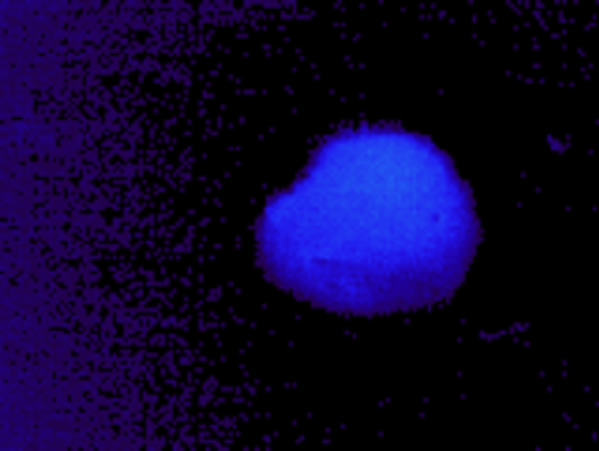

Supplement: Supplementary file 9 — Supplementary Slideshow S1.zip [file 41598_2018_30357_MOESM9_ESM.zip › Slideshow/photos/UTNB140427-058.jpg]

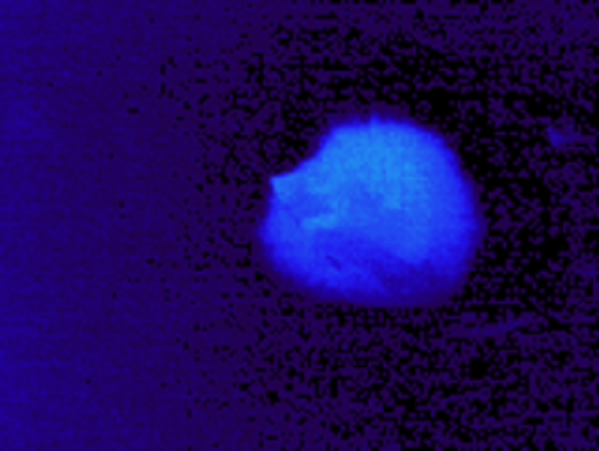

Supplement: Supplementary file 9 — Supplementary Slideshow S1.zip [file 41598_2018_30357_MOESM9_ESM.zip › Slideshow/photos/UTNB140427-064.jpg]

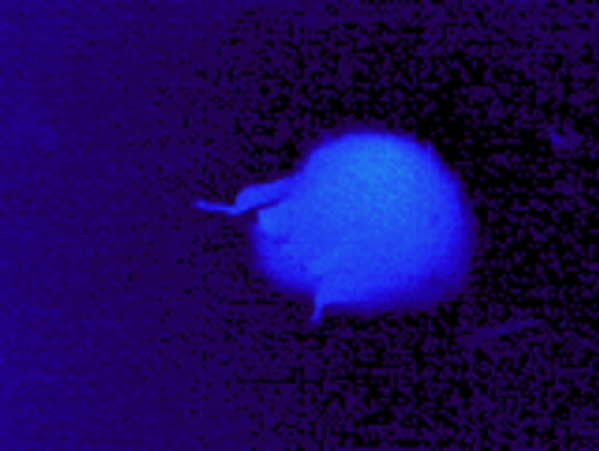

Supplement: Supplementary file 9 — Supplementary Slideshow S1.zip [file 41598_2018_30357_MOESM9_ESM.zip › Slideshow/photos/UTNB140427-070.jpg]

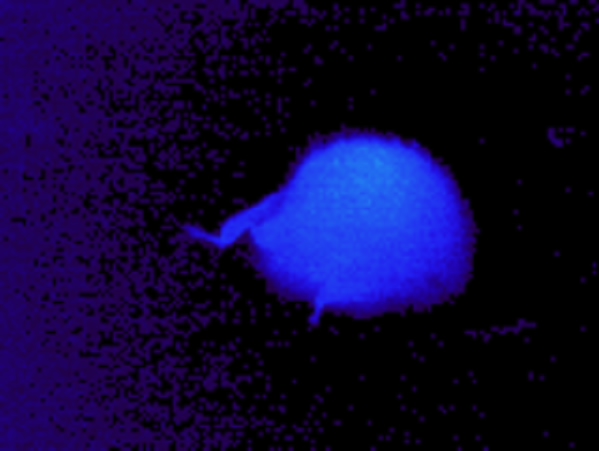

Supplement: Supplementary file 9 — Supplementary Slideshow S1.zip [file 41598_2018_30357_MOESM9_ESM.zip › Slideshow/photos/UTNB140427-071.jpg]

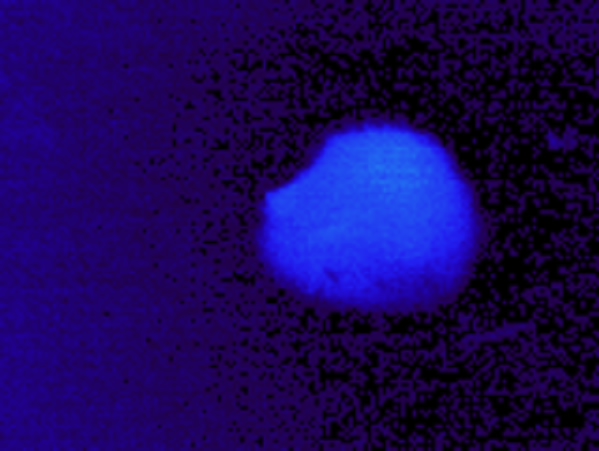

Supplement: Supplementary file 9 — Supplementary Slideshow S1.zip [file 41598_2018_30357_MOESM9_ESM.zip › Slideshow/photos/UTNB140427-065.jpg]

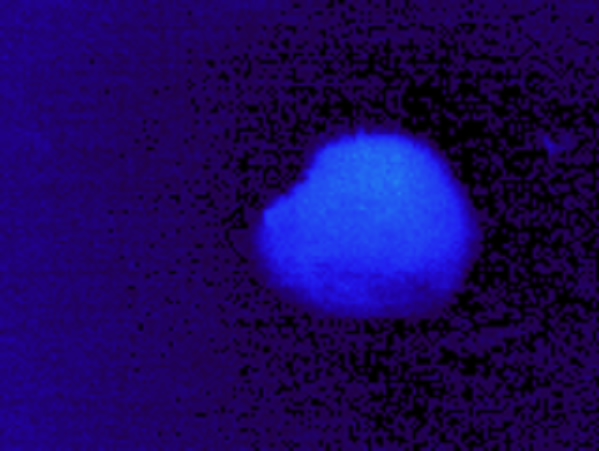

Supplement: Supplementary file 9 — Supplementary Slideshow S1.zip [file 41598_2018_30357_MOESM9_ESM.zip › Slideshow/photos/UTNB140427-059.jpg]

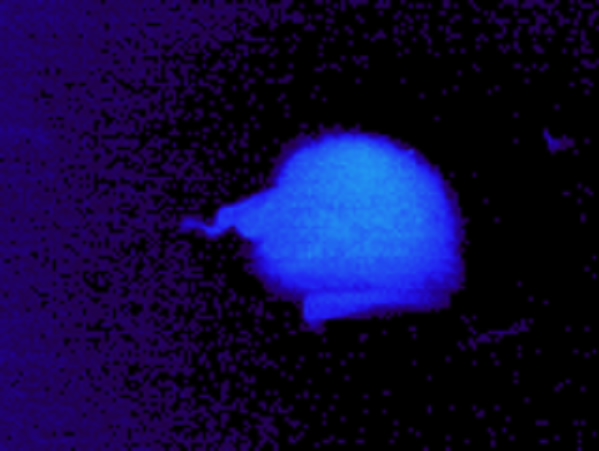

Supplement: Supplementary file 9 — Supplementary Slideshow S1.zip [file 41598_2018_30357_MOESM9_ESM.zip › Slideshow/photos/UTNB140427-105.jpg]

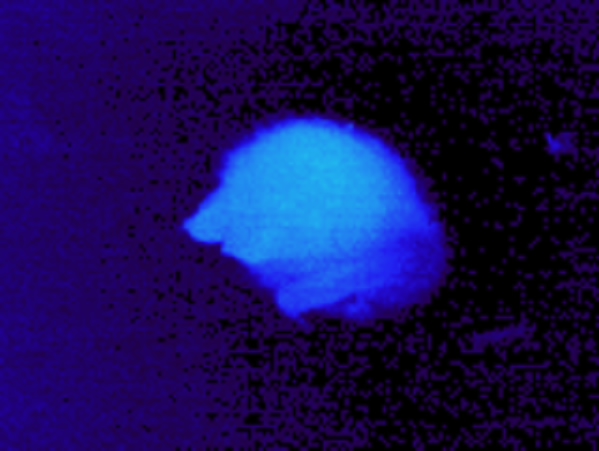

Supplement: Supplementary file 9 — Supplementary Slideshow S1.zip [file 41598_2018_30357_MOESM9_ESM.zip › Slideshow/photos/UTNB140427-111.jpg]

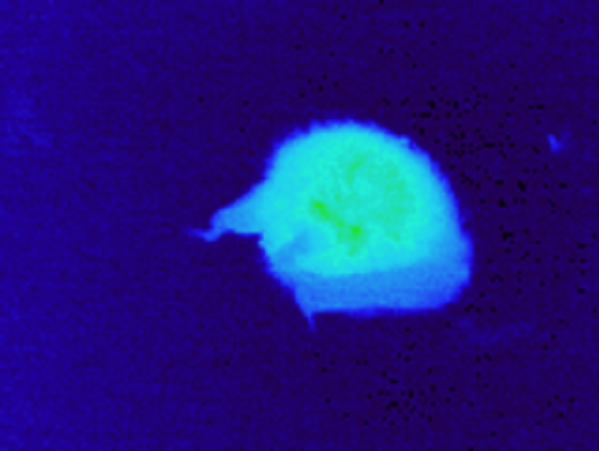

Supplement: Supplementary file 9 — Supplementary Slideshow S1.zip [file 41598_2018_30357_MOESM9_ESM.zip › Slideshow/photos/UTNB140427-139.jpg]
