# Supplementary figures and images for: Evidence for Ussurian tube-nosed bats (Murina ussuriensis) hibernating in snow (part 1 of 2)
Source: Sci Rep. 2018 Aug 13;8:12047. doi: 10.1038/s41598-018-30357-1 (PMC6089880; doi:10.1038/s41598-018-30357-1)

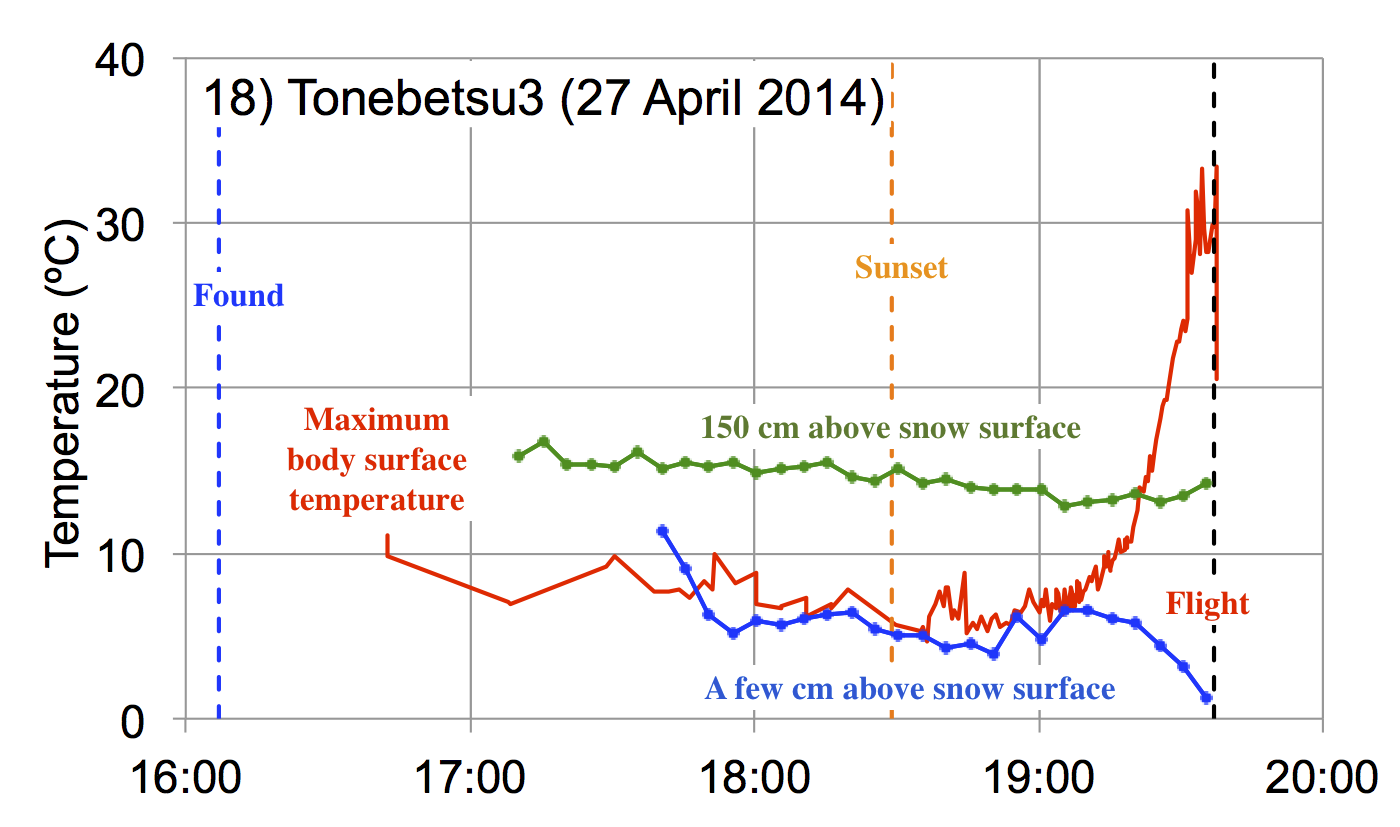

Supplement: Supplementary file 9 — Supplementary Slideshow S1.zip [file 41598_2018_30357_MOESM9_ESM.zip › Slideshow/figures/SumChart2.png]

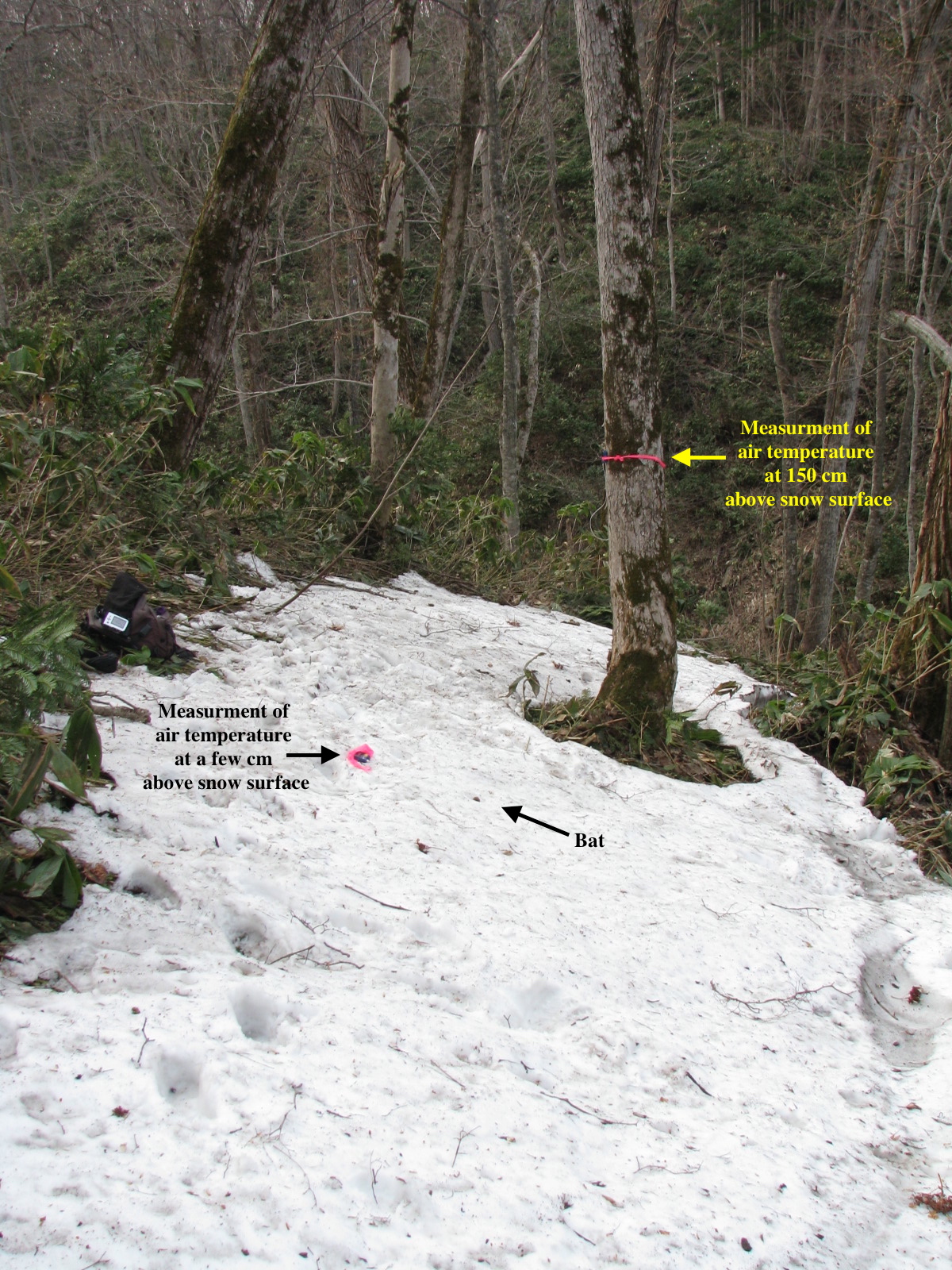

Supplement: Supplementary file 9 — Supplementary Slideshow S1.zip [file 41598_2018_30357_MOESM9_ESM.zip › Slideshow/figures/IMG_2908.jpg]

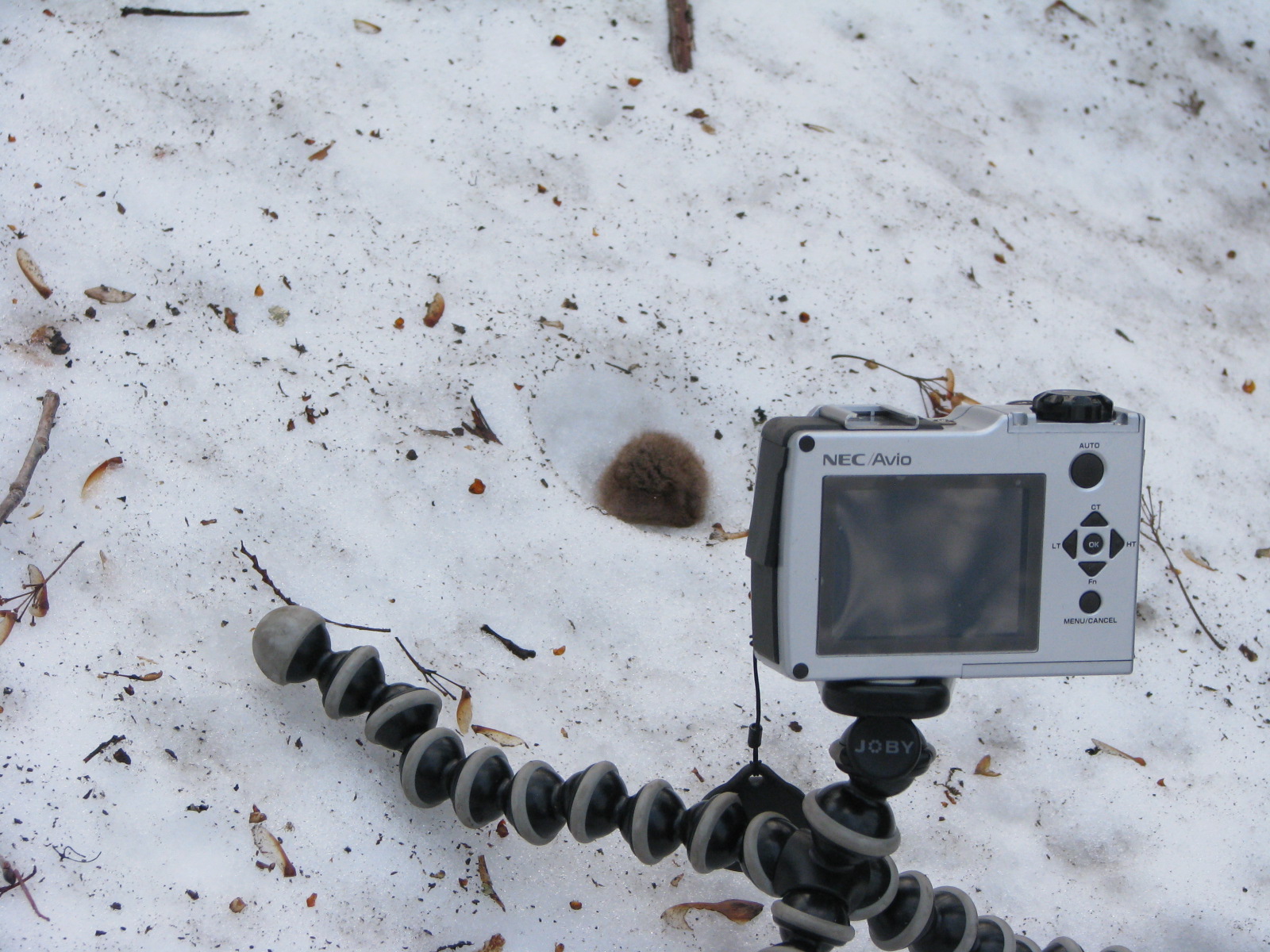

Supplement: Supplementary file 9 — Supplementary Slideshow S1.zip [file 41598_2018_30357_MOESM9_ESM.zip › Slideshow/figures/IMG_2915.JPG]

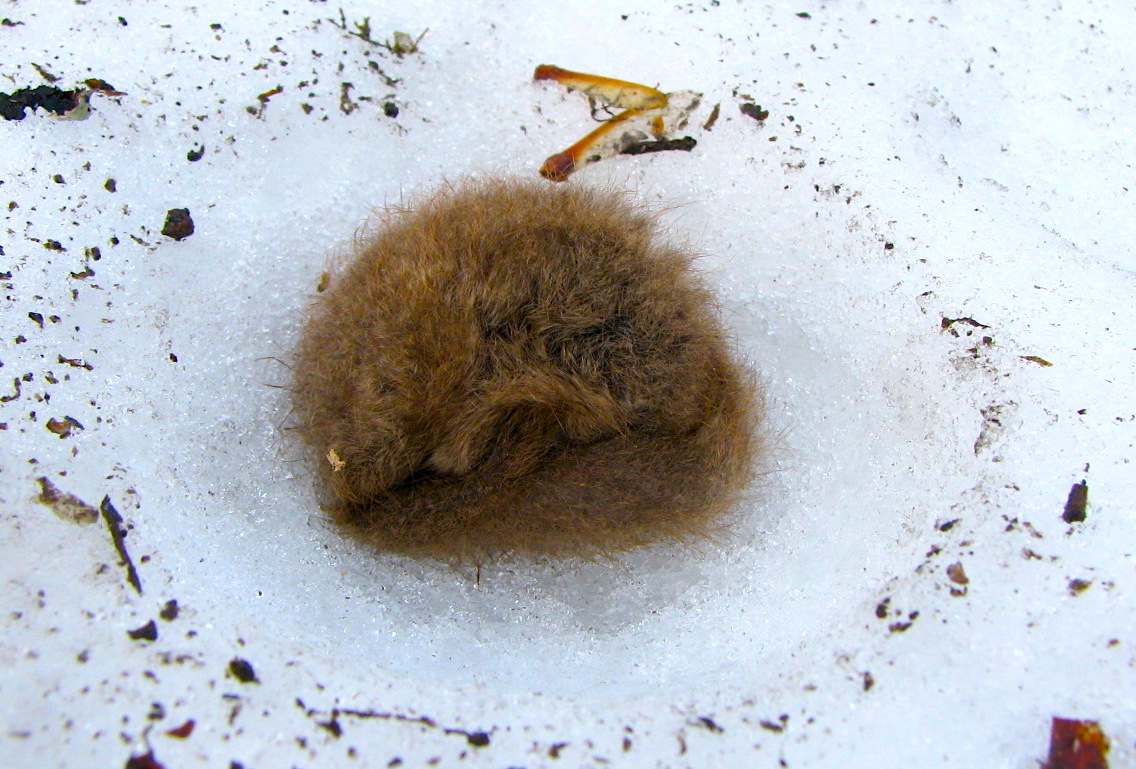

Supplement: Supplementary file 9 — Supplementary Slideshow S1.zip [file 41598_2018_30357_MOESM9_ESM.zip › Slideshow/figures/cover.jpg]

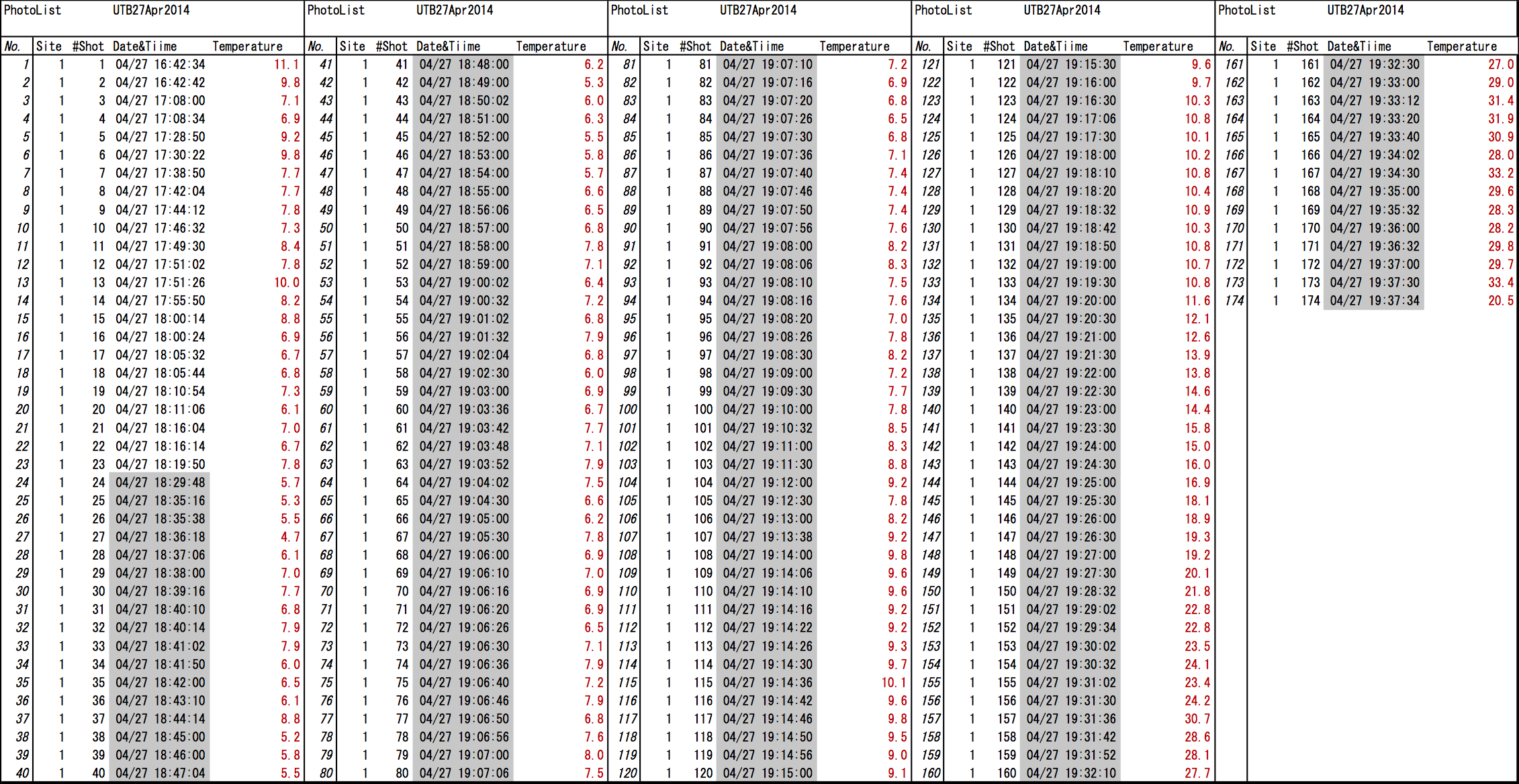

Supplement: Supplementary file 9 — Supplementary Slideshow S1.zip [file 41598_2018_30357_MOESM9_ESM.zip › Slideshow/figures/PhotoList.png]

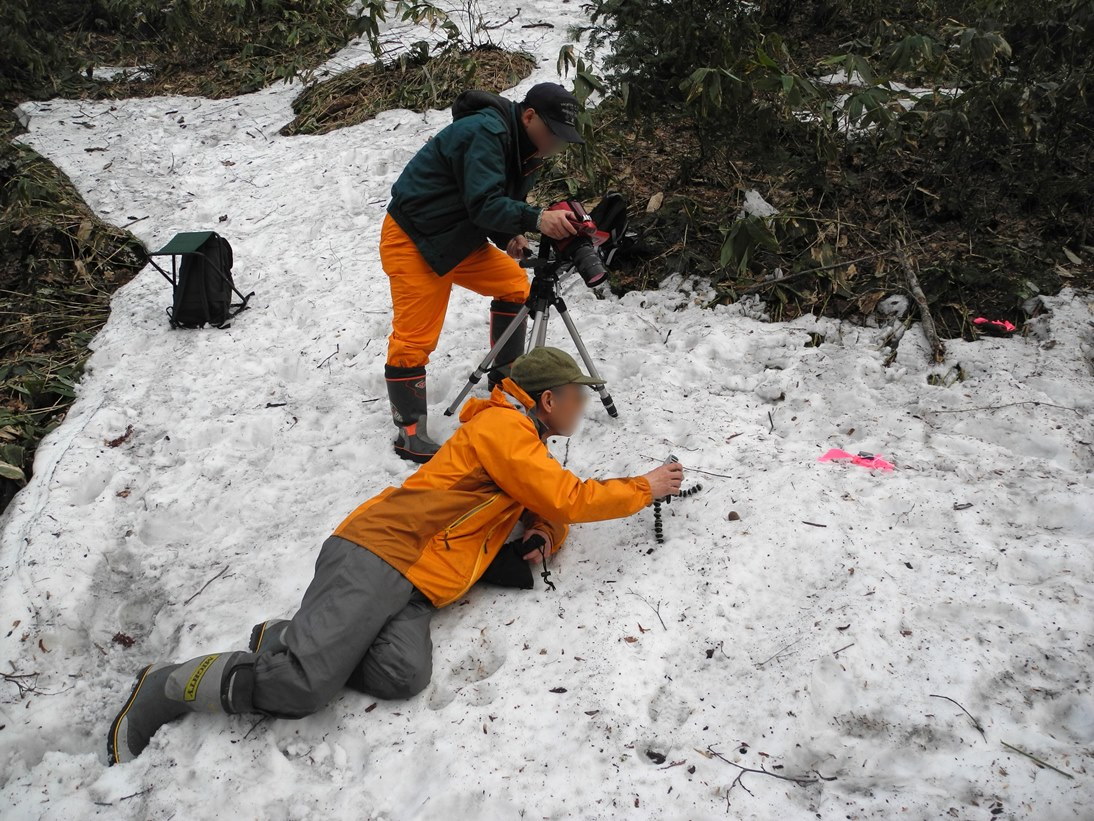

Supplement: Supplementary file 9 — Supplementary Slideshow S1.zip [file 41598_2018_30357_MOESM9_ESM.zip › Slideshow/figures/nagasaka2.jpg]

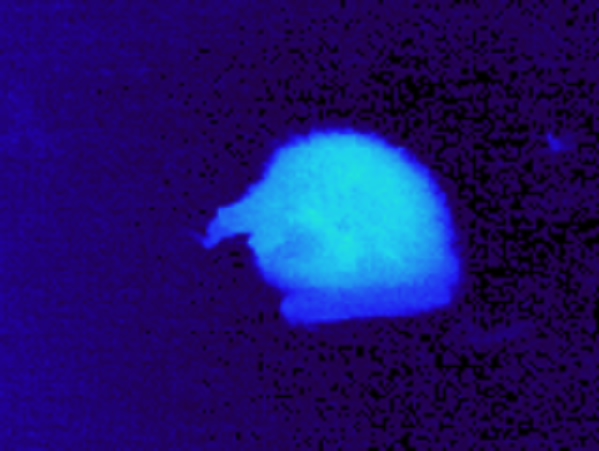

Supplement: Supplementary file 9 — Supplementary Slideshow S1.zip [file 41598_2018_30357_MOESM9_ESM.zip › Slideshow/photos/UTNB140427-134.jpg]

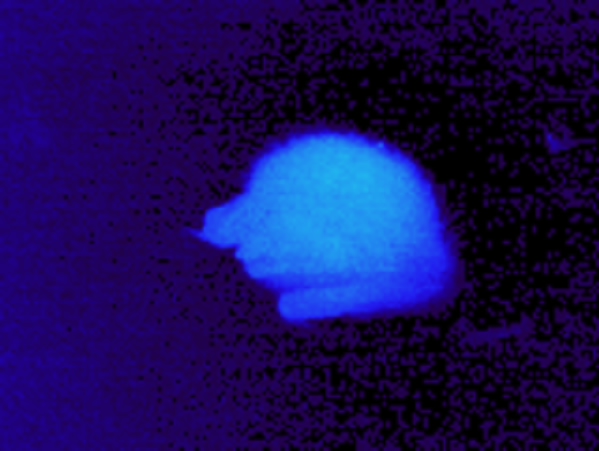

Supplement: Supplementary file 9 — Supplementary Slideshow S1.zip [file 41598_2018_30357_MOESM9_ESM.zip › Slideshow/photos/UTNB140427-120.jpg]

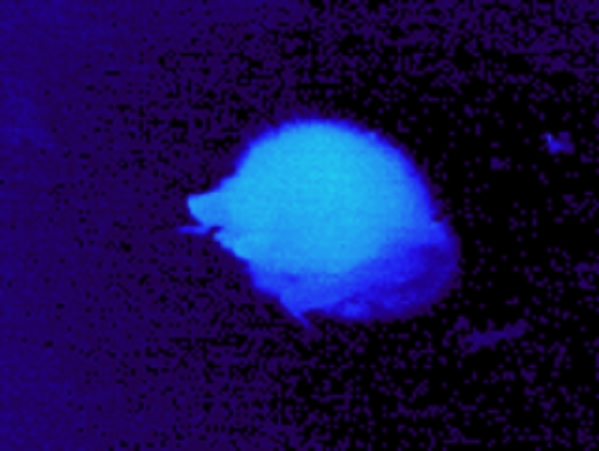

Supplement: Supplementary file 9 — Supplementary Slideshow S1.zip [file 41598_2018_30357_MOESM9_ESM.zip › Slideshow/photos/UTNB140427-108.jpg]

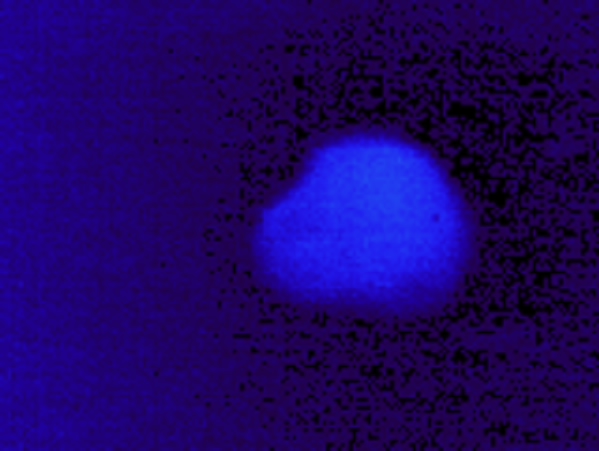

Supplement: Supplementary file 9 — Supplementary Slideshow S1.zip [file 41598_2018_30357_MOESM9_ESM.zip › Slideshow/photos/UTNB140427-040.jpg]

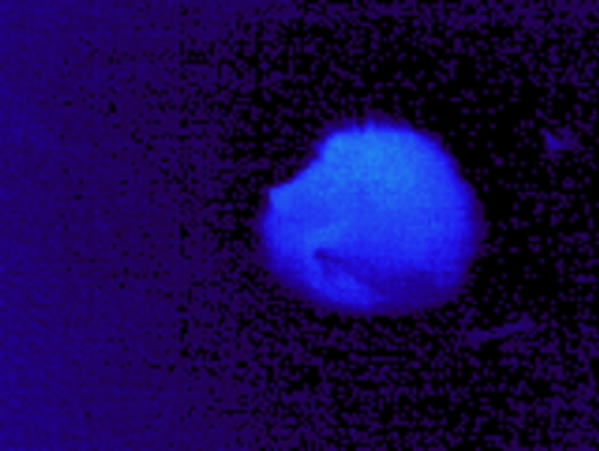

Supplement: Supplementary file 9 — Supplementary Slideshow S1.zip [file 41598_2018_30357_MOESM9_ESM.zip › Slideshow/photos/UTNB140427-054.jpg]

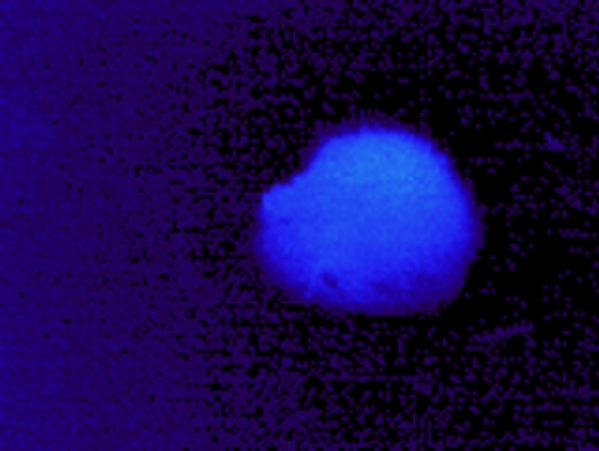

Supplement: Supplementary file 9 — Supplementary Slideshow S1.zip [file 41598_2018_30357_MOESM9_ESM.zip › Slideshow/photos/UTNB140427-068.jpg]

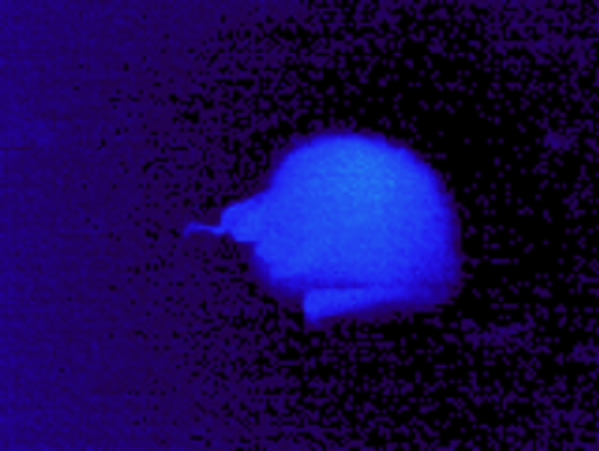

Supplement: Supplementary file 9 — Supplementary Slideshow S1.zip [file 41598_2018_30357_MOESM9_ESM.zip › Slideshow/photos/UTNB140427-083.jpg]

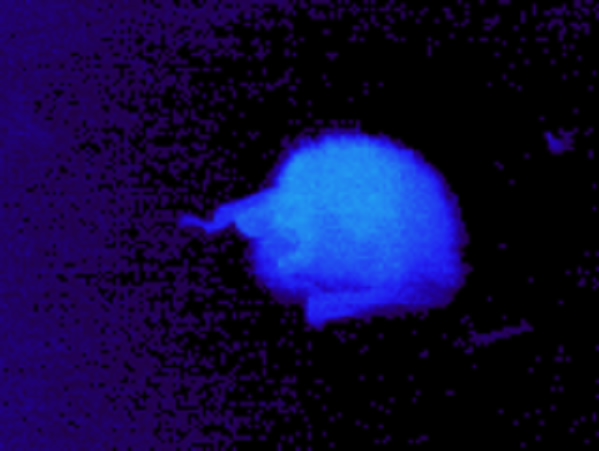

Supplement: Supplementary file 9 — Supplementary Slideshow S1.zip [file 41598_2018_30357_MOESM9_ESM.zip › Slideshow/photos/UTNB140427-097.jpg]

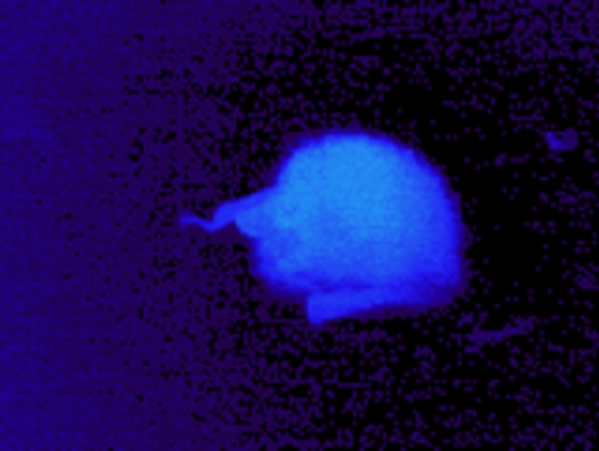

Supplement: Supplementary file 9 — Supplementary Slideshow S1.zip [file 41598_2018_30357_MOESM9_ESM.zip › Slideshow/photos/UTNB140427-096.jpg]

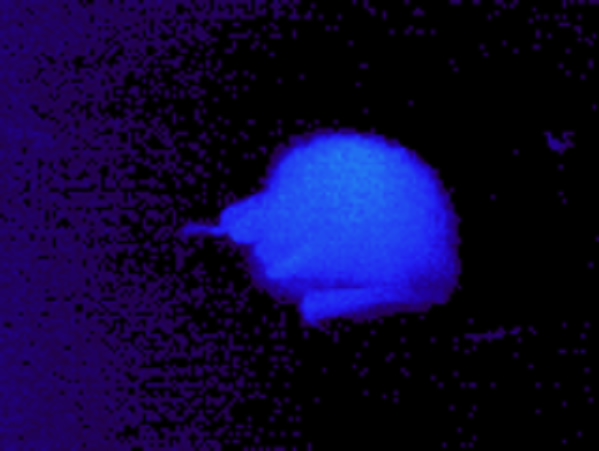

Supplement: Supplementary file 9 — Supplementary Slideshow S1.zip [file 41598_2018_30357_MOESM9_ESM.zip › Slideshow/photos/UTNB140427-082.jpg]

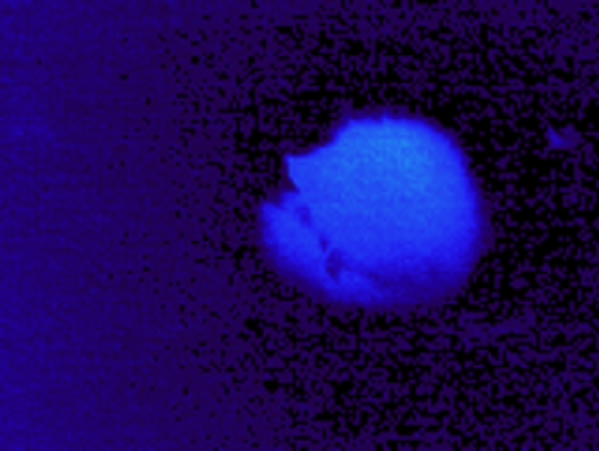

Supplement: Supplementary file 9 — Supplementary Slideshow S1.zip [file 41598_2018_30357_MOESM9_ESM.zip › Slideshow/photos/UTNB140427-069.jpg]

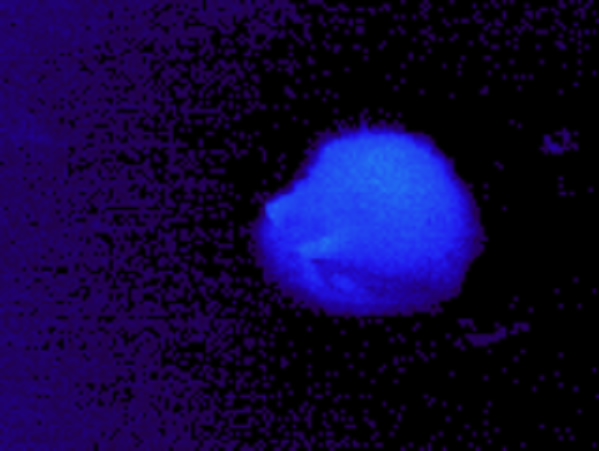

Supplement: Supplementary file 9 — Supplementary Slideshow S1.zip [file 41598_2018_30357_MOESM9_ESM.zip › Slideshow/photos/UTNB140427-055.jpg]

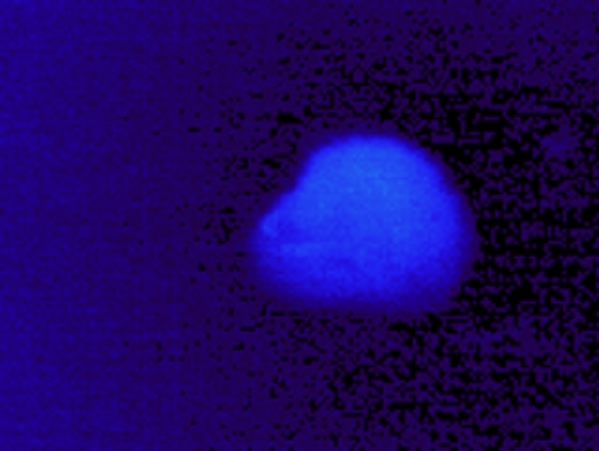

Supplement: Supplementary file 9 — Supplementary Slideshow S1.zip [file 41598_2018_30357_MOESM9_ESM.zip › Slideshow/photos/UTNB140427-041.jpg]

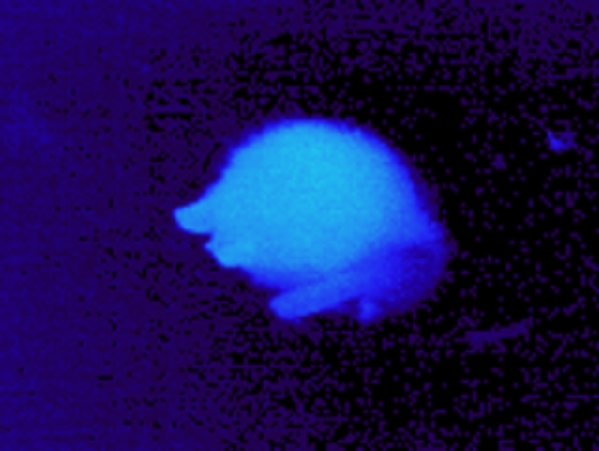

Supplement: Supplementary file 9 — Supplementary Slideshow S1.zip [file 41598_2018_30357_MOESM9_ESM.zip › Slideshow/photos/UTNB140427-109.jpg]

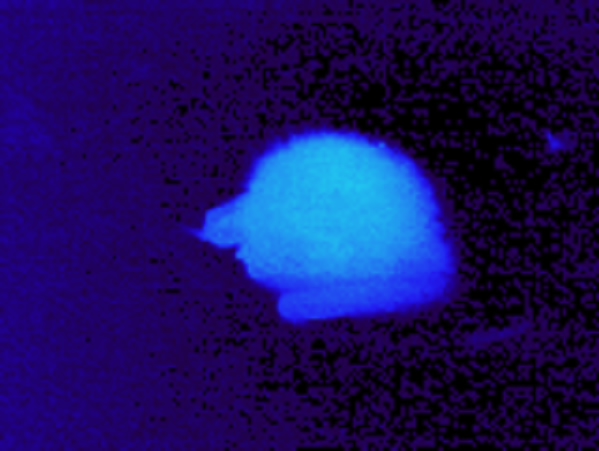

Supplement: Supplementary file 9 — Supplementary Slideshow S1.zip [file 41598_2018_30357_MOESM9_ESM.zip › Slideshow/photos/UTNB140427-121.jpg]

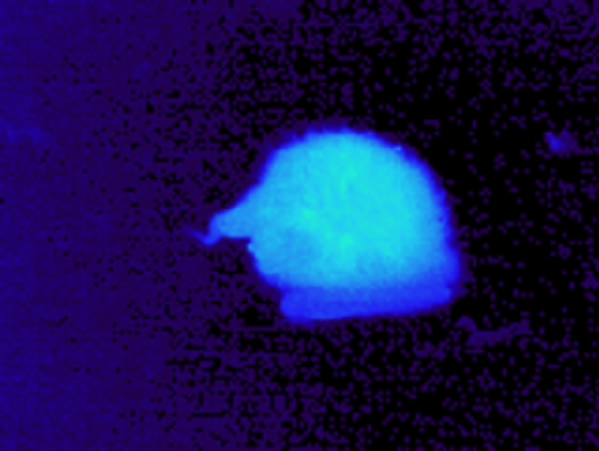

Supplement: Supplementary file 9 — Supplementary Slideshow S1.zip [file 41598_2018_30357_MOESM9_ESM.zip › Slideshow/photos/UTNB140427-135.jpg]

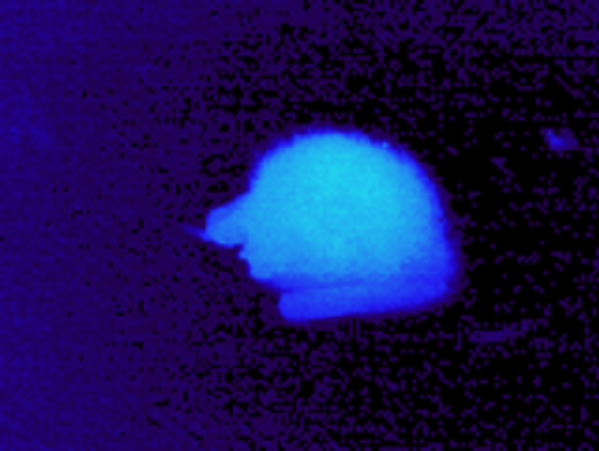

Supplement: Supplementary file 9 — Supplementary Slideshow S1.zip [file 41598_2018_30357_MOESM9_ESM.zip › Slideshow/photos/UTNB140427-123.jpg]

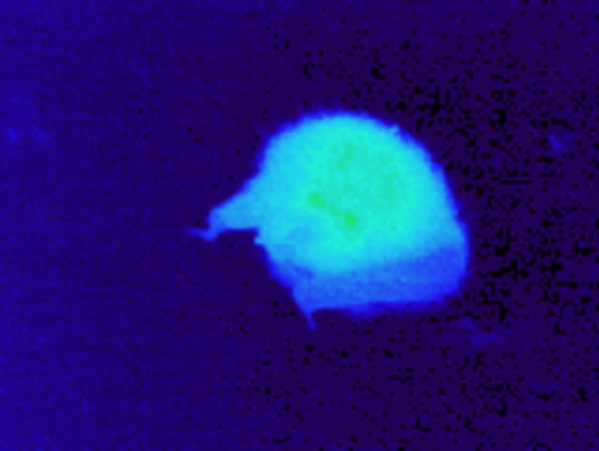

Supplement: Supplementary file 9 — Supplementary Slideshow S1.zip [file 41598_2018_30357_MOESM9_ESM.zip › Slideshow/photos/UTNB140427-137.jpg]

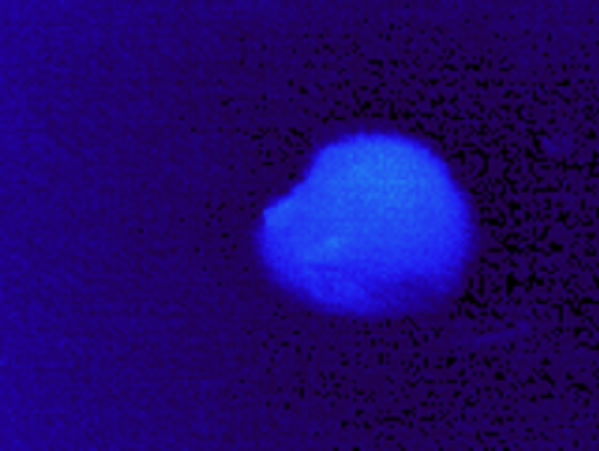

Supplement: Supplementary file 9 — Supplementary Slideshow S1.zip [file 41598_2018_30357_MOESM9_ESM.zip › Slideshow/photos/UTNB140427-057.jpg]

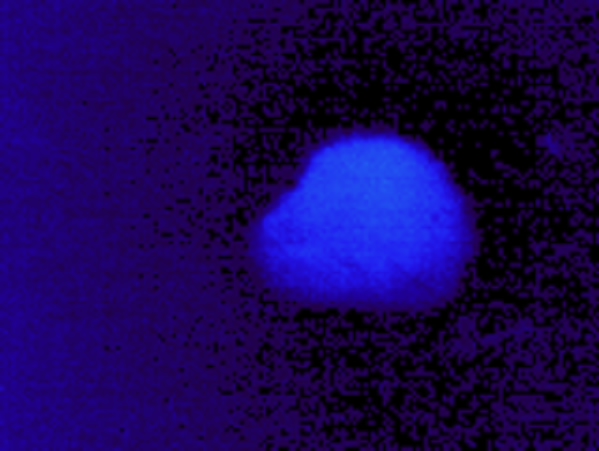

Supplement: Supplementary file 9 — Supplementary Slideshow S1.zip [file 41598_2018_30357_MOESM9_ESM.zip › Slideshow/photos/UTNB140427-043.jpg]

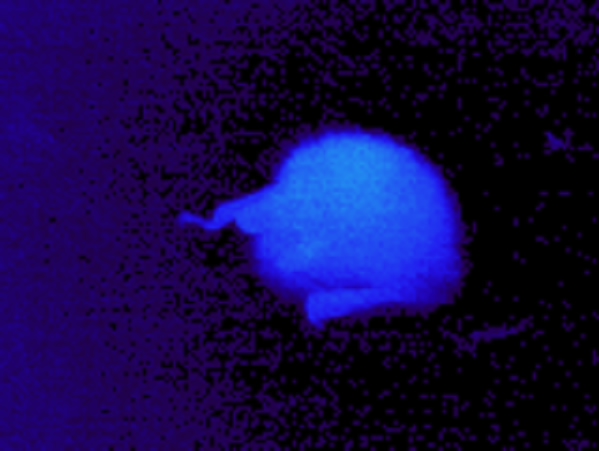

Supplement: Supplementary file 9 — Supplementary Slideshow S1.zip [file 41598_2018_30357_MOESM9_ESM.zip › Slideshow/photos/UTNB140427-094.jpg]

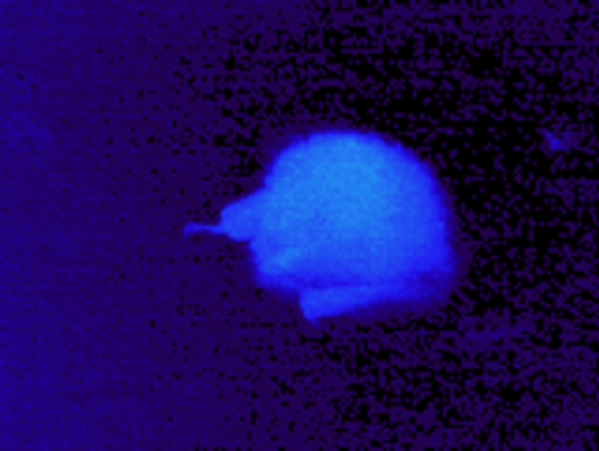

Supplement: Supplementary file 9 — Supplementary Slideshow S1.zip [file 41598_2018_30357_MOESM9_ESM.zip › Slideshow/photos/UTNB140427-080.jpg]

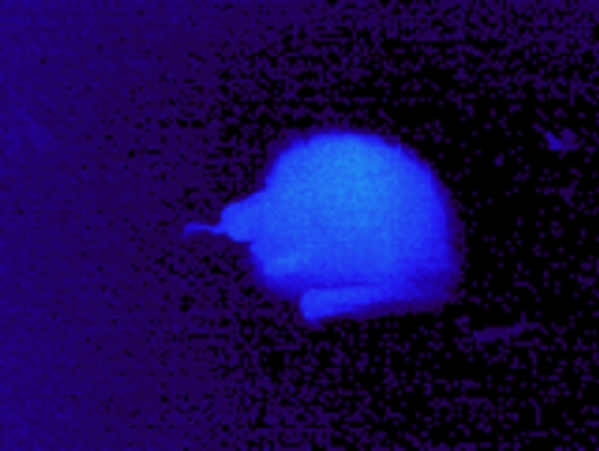

Supplement: Supplementary file 9 — Supplementary Slideshow S1.zip [file 41598_2018_30357_MOESM9_ESM.zip › Slideshow/photos/UTNB140427-081.jpg]

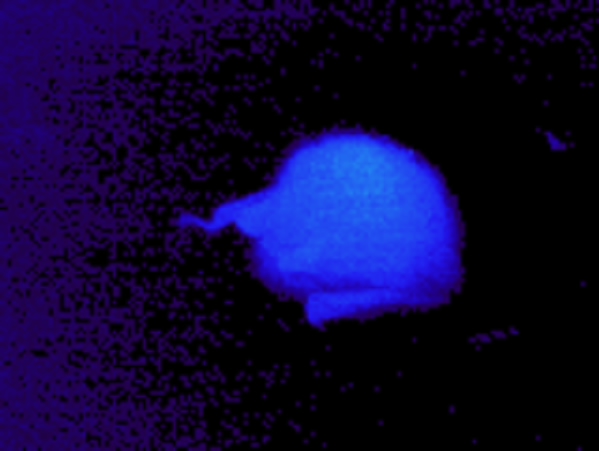

Supplement: Supplementary file 9 — Supplementary Slideshow S1.zip [file 41598_2018_30357_MOESM9_ESM.zip › Slideshow/photos/UTNB140427-095.jpg]

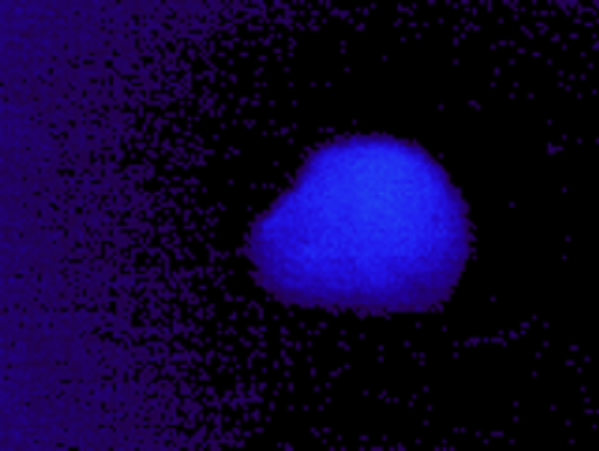

Supplement: Supplementary file 9 — Supplementary Slideshow S1.zip [file 41598_2018_30357_MOESM9_ESM.zip › Slideshow/photos/UTNB140427-042.jpg]

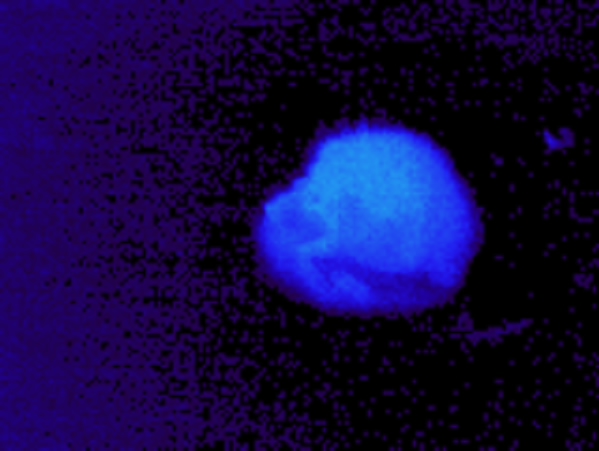

Supplement: Supplementary file 9 — Supplementary Slideshow S1.zip [file 41598_2018_30357_MOESM9_ESM.zip › Slideshow/photos/UTNB140427-056.jpg]

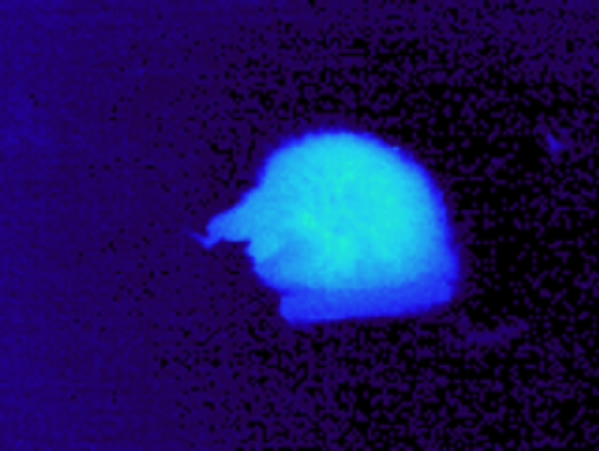

Supplement: Supplementary file 9 — Supplementary Slideshow S1.zip [file 41598_2018_30357_MOESM9_ESM.zip › Slideshow/photos/UTNB140427-136.jpg]

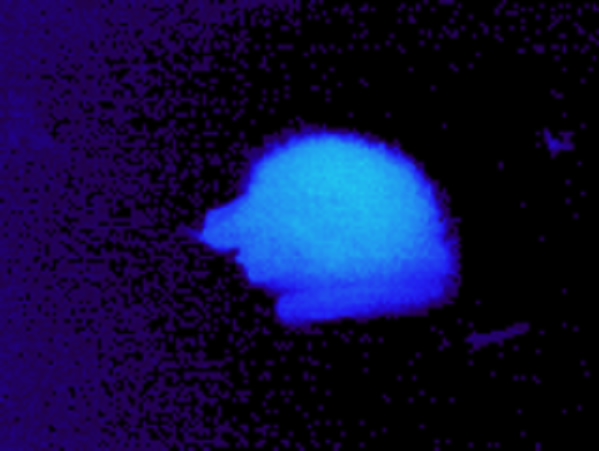

Supplement: Supplementary file 9 — Supplementary Slideshow S1.zip [file 41598_2018_30357_MOESM9_ESM.zip › Slideshow/photos/UTNB140427-122.jpg]

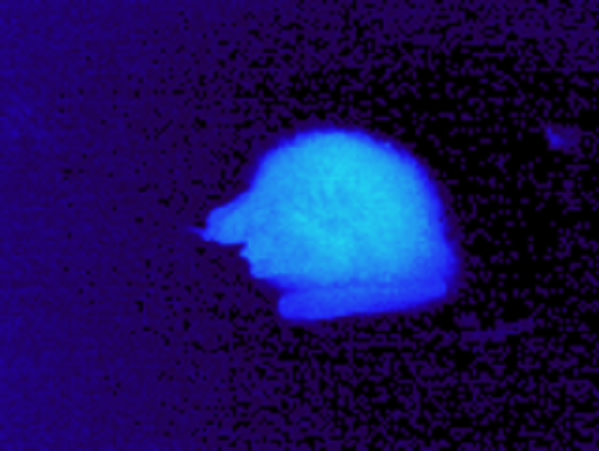

Supplement: Supplementary file 9 — Supplementary Slideshow S1.zip [file 41598_2018_30357_MOESM9_ESM.zip › Slideshow/photos/UTNB140427-126.jpg]

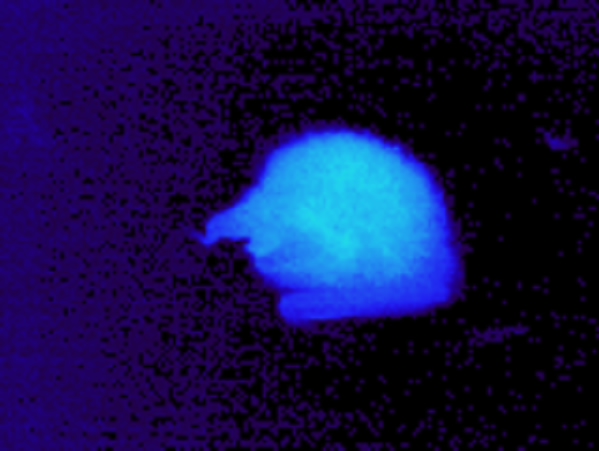

Supplement: Supplementary file 9 — Supplementary Slideshow S1.zip [file 41598_2018_30357_MOESM9_ESM.zip › Slideshow/photos/UTNB140427-132.jpg]

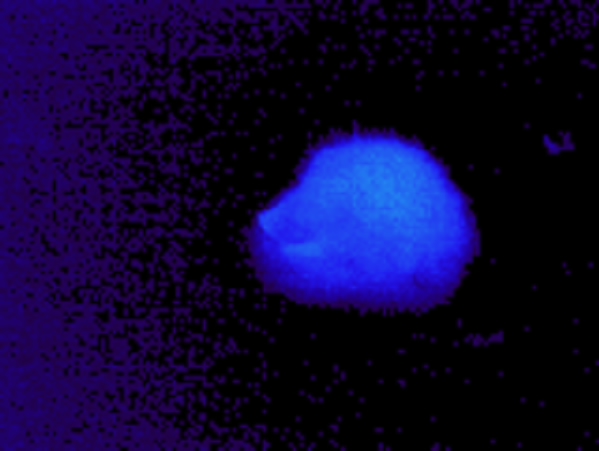

Supplement: Supplementary file 9 — Supplementary Slideshow S1.zip [file 41598_2018_30357_MOESM9_ESM.zip › Slideshow/photos/UTNB140427-052.jpg]

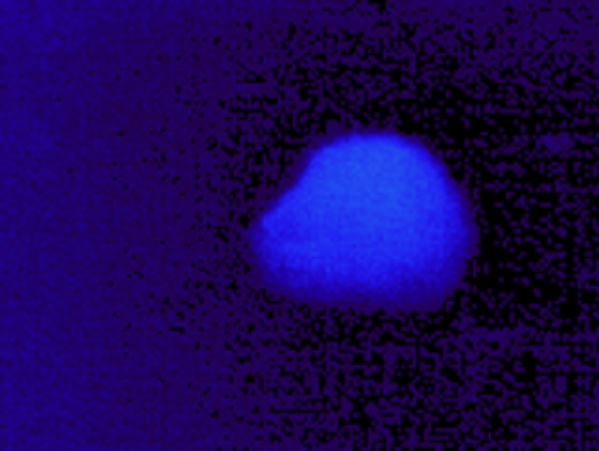

Supplement: Supplementary file 9 — Supplementary Slideshow S1.zip [file 41598_2018_30357_MOESM9_ESM.zip › Slideshow/photos/UTNB140427-046.jpg]

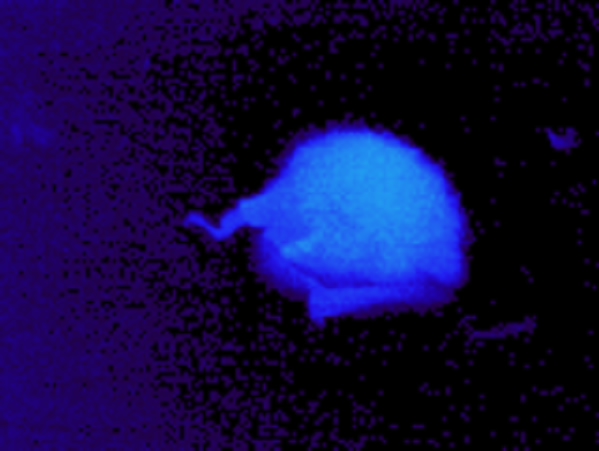

Supplement: Supplementary file 9 — Supplementary Slideshow S1.zip [file 41598_2018_30357_MOESM9_ESM.zip › Slideshow/photos/UTNB140427-091.jpg]

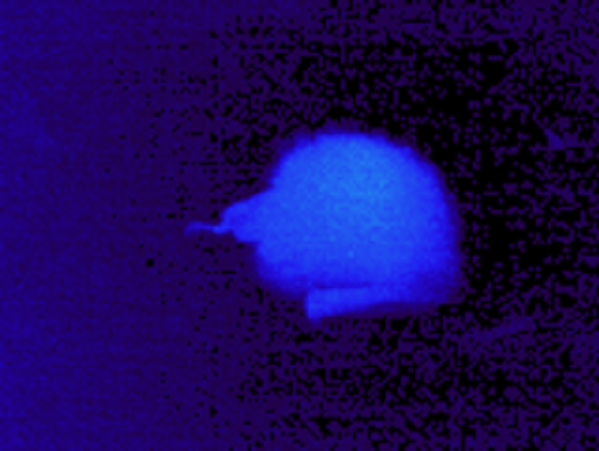

Supplement: Supplementary file 9 — Supplementary Slideshow S1.zip [file 41598_2018_30357_MOESM9_ESM.zip › Slideshow/photos/UTNB140427-085.jpg]

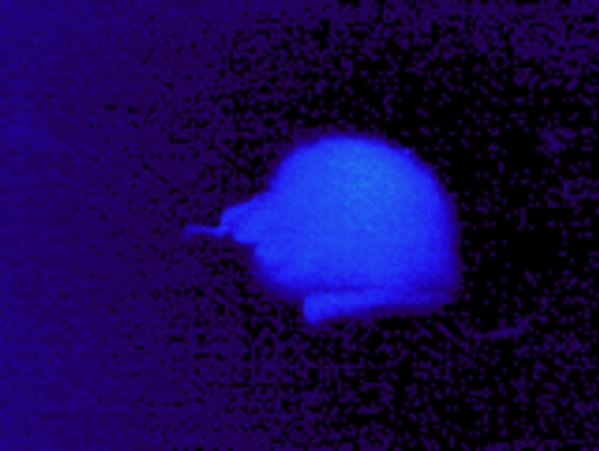

Supplement: Supplementary file 9 — Supplementary Slideshow S1.zip [file 41598_2018_30357_MOESM9_ESM.zip › Slideshow/photos/UTNB140427-084.jpg]

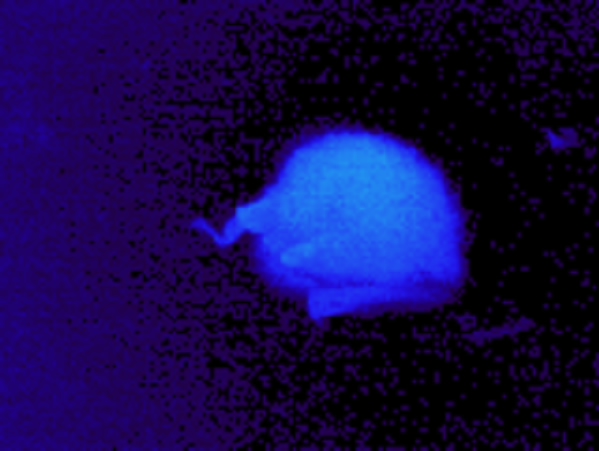

Supplement: Supplementary file 9 — Supplementary Slideshow S1.zip [file 41598_2018_30357_MOESM9_ESM.zip › Slideshow/photos/UTNB140427-090.jpg]

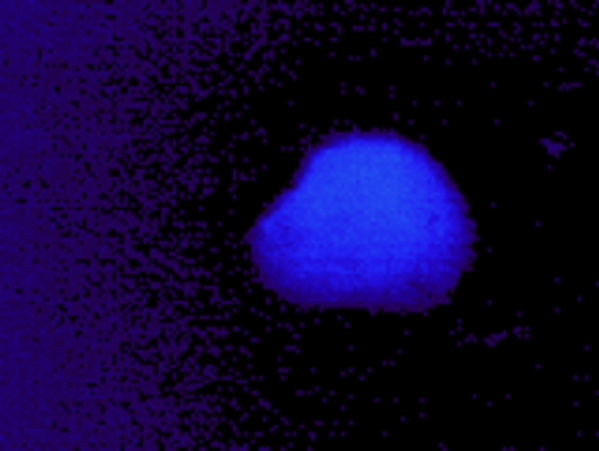

Supplement: Supplementary file 9 — Supplementary Slideshow S1.zip [file 41598_2018_30357_MOESM9_ESM.zip › Slideshow/photos/UTNB140427-047.jpg]

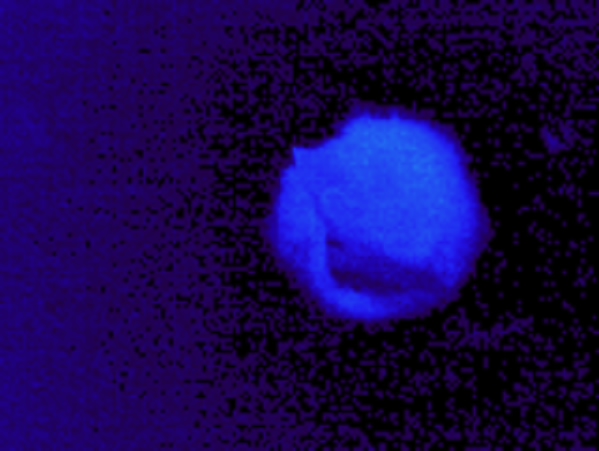

Supplement: Supplementary file 9 — Supplementary Slideshow S1.zip [file 41598_2018_30357_MOESM9_ESM.zip › Slideshow/photos/UTNB140427-053.jpg]

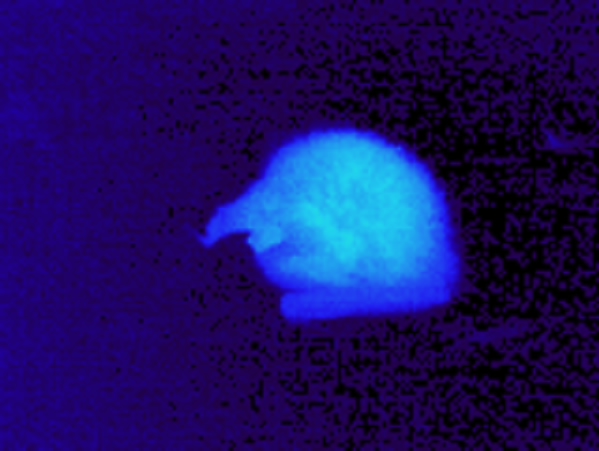

Supplement: Supplementary file 9 — Supplementary Slideshow S1.zip [file 41598_2018_30357_MOESM9_ESM.zip › Slideshow/photos/UTNB140427-133.jpg]

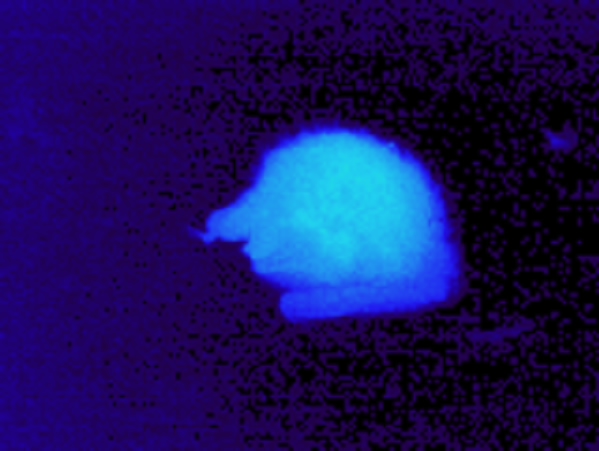

Supplement: Supplementary file 9 — Supplementary Slideshow S1.zip [file 41598_2018_30357_MOESM9_ESM.zip › Slideshow/photos/UTNB140427-127.jpg]

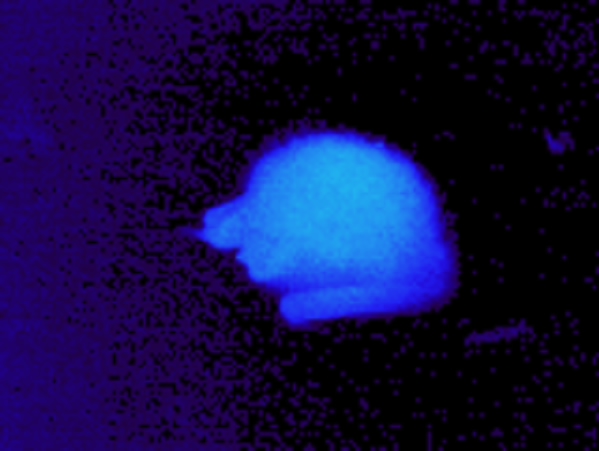

Supplement: Supplementary file 9 — Supplementary Slideshow S1.zip [file 41598_2018_30357_MOESM9_ESM.zip › Slideshow/photos/UTNB140427-119.jpg]

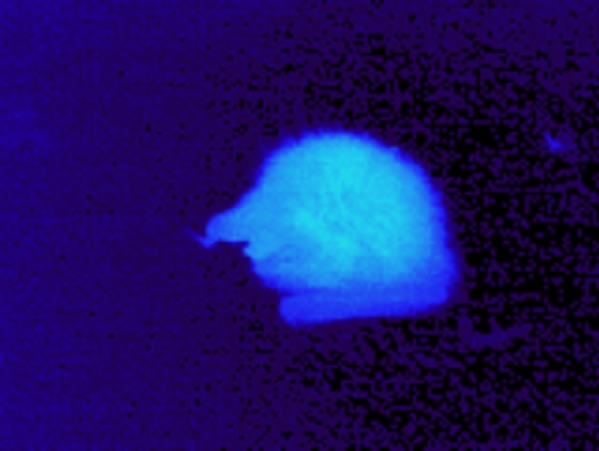

Supplement: Supplementary file 9 — Supplementary Slideshow S1.zip [file 41598_2018_30357_MOESM9_ESM.zip › Slideshow/photos/UTNB140427-131.jpg]

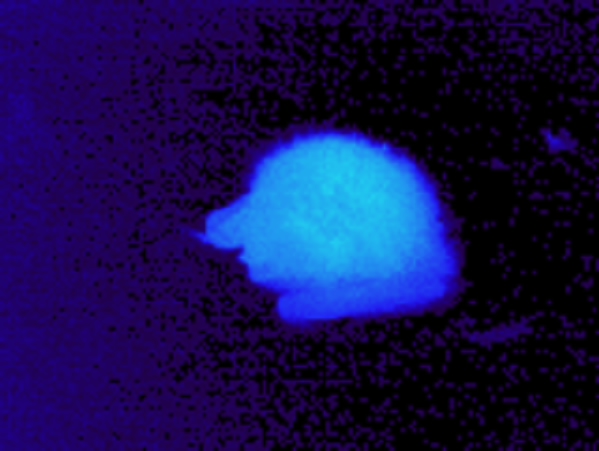

Supplement: Supplementary file 9 — Supplementary Slideshow S1.zip [file 41598_2018_30357_MOESM9_ESM.zip › Slideshow/photos/UTNB140427-125.jpg]

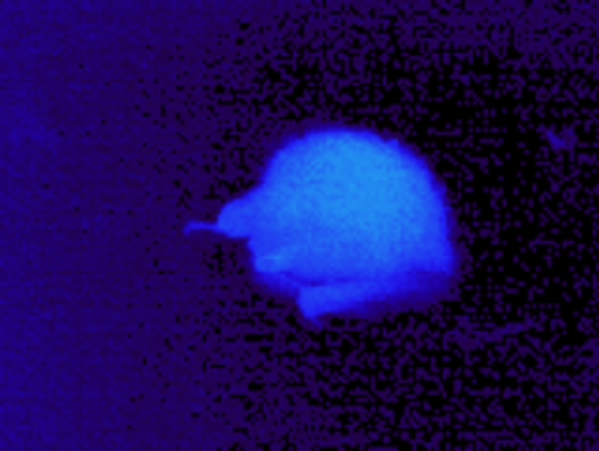

Supplement: Supplementary file 9 — Supplementary Slideshow S1.zip [file 41598_2018_30357_MOESM9_ESM.zip › Slideshow/photos/UTNB140427-079.jpg]

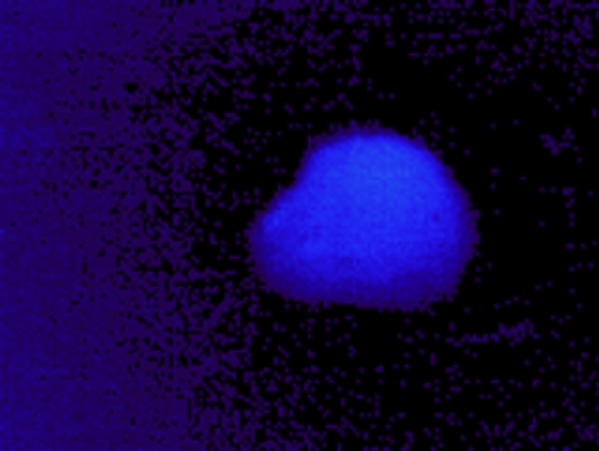

Supplement: Supplementary file 9 — Supplementary Slideshow S1.zip [file 41598_2018_30357_MOESM9_ESM.zip › Slideshow/photos/UTNB140427-045.jpg]

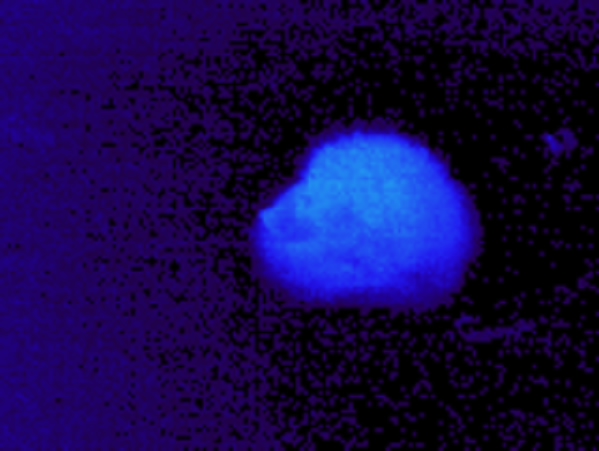

Supplement: Supplementary file 9 — Supplementary Slideshow S1.zip [file 41598_2018_30357_MOESM9_ESM.zip › Slideshow/photos/UTNB140427-051.jpg]

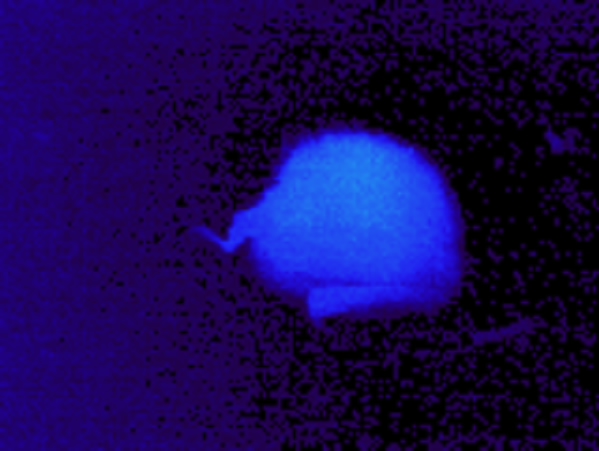

Supplement: Supplementary file 9 — Supplementary Slideshow S1.zip [file 41598_2018_30357_MOESM9_ESM.zip › Slideshow/photos/UTNB140427-086.jpg]

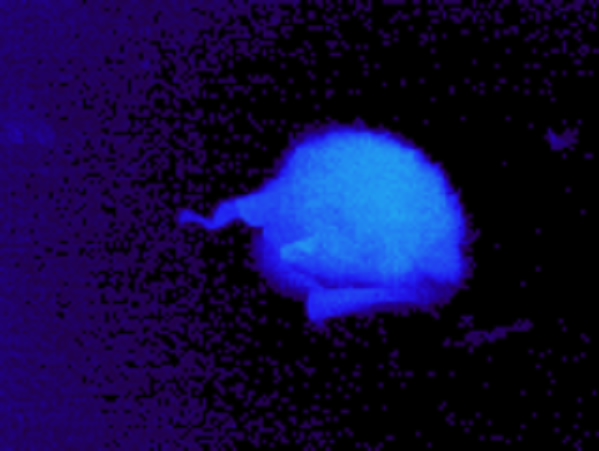

Supplement: Supplementary file 9 — Supplementary Slideshow S1.zip [file 41598_2018_30357_MOESM9_ESM.zip › Slideshow/photos/UTNB140427-092.jpg]

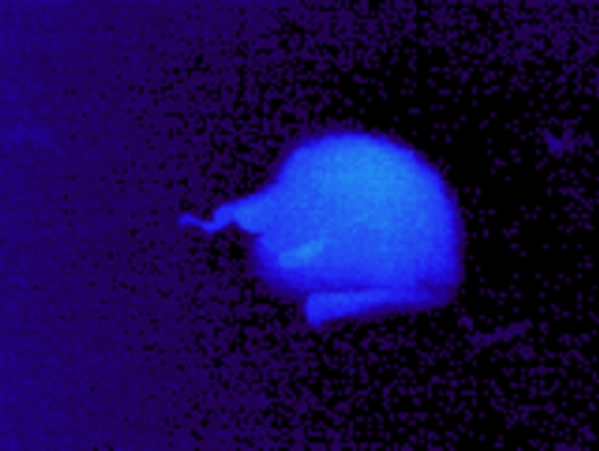

Supplement: Supplementary file 9 — Supplementary Slideshow S1.zip [file 41598_2018_30357_MOESM9_ESM.zip › Slideshow/photos/UTNB140427-093.jpg]

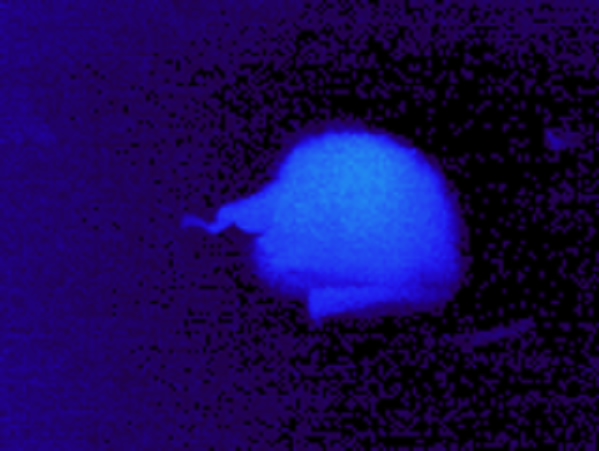

Supplement: Supplementary file 9 — Supplementary Slideshow S1.zip [file 41598_2018_30357_MOESM9_ESM.zip › Slideshow/photos/UTNB140427-087.jpg]

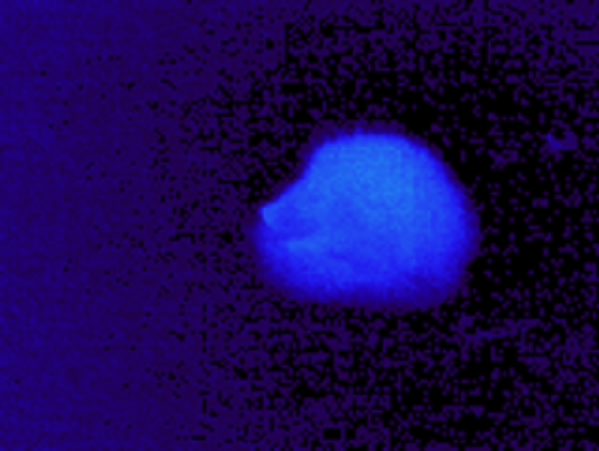

Supplement: Supplementary file 9 — Supplementary Slideshow S1.zip [file 41598_2018_30357_MOESM9_ESM.zip › Slideshow/photos/UTNB140427-050.jpg]

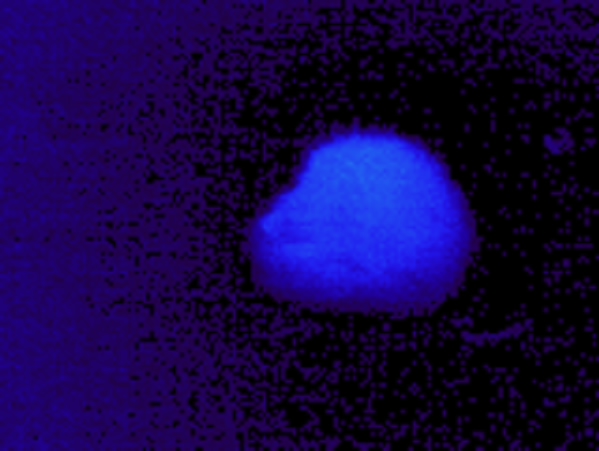

Supplement: Supplementary file 9 — Supplementary Slideshow S1.zip [file 41598_2018_30357_MOESM9_ESM.zip › Slideshow/photos/UTNB140427-044.jpg]

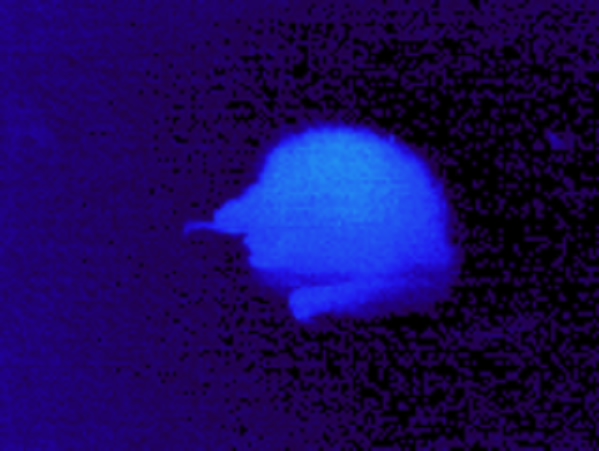

Supplement: Supplementary file 9 — Supplementary Slideshow S1.zip [file 41598_2018_30357_MOESM9_ESM.zip › Slideshow/photos/UTNB140427-078.jpg]

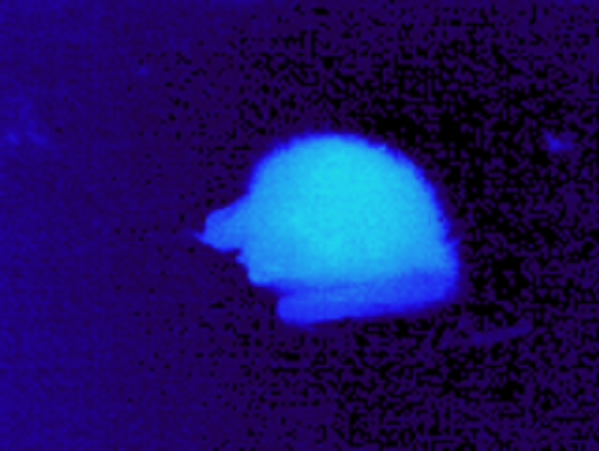

Supplement: Supplementary file 9 — Supplementary Slideshow S1.zip [file 41598_2018_30357_MOESM9_ESM.zip › Slideshow/photos/UTNB140427-124.jpg]

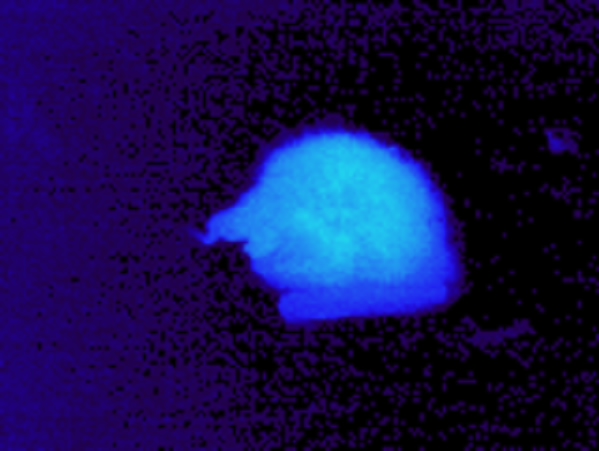

Supplement: Supplementary file 9 — Supplementary Slideshow S1.zip [file 41598_2018_30357_MOESM9_ESM.zip › Slideshow/photos/UTNB140427-130.jpg]

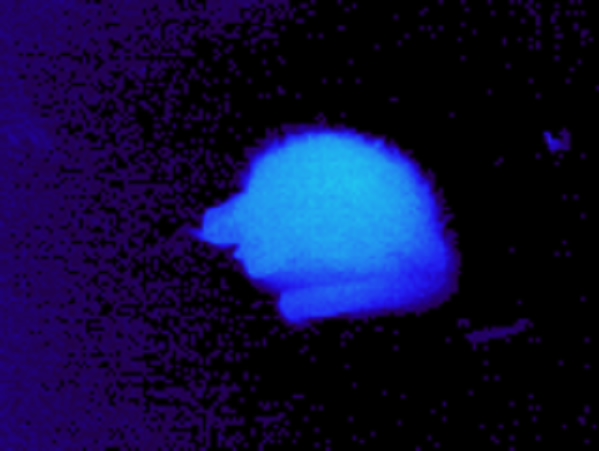

Supplement: Supplementary file 9 — Supplementary Slideshow S1.zip [file 41598_2018_30357_MOESM9_ESM.zip › Slideshow/photos/UTNB140427-118.jpg]

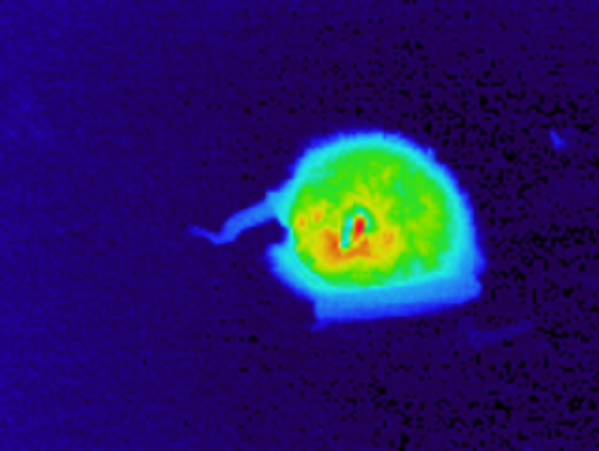

Supplement: Supplementary file 9 — Supplementary Slideshow S1.zip [file 41598_2018_30357_MOESM9_ESM.zip › Slideshow/photos/UTNB140427-157.jpg]

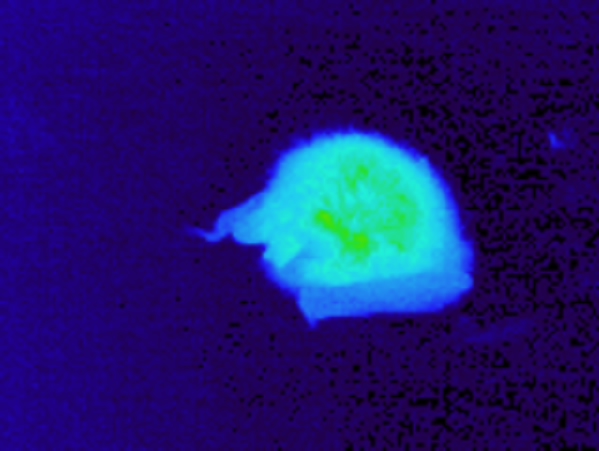

Supplement: Supplementary file 9 — Supplementary Slideshow S1.zip [file 41598_2018_30357_MOESM9_ESM.zip › Slideshow/photos/UTNB140427-143.jpg]

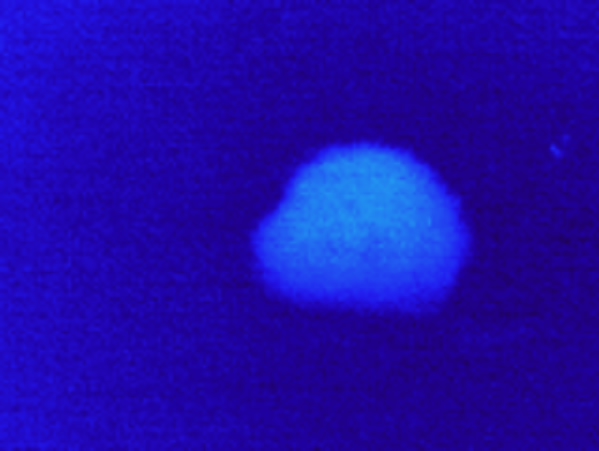

Supplement: Supplementary file 9 — Supplementary Slideshow S1.zip [file 41598_2018_30357_MOESM9_ESM.zip › Slideshow/photos/UTNB140427-023.jpg]

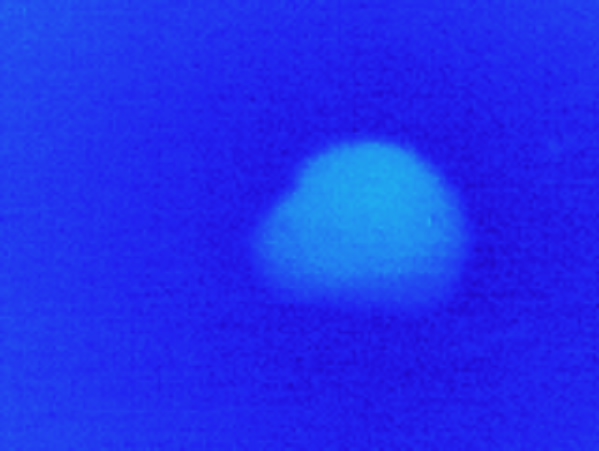

Supplement: Supplementary file 9 — Supplementary Slideshow S1.zip [file 41598_2018_30357_MOESM9_ESM.zip › Slideshow/photos/UTNB140427-037.jpg]

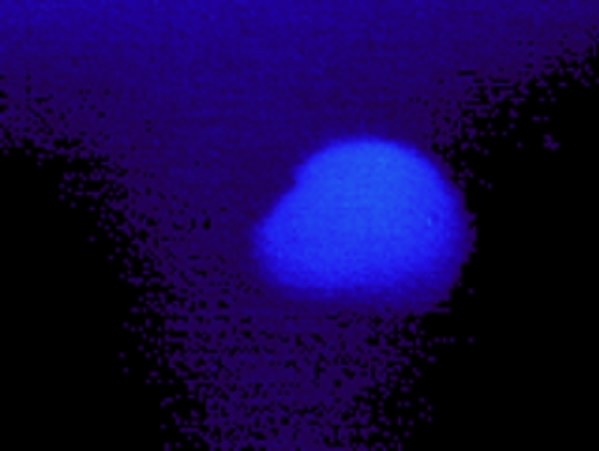

Supplement: Supplementary file 9 — Supplementary Slideshow S1.zip [file 41598_2018_30357_MOESM9_ESM.zip › Slideshow/photos/UTNB140427-036.jpg]

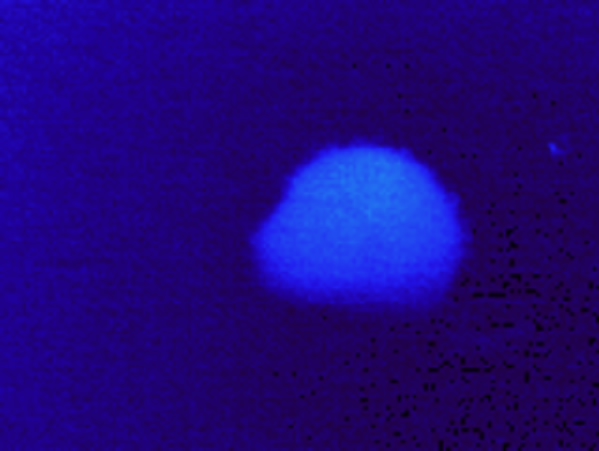

Supplement: Supplementary file 9 — Supplementary Slideshow S1.zip [file 41598_2018_30357_MOESM9_ESM.zip › Slideshow/photos/UTNB140427-022.jpg]

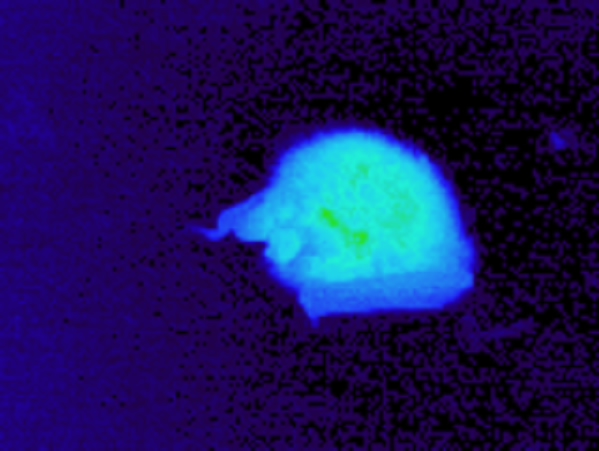

Supplement: Supplementary file 9 — Supplementary Slideshow S1.zip [file 41598_2018_30357_MOESM9_ESM.zip › Slideshow/photos/UTNB140427-142.jpg]

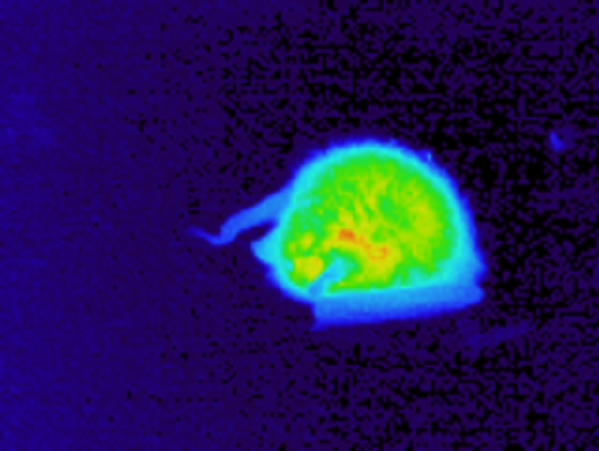

Supplement: Supplementary file 9 — Supplementary Slideshow S1.zip [file 41598_2018_30357_MOESM9_ESM.zip › Slideshow/photos/UTNB140427-156.jpg]

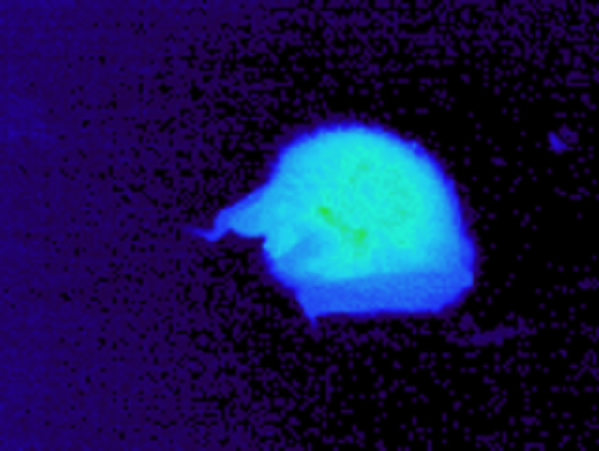

Supplement: Supplementary file 9 — Supplementary Slideshow S1.zip [file 41598_2018_30357_MOESM9_ESM.zip › Slideshow/photos/UTNB140427-140.jpg]

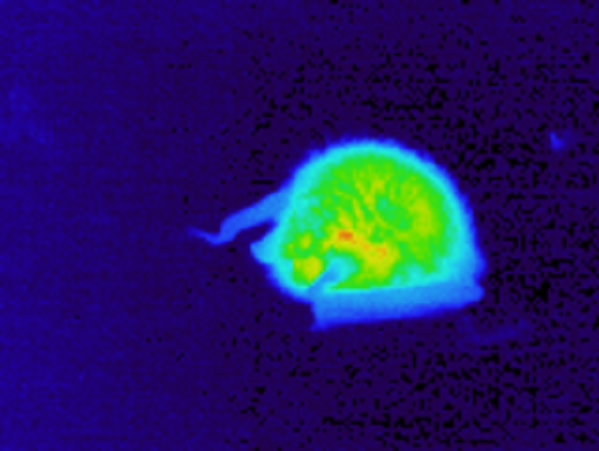

Supplement: Supplementary file 9 — Supplementary Slideshow S1.zip [file 41598_2018_30357_MOESM9_ESM.zip › Slideshow/photos/UTNB140427-154.jpg]

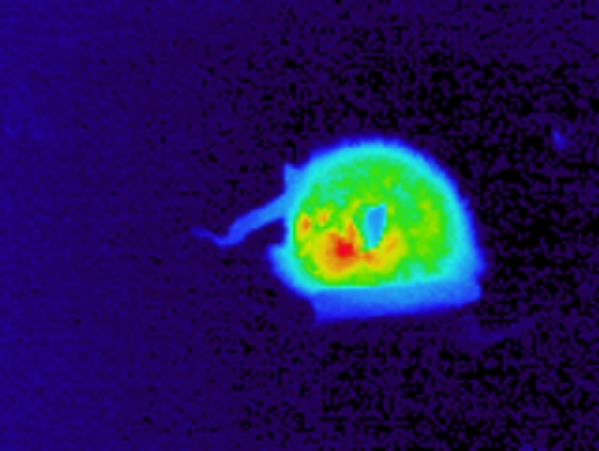

Supplement: Supplementary file 9 — Supplementary Slideshow S1.zip [file 41598_2018_30357_MOESM9_ESM.zip › Slideshow/photos/UTNB140427-168.jpg]

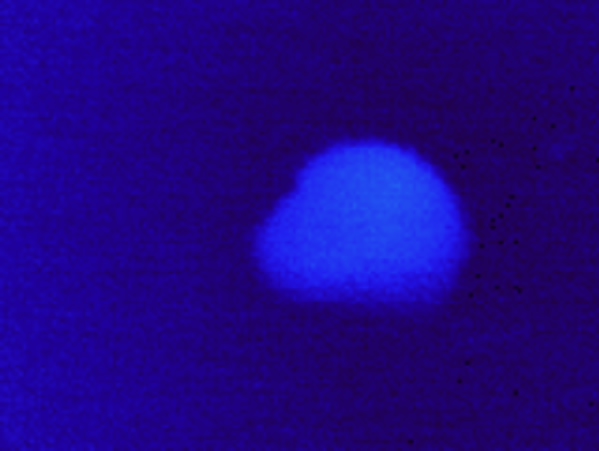

Supplement: Supplementary file 9 — Supplementary Slideshow S1.zip [file 41598_2018_30357_MOESM9_ESM.zip › Slideshow/photos/UTNB140427-034.jpg]

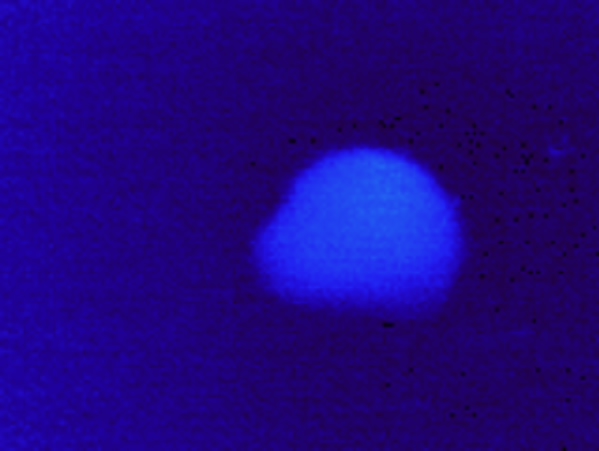

Supplement: Supplementary file 9 — Supplementary Slideshow S1.zip [file 41598_2018_30357_MOESM9_ESM.zip › Slideshow/photos/UTNB140427-020.jpg]

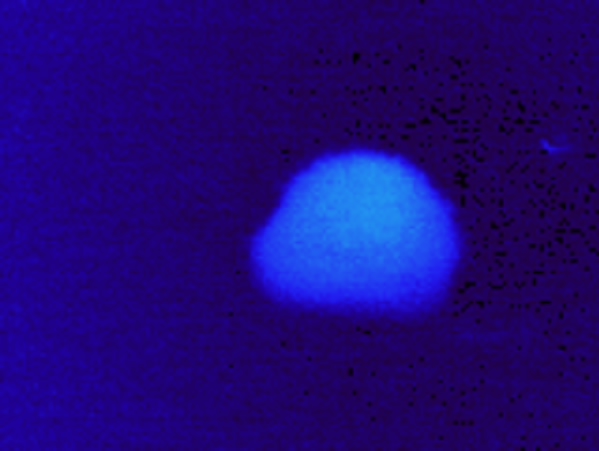

Supplement: Supplementary file 9 — Supplementary Slideshow S1.zip [file 41598_2018_30357_MOESM9_ESM.zip › Slideshow/photos/UTNB140427-008.jpg]

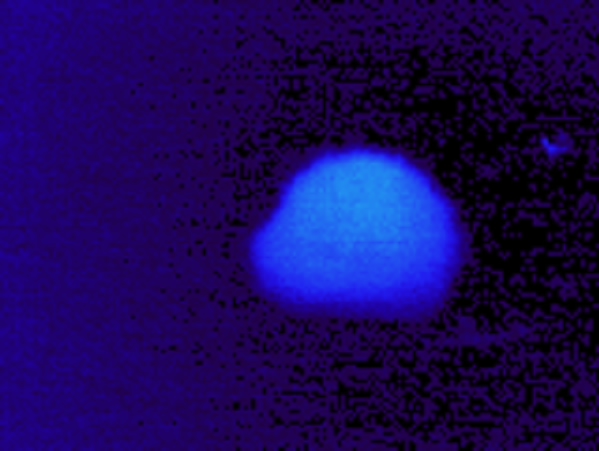

Supplement: Supplementary file 9 — Supplementary Slideshow S1.zip [file 41598_2018_30357_MOESM9_ESM.zip › Slideshow/photos/UTNB140427-009.jpg]

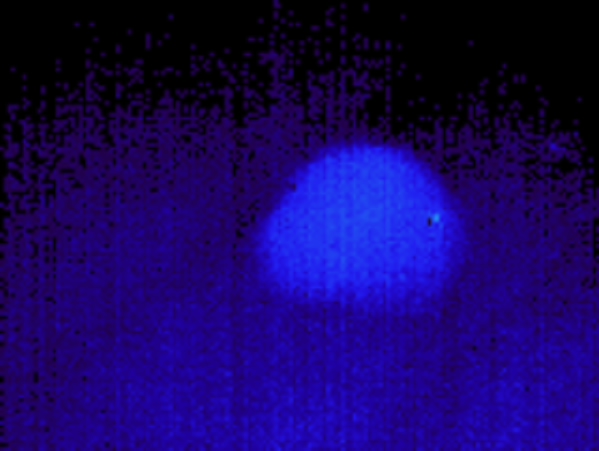

Supplement: Supplementary file 9 — Supplementary Slideshow S1.zip [file 41598_2018_30357_MOESM9_ESM.zip › Slideshow/photos/UTNB140427-021.jpg]

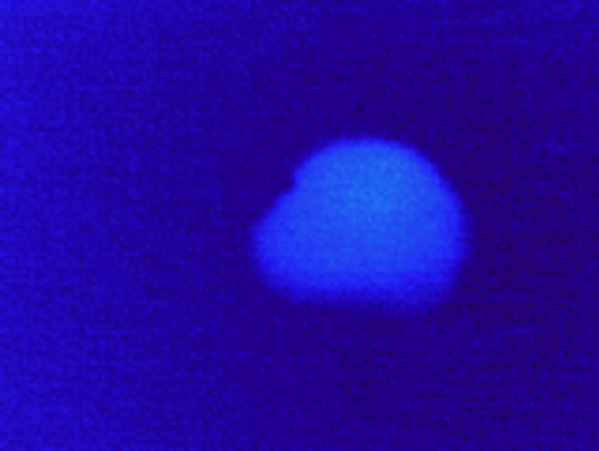

Supplement: Supplementary file 9 — Supplementary Slideshow S1.zip [file 41598_2018_30357_MOESM9_ESM.zip › Slideshow/photos/UTNB140427-035.jpg]

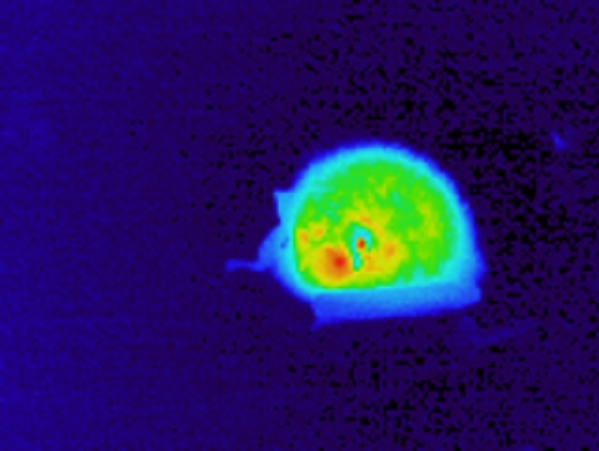

Supplement: Supplementary file 9 — Supplementary Slideshow S1.zip [file 41598_2018_30357_MOESM9_ESM.zip › Slideshow/photos/UTNB140427-169.jpg]

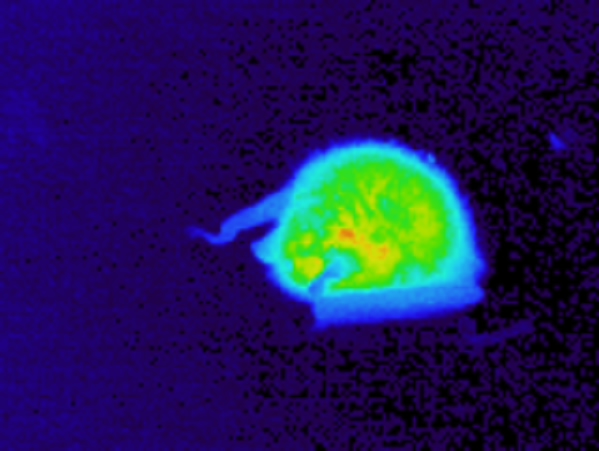

Supplement: Supplementary file 9 — Supplementary Slideshow S1.zip [file 41598_2018_30357_MOESM9_ESM.zip › Slideshow/photos/UTNB140427-155.jpg]

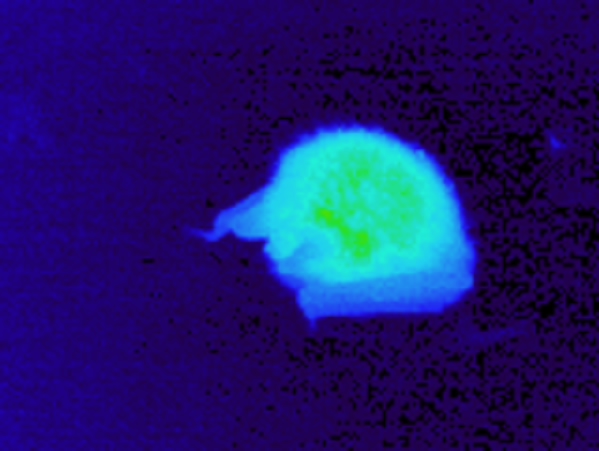

Supplement: Supplementary file 9 — Supplementary Slideshow S1.zip [file 41598_2018_30357_MOESM9_ESM.zip › Slideshow/photos/UTNB140427-141.jpg]

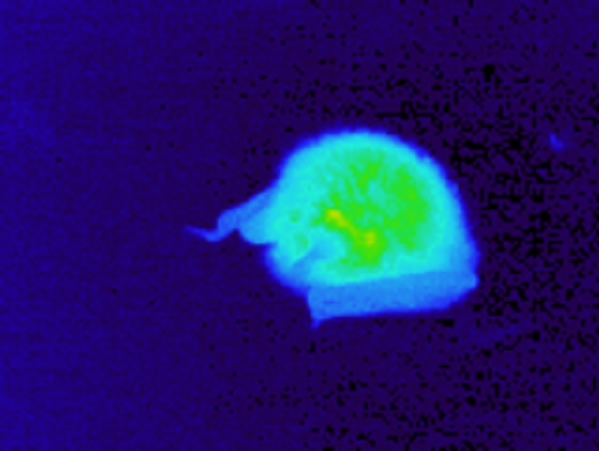

Supplement: Supplementary file 9 — Supplementary Slideshow S1.zip [file 41598_2018_30357_MOESM9_ESM.zip › Slideshow/photos/UTNB140427-145.jpg]

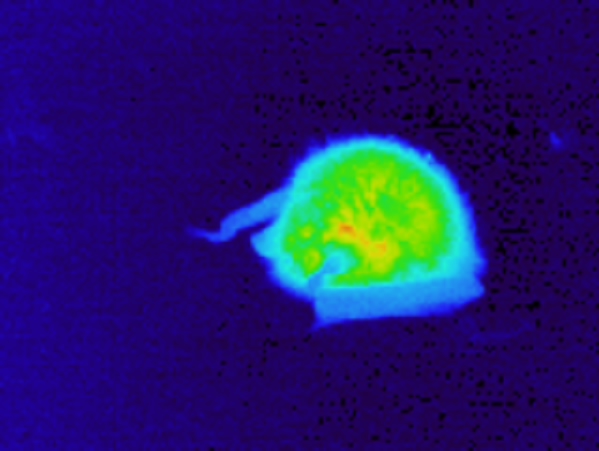

Supplement: Supplementary file 9 — Supplementary Slideshow S1.zip [file 41598_2018_30357_MOESM9_ESM.zip › Slideshow/photos/UTNB140427-151.jpg]

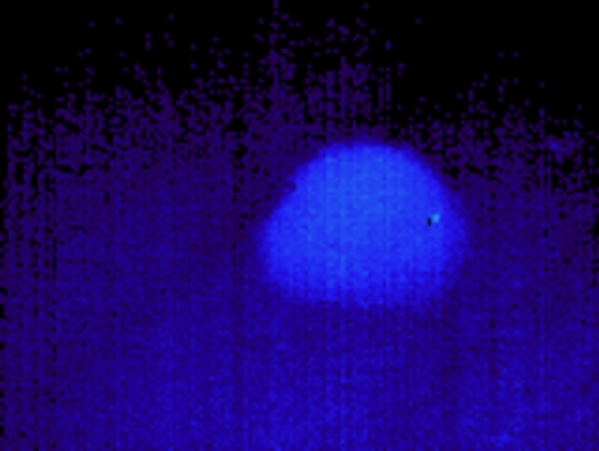

Supplement: Supplementary file 9 — Supplementary Slideshow S1.zip [file 41598_2018_30357_MOESM9_ESM.zip › Slideshow/photos/UTNB140427-019.jpg]

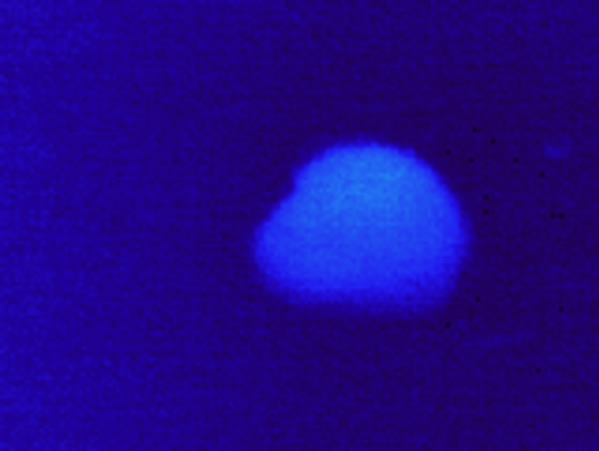

Supplement: Supplementary file 9 — Supplementary Slideshow S1.zip [file 41598_2018_30357_MOESM9_ESM.zip › Slideshow/photos/UTNB140427-031.jpg]

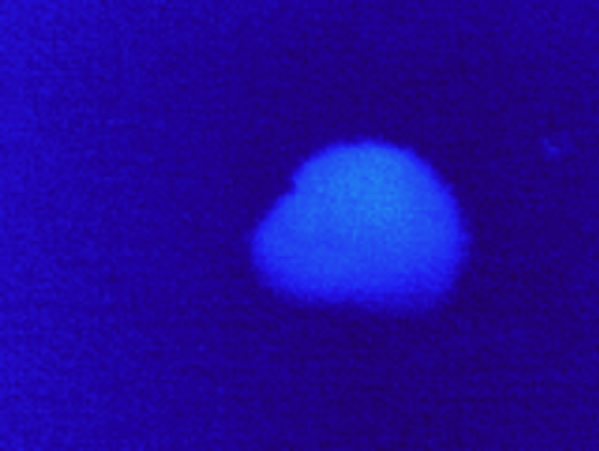

Supplement: Supplementary file 9 — Supplementary Slideshow S1.zip [file 41598_2018_30357_MOESM9_ESM.zip › Slideshow/photos/UTNB140427-025.jpg]

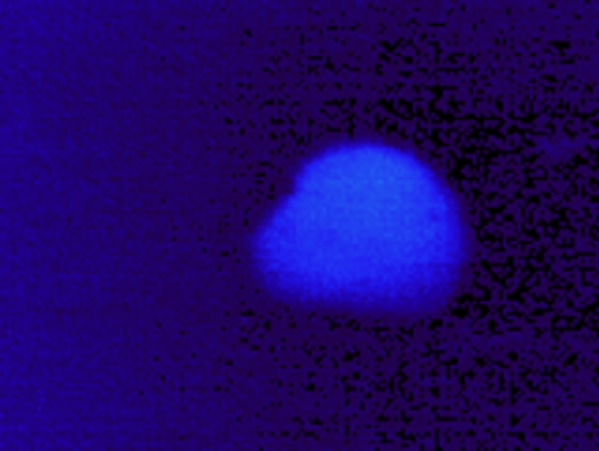

Supplement: Supplementary file 9 — Supplementary Slideshow S1.zip [file 41598_2018_30357_MOESM9_ESM.zip › Slideshow/photos/UTNB140427-024.jpg]

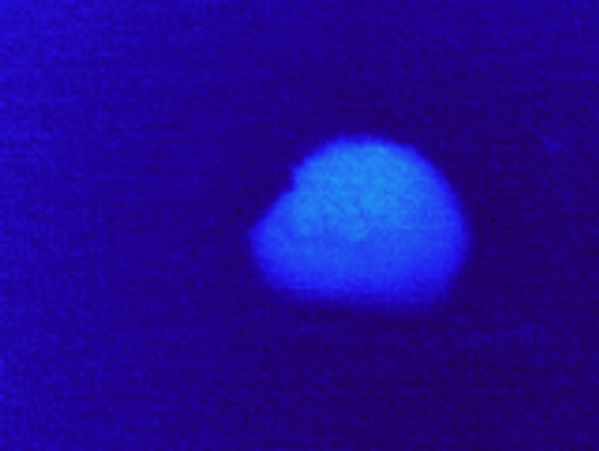

Supplement: Supplementary file 9 — Supplementary Slideshow S1.zip [file 41598_2018_30357_MOESM9_ESM.zip › Slideshow/photos/UTNB140427-030.jpg]

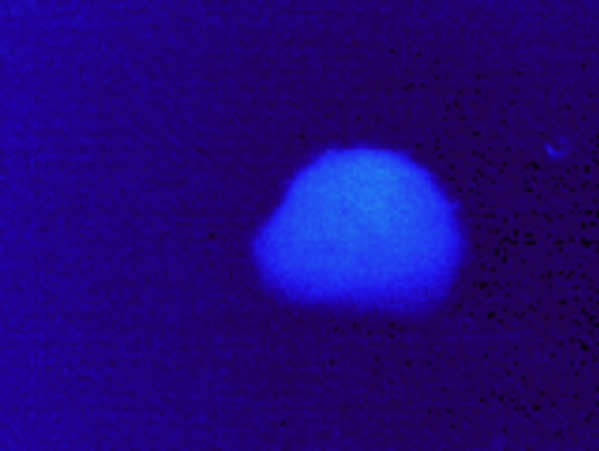

Supplement: Supplementary file 9 — Supplementary Slideshow S1.zip [file 41598_2018_30357_MOESM9_ESM.zip › Slideshow/photos/UTNB140427-018.jpg]

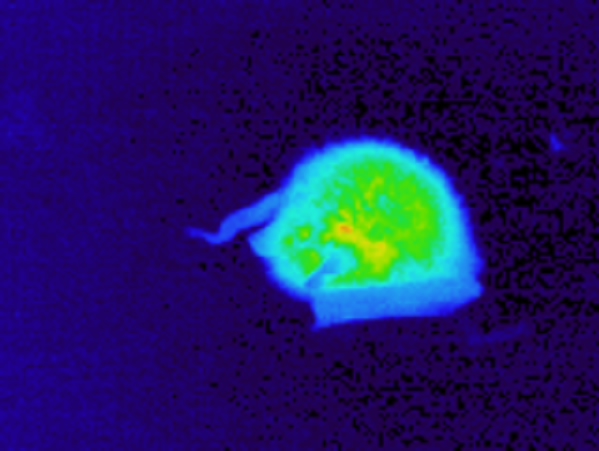

Supplement: Supplementary file 9 — Supplementary Slideshow S1.zip [file 41598_2018_30357_MOESM9_ESM.zip › Slideshow/photos/UTNB140427-150.jpg]

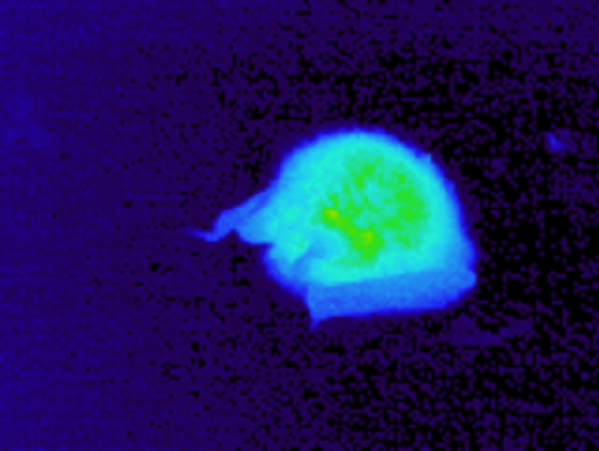

Supplement: Supplementary file 9 — Supplementary Slideshow S1.zip [file 41598_2018_30357_MOESM9_ESM.zip › Slideshow/photos/UTNB140427-144.jpg]

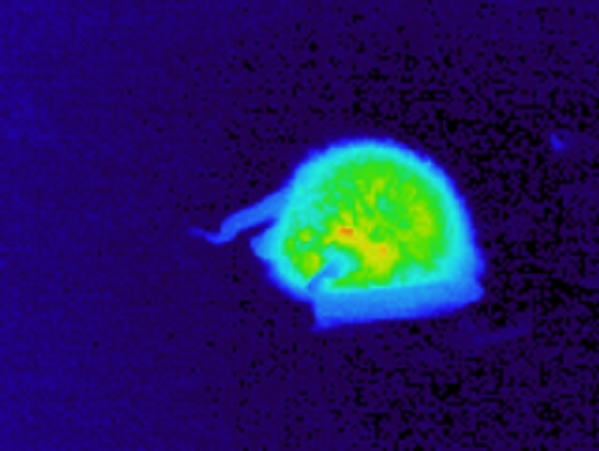

Supplement: Supplementary file 9 — Supplementary Slideshow S1.zip [file 41598_2018_30357_MOESM9_ESM.zip › Slideshow/photos/UTNB140427-152.jpg]

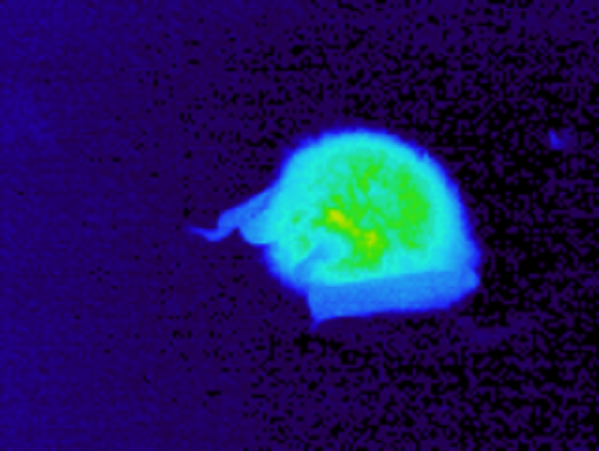

Supplement: Supplementary file 9 — Supplementary Slideshow S1.zip [file 41598_2018_30357_MOESM9_ESM.zip › Slideshow/photos/UTNB140427-146.jpg]

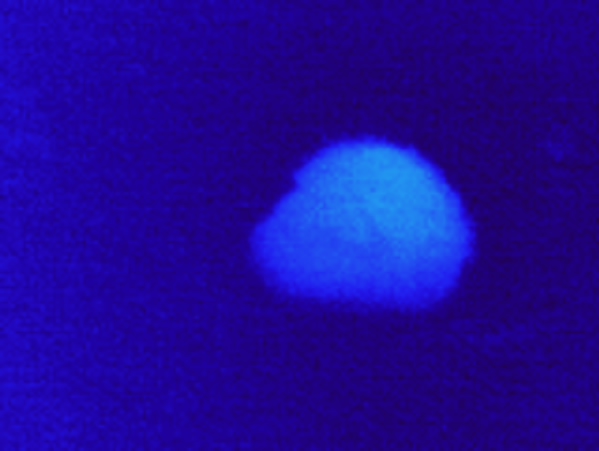

Supplement: Supplementary file 9 — Supplementary Slideshow S1.zip [file 41598_2018_30357_MOESM9_ESM.zip › Slideshow/photos/UTNB140427-032.jpg]

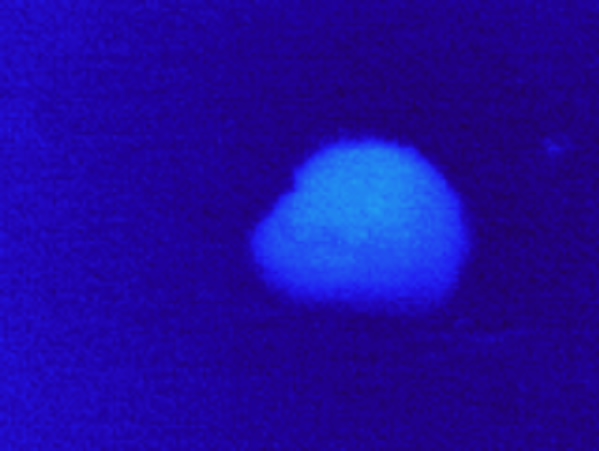

Supplement: Supplementary file 9 — Supplementary Slideshow S1.zip [file 41598_2018_30357_MOESM9_ESM.zip › Slideshow/photos/UTNB140427-033.jpg]

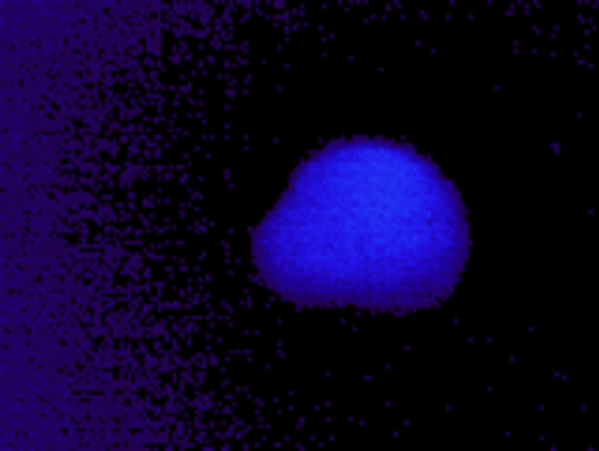

Supplement: Supplementary file 9 — Supplementary Slideshow S1.zip [file 41598_2018_30357_MOESM9_ESM.zip › Slideshow/photos/UTNB140427-027.jpg]

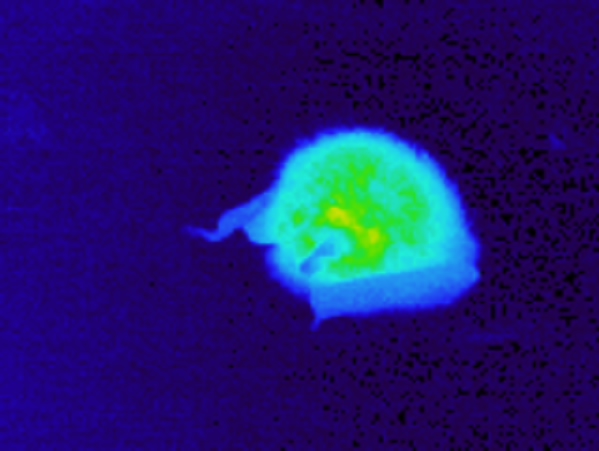

Supplement: Supplementary file 9 — Supplementary Slideshow S1.zip [file 41598_2018_30357_MOESM9_ESM.zip › Slideshow/photos/UTNB140427-147.jpg]

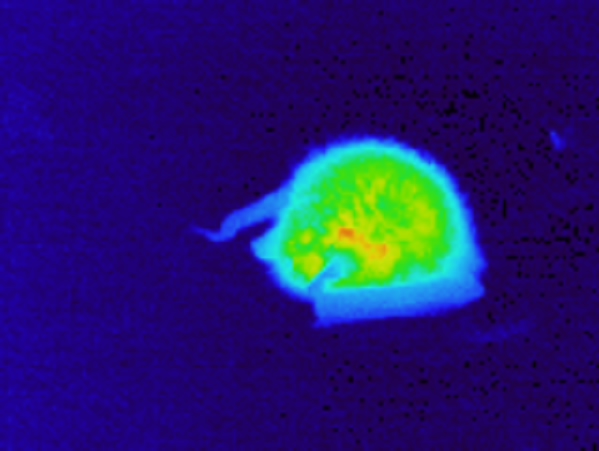

Supplement: Supplementary file 9 — Supplementary Slideshow S1.zip [file 41598_2018_30357_MOESM9_ESM.zip › Slideshow/photos/UTNB140427-153.jpg]

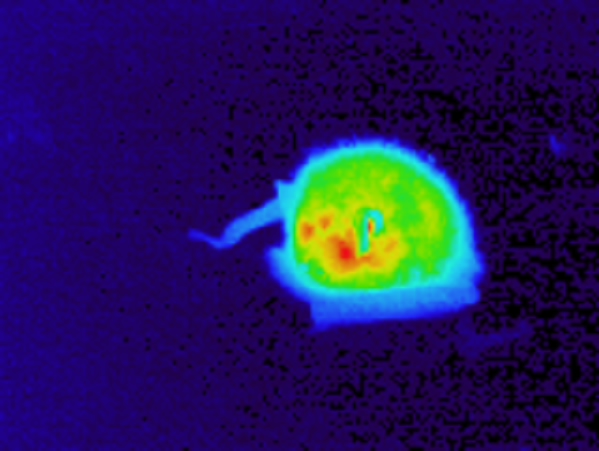

Supplement: Supplementary file 9 — Supplementary Slideshow S1.zip [file 41598_2018_30357_MOESM9_ESM.zip › Slideshow/photos/UTNB140427-162.jpg]
